# Deciphering the global roles of Cold shock proteins in *Listeria monocytogenes* nutrient metabolism and stress tolerance

**DOI:** 10.3389/fmicb.2022.1057754

**Published:** 2022-12-20

**Authors:** Francis Muchaamba, Ueli von Ah, Roger Stephan, Marc J. A. Stevens, Taurai Tasara

**Affiliations:** ^1^Institute for Food Safety and Hygiene, Vetsuisse Faculty, University of Zurich, Zurich, Switzerland; ^2^Agroscope, Bern, Switzerland

**Keywords:** *Listeria monocytogenes*, cold shock protein, phenotype, regulon, stress tolerance, nutrient utilization

## Abstract

*Listeria monocytogenes* (*Lm*) accounts for serious public health and food safety problems owing to its stress resilience and pathogenicity. Based on their regulatory involvement in global gene expression events, cold-shock domain family proteins (Csps) are crucial in expression of various stress fitness and virulence phenotypes in bacteria. *Lm* possesses three Csps (CspA, CspB, and CspD) whose regulatory roles in the context of the genetic diversity of this bacterium are not yet fully understood. We examined the impacts of Csps deficiency on *Lm* nutrient metabolism and stress tolerance using a set of *csp* deletion mutants generated in different genetic backgrounds. Phenotype microarrays (PM) analysis showed that the absence of Csps in ∆*cspABD* reduces carbon (C-) source utilization capacity and increases *Lm* sensitivity to osmotic, pH, various chemical, and antimicrobial stress conditions. Single and double *csp* deletion mutants in different *Lm* genetic backgrounds were used to further dissect the roles of individual Csps in these phenotypes. Selected PM-based observations were further corroborated through targeted phenotypic assays, confirming that Csps are crucial in *Lm* for optimal utilization of various C-sources including rhamnose and glucose as well as tolerance against NaCl, β-phenyethylamine (PEA), and food relevant detergent stress conditions. Strain and genetic lineage background-based differences, division of labour, epistasis, and functional redundancies among the Csps were uncovered with respect to their roles in various processes including C-source utilization, cold, and PEA stress resistance. Finally, targeted transcriptome analysis was performed, revealing the activation of *csp* gene expression under defined stress conditions and the impact of Csps on expression regulation of selected rhamnose utilization genes. Overall, our study shows that Csps play important roles in nutrient utilization and stress responses in *Lm* strains, contributing to traits that are central to the public health and food safety impacts of this pathogen.

## Introduction

*Listeria monocytogenes* (*Lm*) the causative agent of listeriosis is an important foodborne pathogen that accounts for serious public health problems and food safety challenges by causing severe clinical illnesses and high mortality rates in vulnerable human populations (European Food Safety Authority [EFSA], 2017; Centers for Disease Control [CDC], 2018; Radoshevich and Cossart, 2018). Major outbreaks, continuous sporadic cases, and food recalls highlight the public health significance of this pathogen (Buchanan et al., 2017; Smith et al., 2019). *Lm* is genetically diverse comprising four main genetic lineages, a hybrid sub-lineage, and numerous multi-locus sequence types (Maury et al., 2016; Painset et al., 2019; Yin et al., 2019). Epidemiological distribution of different *Lm* strains with respect to food, processing environments, and clinical listeriosis cases follow a genetic background-associated trend (Doumith et al., 2004; Maury et al., 2016; Datta and Burall, 2018; Fritsch et al., 2018). Lineage I (LI) serotype 4b clonal complex CC1, CC2, CC4, and CC6 strains shown to be highly virulent through *in vivo* virulence studies are overrepresented in clinical cases. Lineage II (LII) serotype 1/2a CC8, CC9, and CC121 strains that often predominate among food and associated environmental isolates have been found to be less virulent (Maury et al., 2016; Datta and Burall, 2018; Lee et al., 2019; Muchaamba et al., 2022).

The ubiquitous distribution of *Lm* in addition to its virulence, increased tolerance to several hurdle techniques used in food production, and ability to grow at refrigeration temperatures contribute to its public health and food safety challenges (Gandhi and Chikindas, 2007; Cossart, 2011; Bucur et al., 2018; Wiktorczyk-Kapischke et al., 2021). These attributes depend on *Lm* deploying various protein systems that ensure appropriately regulated expression of stress adaption and virulence responses. This allows *Lm* to survive, thrive, and passage through the food chain overcoming hurdle procedures, ultimately causing host infection and disease (Gandhi and Chikindas, 2007; Burgess et al., 2016; Wiktorczyk-Kapischke et al., 2021). Genes encoding proteins that promote *Lm* stress tolerance, nutrient utilization, and virulence are under tight regulation. Gene expression regulatory proteins in this foodborne pathogen include alternative sigma factors and cold-shock domain family proteins (Csps; Nadon et al., 2002; O’Byrne and Karatzas, 2008; Radoshevich and Cossart, 2018; Gaballa et al., 2019; Muchaamba et al., 2021a). Two-component systems, regulatory RNA, and other gene expression regulatory mechanisms all act in parallel to fine-tune the expression of these stress tolerance and virulence-conferring systems (De Las Heras et al., 2011; Cerutti et al., 2017; Lebreton and Cossart, 2017; Radoshevich and Cossart, 2018). However, knowledge of how some of these regulators function is limited, yet it is pivotal to fully understand *Lm* public health and food safety relevant traits.

Csps are small, highly conserved nucleic acid binding proteins that regulate the expression of several genes (Horn et al., 2007; Keto-Timonen et al., 2016; Caballero et al., 2018). Named due to their induction in response to rapid downshift from optimum to cold temperature, i.e., cold shock, Csps promote tolerance to cold stress through chaperone and regulatory inputs that maintain cell membrane fluidity, enzyme activity, and gene expression. However, in departure from their name, studies have shown that some Csps are non-cold inducible suggesting that their functions are also relevant under various other non-cold stress conditions (Schmid et al., 2009; Tanaka et al., 2012; Keto-Timonen et al., 2016). In agreement with this, Csps have been shown to be important in different bacterial processes including normal growth, metabolism, virulence, and tolerance of various stress conditions such as osmotic stress, antibiotics, and nutrient starvation (Phadtare, 2004; Michaux et al., 2012; Keto-Timonen et al., 2016; Zhang et al., 2018). Similarly, characterization of *csp* gene deletion mutants in *Lm* has so far revealed that in addition to promoting cold stress, their functions are also essential for virulence, motility, biofilm formation, and resisting food-relevant stress conditions including, elevated NaCl concentrations, nisin, desiccation, and oxidative environments (Schmid et al., 2009; Loepfe et al., 2010; Schärer et al., 2013; Eshwar et al., 2017; Kragh et al., 2020; Muchaamba et al., 2021b). Our understanding of the exact molecular mechanisms through which Csps influence such a wide range of cellular processes and phenotypes is, however, currently limited. Csps are presumed to regulate transcription, translation, and mRNA stability processes in bacteria and other organisms, functions based on their interaction with DNA and RNA (Feng et al., 2001; Caballero et al., 2018; Zhang et al., 2018). As seen in other bacteria, this effect appears to be through the upregulation of effector genes either by promoting their transcription or prolonging their mRNA half-life by making it more stable (Bae et al., 2000; Phadtare, 2004; Schärer et al., 2013; Meier et al., 2017). In addition to increasing transcript levels, Csps have been shown to directly interact with transcripts promoting translation by facilitating translation initiation or through melting inhibitory mRNA secondary structures (Jiang et al., 1997; Bae et al., 2000; Phadtare et al., 2002). Such detailed knowledge on the mechanistic roles of Csps in *Lm* is however, presently lacking.

*Lm* possesses three Csps: CspA, CspB, and CspD (Glaser et al., 2001). Deletion of all three Csp encoding genes in the reference strain *Lm* EGDe showed that these proteins, though not essential for viability, are crucial in promoting the expression of stress resistance and virulence functions (Schmid et al., 2009; Loepfe et al., 2010; Schärer et al., 2013; Eshwar et al., 2017). Besides the loss of cold growth ability and increased osmotic stress sensitivity, an EGDe_*∆cpsABD* mutant was also significantly impaired in macrophage survival and virulence against zebrafish embryos, as well as secretion of the virulence factor Listeriolysin O (LLO; Schmid et al., 2009; Loepfe et al., 2010; Schärer et al., 2013; Eshwar et al., 2017). Csp deficiency also reduced cellular aggregation, flagella expression, and motility (Eshwar et al., 2017). One caveat in this knowledge is that these observations are mainly based on a single reference strain *Lm* EGDe (LII; serotype 1/2a; CC9), which represents a *Lm* genotype associated with lower clinical impact compared to strains from hypervirulent genotypes such as LI, serotype 4b, CC1, CC2, CC4, and CC6 (Maury et al., 2016; Muchaamba et al., 2022). Thus, it is important that Csp functional roles and regulatory impacts are also understood in the context of *Lm* biodiversity that includes these more clinically relevant and highly virulent *Lm* genetic backgrounds.

A broadened investigation of Csp-dependent stress response and nutrient utilization phenomes as well as gene expression regulatory impacts was undertaken in the current study, taking into consideration the existing genetic and phenotypic diversity of *Lm*. To this end, *csp* gene deletion mutants in different *Lm* genetic backgrounds were characterized in comparison to their corresponding parental wildtype (WT) strains.

## Materials and methods

### Bacterial strains, genetic manipulations, and culture conditions

The strains used in this study are listed in Supplementary Table S1. These strains were selected as LI, LII, and LIII genetic backgrounds representatives based on their genome, stress tolerance, and virulence profiles observed in our previous studies (Muchaamba et al., 2019, 2022). The *Lm* reference strain, *Lm* EGDe, was also included. Starting from frozen stocks (−80°C), strains were grown to single colonies on Brain Heart Infusion (BHI; Oxoid, United Kingdom) agar plates. For Phenotype Microarray (PM) assays, single colonies were picked from the primary plates and re-streaked on BHI agar plates generating cultures that were subsequently used for all the PM assays. To conduct optical density-based growth assays, strains were pre-cultured twice in BHI broth (16–18 h at 37°C and 150 rpm), generating secondary stationary growth phase cultures that were used for all experiments unless otherwise stated.

#### Creation of *csp* gene deletion mutants and their complemented strains

*Lm* wildtype (WT) strain representatives of LI, LII, and LIII genetic backgrounds were used to create marker-less in-frame *csp* gene deletion mutants. The *csp* gene deletion mutant series and complemented strains in *Lm* EGDe have been previously described (Schmid et al., 2009; Muchaamba et al., 2021b). Deletion of *csp* genes in the other *Lm* strains was performed through a double homologous recombination and allelic exchange process (Smith and Youngman, 1992; Camilli et al., 1993), mediated by the pKSV7-Δ*cspA* and pKSV7-Δ*cspB* plasmids as previously described (Schmid et al., 2009). Plasmid constructs used, and the *Lm* gene deletion and complementation mutants were all confirmed through DNA sequencing.

### Phenotype microarray

PM (Bochner, 2009)^1^ were used to determine metabolic and stress tolerance profiles of WT and ∆*csp* mutant strains. Phenotypic comparisons were made with respect to carbon (C-) source utilization (PM01-02), osmotic and pH (PM09-10), as well as chemical and antibiotic (PM11-20) stress sensitivity (full list of test conditions last accessed 6 September 2022).^2^ All experiments were performed at 37°C over 48 h following standard Biolog protocols with few modifications as previously described (Fox and Jordan, 2014; Muchaamba et al., 2019). A starting inoculum cell suspension with 85% transmittance was used. Using the mean of two biological replicates, output data were analyzed using opm in R and DuctApe packages as previously described (Galardini et al., 2014; Göker et al., 2016). Analysis was mainly based on area under the curve (AUC) in opm and activity index (AV) in DuctApe.

#### Optical density-based evaluation of growth

Optical density-based growth assays were used to validate selected PM observations. Growth phenotypes on L-rhamnose and D-glucose as C-sources were validated using Nutrient broth media (NB; Oxoid, United Kingdom) supplemented (1%; w/v) with the defined C-sources. Stress growth phenotypes in presence of NaCl, benzalkonium chloride (BC; Sigma-Aldrich, Buchs, Switzerland), and ß-phenylethylamine (PEA; Sigma-Aldrich Chemie GmbH, Steinheim, Germany) stress conditions were determined in BHI broth containing the different (6% NaCl; 0–8 μg/ml BC, and 6.25 mg/ml PEA) stressors. In all cases, inoculation was achieved by diluting BHI secondary cultures prepared for each strain as described above to 10^7^ CFU/ml (10^5^ for NaCl and BC assays) in the appropriate media. The cultures were prepared in triplicate for each strain (200 ul) in a 96-well microtiter plate. Plates were incubated 24 to 48 h at 25 and 37°C with shaking in a Synergy HT optical density (OD) reader (BioTek Instruments, GmbH, Switzerland), and OD was measured every 30 min at 600 nm (OD_600_). Growth analysis was also done in two 10 ml BHI cultures per strain inoculated at 10^7^ CFU/ml and incubated at 4 and 8°C for 19 days without shaking to compare growth between strains under cold and dual cold (8°C) plus NaCl (6.5%) stress. Growth in such experiments was assessed through OD_600_ measurements and CFU counts. Growth kinetics such as lag phase duration, maximal growth rate, and AUC were determined from the OD_600_ growth data using R and Graphad Prism (Göker, 2016; Göker et al., 2016). Three independent biological replicates were done for each setup.

### Antibiotic sensitivity

Bacteria solutions equivalent to 0.5 McFarland standard density were prepared from bacteria grown overnight at 37°C on blood agar plates (Columbia blood agar base, 5% sheep blood, Oxoid, Switzerland), and plated to form a lawn on Muller Hinton plus 5% sheep blood agar plates (Oxoid, Wesel, Germany). Tests for antibiotic susceptibility using antibiotic-impregnated disks and E-test strips against a panel of 28 antimicrobials (Supplementary Table S2) were done in accordance with the manufacturer’s recommendations (Biomerieux, Lyon, France). Results were assessed after incubation at 37°C for 48 h. Three independent biological replicates were done.

### Genome analysis

A total of 56 genomes (Supplementary Table S3) were annotated and compared using the Rapid Annotation Subsystem Technology (RAST) and Seed Viewer using standard settings.^3^ Whole genome and selected gene sequences (possibly linked to phenotypic differences), as well as their corresponding protein sequences, were also compared using CLC Genomics version 20.0.3. (Qiagen, Prismet, Denmark). Such analysis allowed for the determination of single nucleotide polymorphisms (SNPs), insertions and deletions (InDels), as well as the resulting amino acid changes. Where applicable, *Lm* strains EGDe and LL195 genomes were used as the references for SNP analysis. For quality control, the selected gene and corresponding protein sequences were analyzed using BLASTn and BLASTp in the National Center for Biotechnology Information (NCBI) platform.^4^

The pan-genome for the seven WT strains used in this study and 49 other genomes representing LI, LII, and LIII (Supplementary Table S3) was generated using the *Lm* BIGSdb-platform.^5^ The pan-genome was double-checked for SNP-induced false discoveries of gene absence by manual BLAST using CLC Genomics. DuctApe software was used to link PM observations and genomic data, identifying metabolic pathways and proteins that could be responsible for the phenotypic variations observed in C-source utilization (Galardini et al., 2014). Genes described in the Kyoto Encyclopedia of Genes and Genomes (KEGG) database were considered in this approach.

### RNA extraction and reverse transcription quantitative PCR analysis

The impact of PEA stress and Csp deficiency on the expression of *csp* and rhamnose utilization genes (*rhaB*, *rhaD*, and *rhaR*), respectively, were assessed by reverse transcription quantitative PCR (RT-qPCR) using primers listed in Supplementary Table S4. To determine PEA stress impact on *csp* gene expression, *Lm* WT strains were grown to stationary phase (16 h at 37°C and 150 rpm) in BHI supplemented with 0 and 6.25 mg/ml PEA. To evaluate Csp deficiency impact on rhamnose utilization genes expression, overnight secondary cultures of EGDe WT and Δ*cspABD* strains grown in 10 ml BHI, as described above, were centrifuged (3,400 g for 5 min). Bacterial pellets were washed once in PBS and resuspended in 10 ml NB with (1%; w/v) or without L-rhamnose supplementation. The cultures were incubated at 37°C. At the start (t_0_) and during incubation [rhamnose: 15 (t_15_) and 30 (t_30_) mins, and PEA: 16 h], 1 ml aliquots per sample were harvested in RNA Protect Bacteria reagent (Qiagen, Hombrechtikon, Switzerland). RNA was extracted using the RNeasy Plus Mini Kit (Qiagen GmbH, Hilden, Germany) as previously described (Eshwar et al., 2017). Yield and quality of the extracted RNA were assessed using the Quanti Fluor RNA System (Promega, Madison, United States) and the BioAnalyzer (Agilent Technologies, United States) according to the manufacturers recommendations. RNA (400 ng and RIN ≥ 8) was converted to cDNA using the Quantitect Reverse Transcription Kit (Qiagen GmbH, Hilden, Germany). Samples (2.5 ng diluted cDNA each) were amplified using the SYBR green I kit (Roche Molecular Diagnostics GmbH, Mannheim, Germany) in the Light Cycler LC480 instrument (Roche Molecular Diagnostics, Rotkreuz, Switzerland). Relative mRNA quantification was performed using the Light Cycler 480 Relative Quantification Software (Roche Molecular Diagnostics). The 16S rRNA gene was used as a reference for normalizing the mRNA amounts (Tasara and Stephan, 2007). For the rhamnose utilization operon expression assays, gene fold induction was determined relative to mRNA abundance in the control t_0_ samples. Three independent biological replicates were done.

### Statistical analyses

All experiments presented were performed independently in triplicate at least three times unless stated otherwise. GraphPad Prism [Version 9.2.0 (283), GraphPad Software, San Diego, California United States] was used for statistical analysis of data. Growth curve kinetics parameters such as AUC were extracted and analyzed using Graphad Prism and R following previously described protocols (Göker, 2016; Göker et al., 2016). PM data was analyzed using opm in R. In this analysis Tukey HSD test was used to assess the statistical significance of differences. Antibiotic sensitivity and *csp* gene expression data were analyzed using the t-test. Rhamnose utilization gene expression data, PEA stress tolerance and cold growth comparison data were assessed for statistical significance of differences using One-way ANOVA with post-hoc Tukey HSD tests. *p* values <0.05 were considered statistically significant.

## Results

### Global impact of Csps On nutrient utilization and stress tolerance in *Lm*

**Figure 1:**
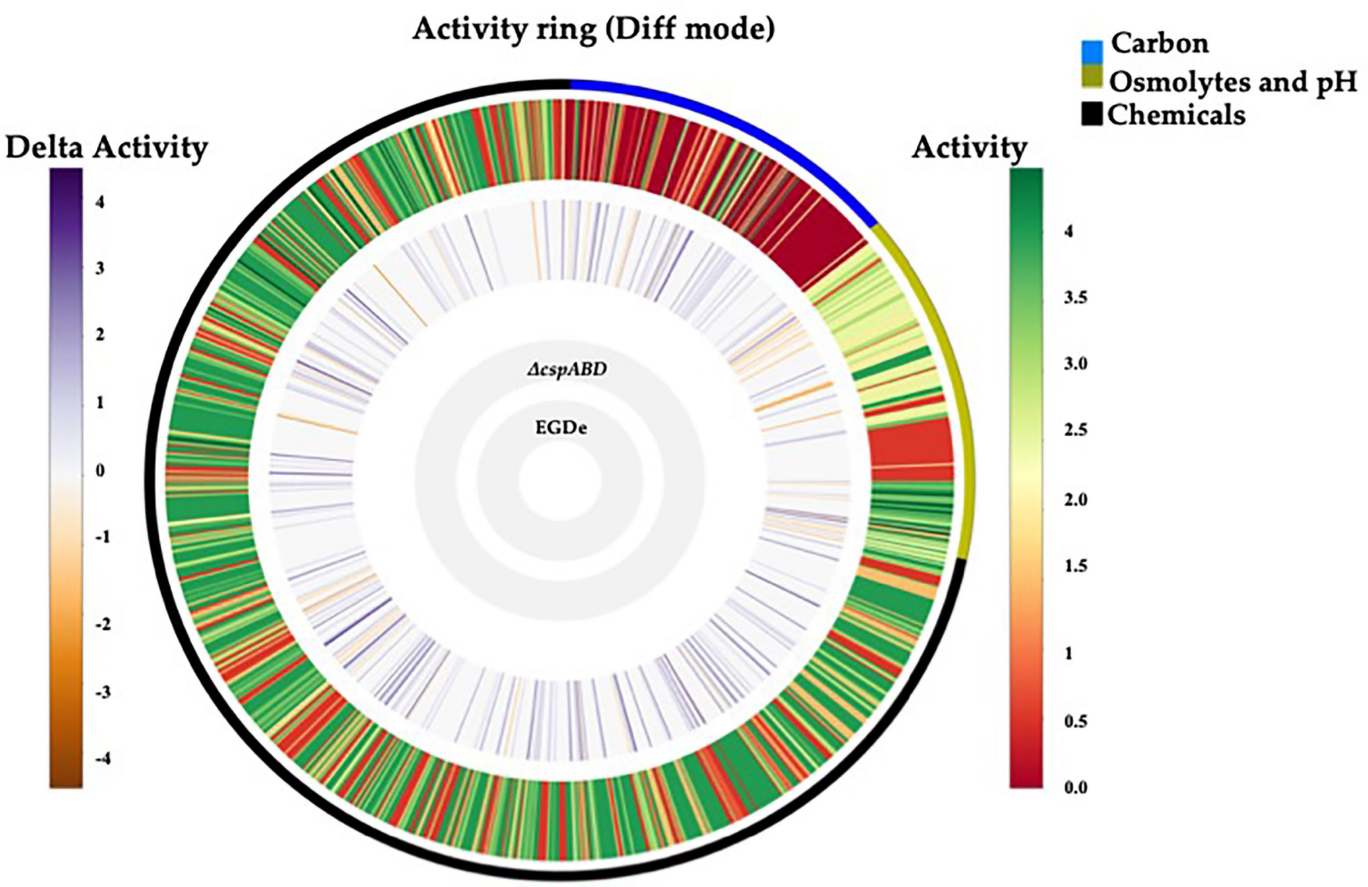
Csps functions are crucial in nutrient utilization and stress tolerance of *Lm*. Metabolic activity ring-based on PM profile comparison between *Lm* EGDe WT and ∆*cspABD* strains on PM01-02 (C-source utilization), PM09-10 (osmolytes and pH stress), and PM11-20 (chemical stress sensitivity). Gray inner circles indicate the strains’ order; external circle indicates the PM categories: e.g., black = chemical stress assays. The activity index (AV) calculated for each strain and well is reported as color stripes going from red (AV = 0) to green (AV = 4). Delta activity: the difference between the AV values of the WT strain (EGDe) and the ∆*cspABD* mutant is reported when equal to or higher than 2 AV units; gray: indicates no significant difference; purple: higher WT activity; orange: lower WT activity.

**Figure 2:**
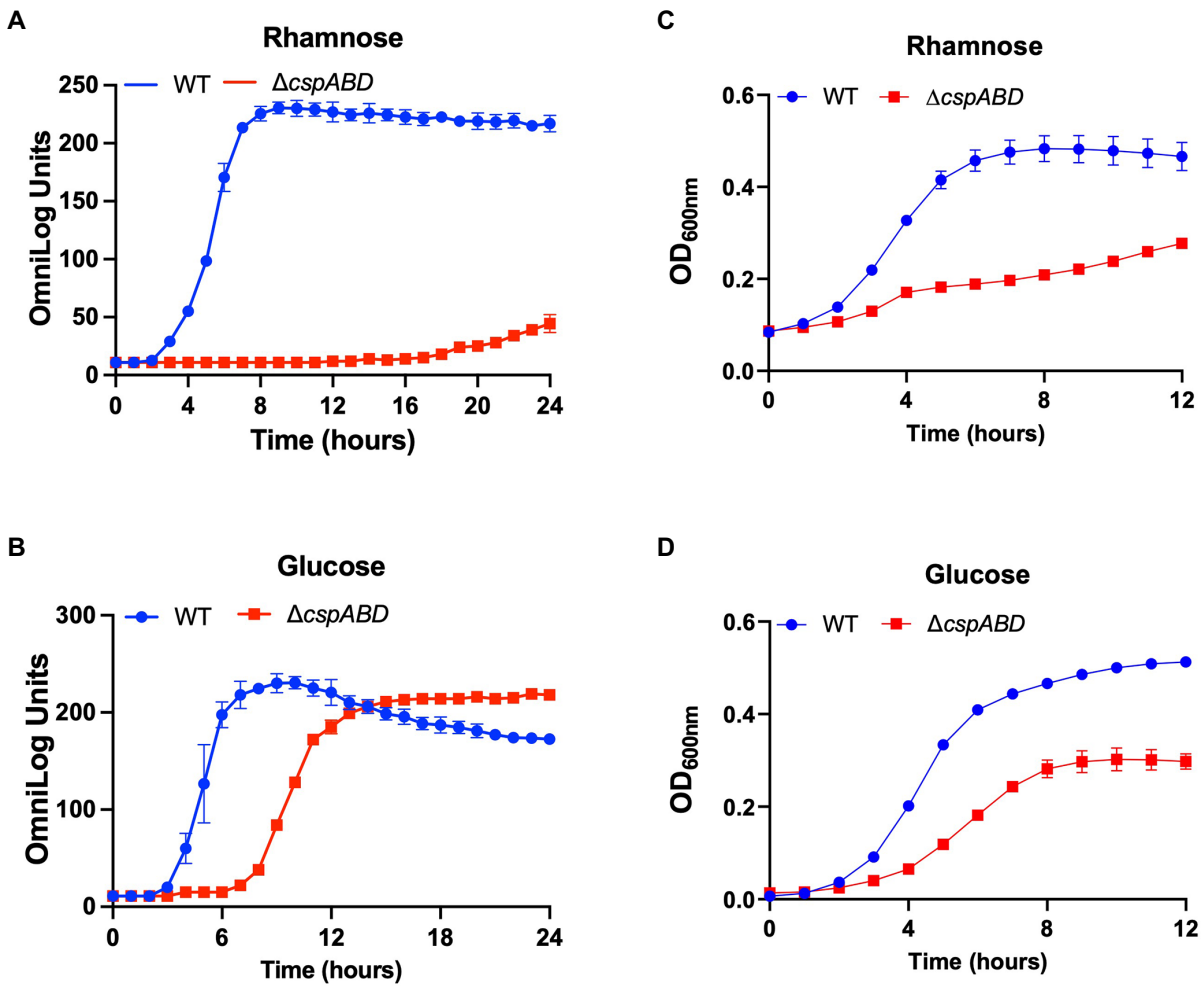
Csp deficiency severely impairs rhamnose and glucose utilisation efficiency in *Lm*. PM-based growth curves of *Lm* EGDe WT and Δ*cspABD* strains grown at 37°C with L-rhamnose **(A)**, or D-glucose **(B)**, as the sole C-sources. PM observations were corroborated using OD-based growth phenotypic assays. Strains were cultivated in NB supplemented (1% w/v) with L-rhamnose **(C)**, or D-glucose **(D)**. Presented data are means and their corresponding standard deviation (error bars) from two (PM) and three (OD-based) independent biological replicates.

**Figure 3:**
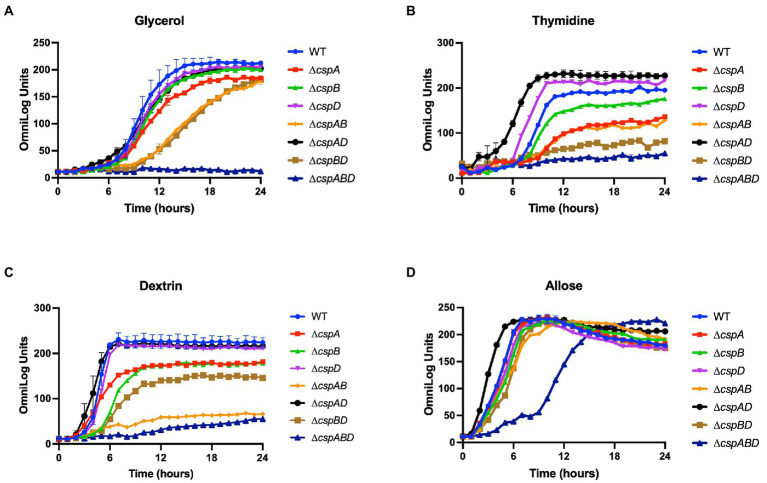
Variable contribution of the individual Csps to C-source utilization in *Lm* EGDe. Presented data are means and their corresponding standard deviation (error bars) from two biological replicates of PM derived metabolic activity curves generated at 37°C using **(A)** glycerol, **(B)** thymidine, **(C)** dextrin, and **(D)** allose as sole C-sources.

Initially, a comprehensive overview of the impact of Csps on nutrient utilization and stress tolerance in *Lm* was generated using the *Lm* reference strain EGDe. PM comparison of EGDe WT strain and its Δ*cspABD* mutant, deleted for all the three *csp* genes, showed that Csp functions are crucial for the optimal utilization of several C-sources and tolerance to various stress conditions. Growth/metabolic activity of the EGDe_Δ*cspABD* mutant compared to the WT strain was lost on 10 compounds and utilization capacity significantly reduced (*p* < 0.05) on another 25 of the 190 C-sources examined (Figures 1–3; Table 1; Supplementary Figures S1A, S2A; Tables S5, S6). Notably, Csp loss impaired *Lm*’s ability to utilize several C-sources relevant for survival and growth within host and food-associated environments such as glucose (2-fold), glycerol (9-fold), palatinose (3.7-fold), L-rhamnose (11-fold), β-D-allose (2.1-fold), N-acetyl-D-glucosamine (2-fold), and dextrin (6-fold). The ∆*cspABD* mutation also increased *Lm* EGDe sensitivity to osmotic, pH, and various chemical stressors (Figure 1; Supplementary Tables S7–S9). A selection of the PM observations was further corroborated *via* OD-based growth assays confirming that growth of the ∆*cspABD* mutant on D-glucose and L-rhamnose was impaired compared to the EGDe WT strain (Figure 2). Overall, the analysis of the EGDe_Δ*cspABD* mutant thus provides an extensive overview of Csp functional contributions to C-source utilization and stress response in *Lm*.

**Table 1:** Impact of different *csp* mutations on C-source utilization capacity of *Lm* EGDe strain.

**PM01**	**EGDe**	**∆*cspA***	**∆*cspB***	**∆*cspD***	**∆*cspAB***	**∆*cspAD***	**∆*cspBD***	**∆*cspABD***
D-Galactose	**−**	**−**	**−**	**−**	**−**	**+**	**−**	**−**
D-Sorbitol	**−**	**−**	**−**	**−**	**−**	**+**	**−**	**−**
Glycerol	**+**	**+**	**+**	**+**	**+**	**+**	**+**	**−**
L-Fucose	**−**	**−**	**−**	**−**	**−**	**+**	**−**	**−**
D-Glucose-6-Phosphate	**+** ^**a**^	**−**	**−**	**−**	**−**	**−**	**+**	**+** ^**a**^
L-Rhamnose	**+**	**+**	**+**	**+**	**+**	**+**	**+**	**−**
Thymidine	**+**	**+**	**+**	**+**	**+**	**+**	**−**	**−**
α-Keto-Butyric Acid	**+**	**−**	**+**	**+**	**−**	**+**	**−**	**−**
α-D-Lactose	**−**	**+**	**−**	**−**	**−**	**+**	**−**	**−**
α-D-Glucose-1-Phosphate	**+** ^**a**^	**−**	**−**	**−**	**−**	**−**	**−**	**+** ^**a**^
D-Fructose-6-Phosphate	**−**	**−**	**−**	**−**	**−**	**+**	**−**	**+** ^**a**^
2′-Deoxy-Adenosine	**−**	**−**	**−**	**−**	**−**	**+**	**−**	**−**
Adenosine	**+**	**−**	**−**	**−**	**−**	**+**	**−**	**−**
D-Cellobiose	**+**	**+**	**+**	**+**	**−**	**+**	**+**	**+**
*N*-Acetyl-β-D-Mannosamine	**+**	**+**	**+**	**+**	**+**	**+**	**+**	**−**
Methyl Pyruvate	**−**	**−**	**−**	**−**	**−**	**+**	**−**	**−**
D-Psicose	**+**	**+**	**+**	**+**	**+**	**+**	**+**	**−**
Glucuronamide	**−**	**−**	**−**	**−**	**−**	**+**	**−**	**−**
Pyruvic Acid	**−**	**−**	**−**	**−**	**−**	**+**	**−**	**−**
**PM02**								
Dextrin	**+**	**+**	**+**	**+**	**−**	**+**	**+**	**−**
Glycogen	**−**	**−**	**−**	**−**	**−**	**+**	**−**	**−**
D-Arabinose	**−**	**−**	**−**	**−**	**−**	**+**	**−**	**−**
2-Deoxy-D-Ribose	**+**	**+**	**+**	**+**	**+**	**+**	**−**	**+**
α-Methyl-D-Glucoside	**+**	**+**	**+**	**+**	**−**	**+**	**+**	**−**
3-*O*-Methyl-D-Glucose	**−**	**−**	**−**	**−**	**−**	**+**	**−**	**−**
Palatinose	**+**	**+**	**+**	**+**	**−**	**+**	**+**	**−**
D-Tagatose	**−**	**−**	**−**	**−**	**−**	**+**	**−**	**−**
Turanose	**−**	**−**	**−**	**−**	**−**	**+**	**−**	**−**
α-Keto-Valeric Acid	**−**	**−**	**−**	**−**	**−**	**+**	**−**	**−**
Oxalomalic Acid	**−**	**−**	**−**	**−**	**−**	**+**	**−**	**−**
2,3-Butanedione	**−**	**−**	**−**	**−**	**−**	**+**	**−**	**−**

PM derived table generated using opm based AUC comparison. Key: The symbols and colors indicate: **–**, not able to metabolize; **+**, able to metabolize the C-source.

a Weak metabolic activity observed.

### Contribution of the individual Csps to C-source utilization in *Lm* EGDe

We next assessed the functional contributions of the individual Csps towards the PM uncovered phenotypes of the *Lm* EGDe_Δ*cspABD* mutant. To this end, single (Δ*cspA*, Δ*cspB,* and Δ*cspD*), double (Δ*cspAB*, Δ*cspAD*, and Δ*cspBD*), and triple (Δ*cspABD*) *csp* gene deletion mutants in *Lm* EGDe were included in PM analysis. This revealed several differences in C-source utilization and stress resilience between the EGDe WT strain and different *csp* gene deletion mutants (Table 1; Supplementary Figures S1A, S2A). *Lm*’s ability to utilize 26 C-sources, including those relevant in infected host cells and food environments, was found to differ between the EGDe WT and its *csp* mutant strains. The phenotypic changes revealed sometimes varied depending on the type of *csp* deletion mutation. A ∆*cspABD* mutation eliminating all three *csp* genes, for example, severely attenuated glycerol, thymidine, dextrin, and allose utilization, while the ability to utilize these C-sources was enhanced or unaltered in a ∆*cspAD* double deletion mutation, which removed *cspA* and *cspD* leaving the *cspB* gene (Figures 3A–D). The phenotypes that emerged in utilizing glycerol, an important intracellular C-source, were consistent with functional redundancy and epistatic interactions between the *csp* genes (Figure 3A). Although Δ*cspABD* mutation removing all three *csp* genes almost completely attenuates metabolic activity on glycerol, no phenotypic defects were observed for ∆*cspB*, ∆*cspD*, and ∆*cspAD* mutants consistent with redundancy in the functional contributions of Csps to glycerol utilization. Relative to the WT strain, deletion of *cspB* could only induce a significant reduction (*p* < 0.05) in glycerol utilization when combined with either *cspA* (∆*cspAB*: 1.6-fold) or *cspD* (∆*cspBD*: 1.7-fold; Figure 3A) deletion. Thus, although *cspA* overall appears to be the main *csp* gene involved in glycerol utilization, its functions seem epistatic with *cspB*. An overall hierarchical trend of CspB>CspA>CspD was observed with respect to their roles in thymidine, dextrin, and palatinose utilization as C-sources (Figures 3B–C; Table 1; Supplementary Figure S2A). Deletions of *cspA* (∆*cspA*: 1.65-fold), *cspB* (∆*cspB*, ∆*cspAB,* and ∆*cspBD*: 1.2-, 1.73-, and 2.3-fold, respectively) or all three *csp* (∆*cspABD*: 3.4-fold) genes reduced thymidine utilization. On the other hand, the deletion of *cspD* (∆*cspD* and ∆*cspAD,* 1.2- and 1.38-fold, respectively), had minimum effects on thymidine utilization (Figure 3B). Dextrin utilization was significantly reduced upon *cspA* (∆*cspA*, ∆*cspAB*, and ∆*cspABD*: 1.25-, 3.7-, and 6-fold, respectively) or *cspB* deletion (∆*cspB* and ∆c*spBD*: 1.4- and 1.7-fold, respectively), while *cspD* deletion had no impact (Figure 3C).

A hierarchical trend of CspD>CspB>CspA was observed with respect to Csp roles in rhamnose utilization as a sole C-source by *Lm* EGDe. Utilization was more significantly attenuated (p < 0.05) in ∆*cspD*, ∆*cspBD*, and ∆*cspABD* mutants (2-, 3-, and 11-fold, respectively), whereas *cspA* deleted alone (Δ*cspA*) had no impact compared to the WT strain (Supplementary Figure S1A). Further epistatic and hierarchical trends were observed in the utilization of other C-sources by the different *Lm* EGDe *csp* gene deletion mutants (Supplementary Figures S1A, S2A; Table S10).

Interestingly, *cspB* expressed alone without *cspA* and *cspD* (∆*cspAD* mutant) caused a gain of function enabling utilization of 18 extra C-sources compared to the WT (Table 1; Supplementary Figures S1A, S2A). The ability of *Lm* EGDe to utilize phosphate sugars, another important intracellular C-source, was affected by Csp deficiency. For example, D-glucose-6-phosphate utilization was enhanced by ∆*cspBD* and ∆*cspABD* mutations compared to the WT strain (Table 1; Supplementary Figure S1A). These improved or gain of function phenotypes in the absence of other *csp* genes might be suggestive of regulatory influences between *csp* genes. Csps might also have inhibitory regulatory roles on some C-source utilization systems. Overall, our observations have uncovered varying roles, epistatic interactions, and functional redundances between individual *csp* genes with respect to the regulation of utilization pathways for several C-sources.

#### Strain and genetic background-associated variations in Csp contributions to C-source utilization

**Figure 4:**
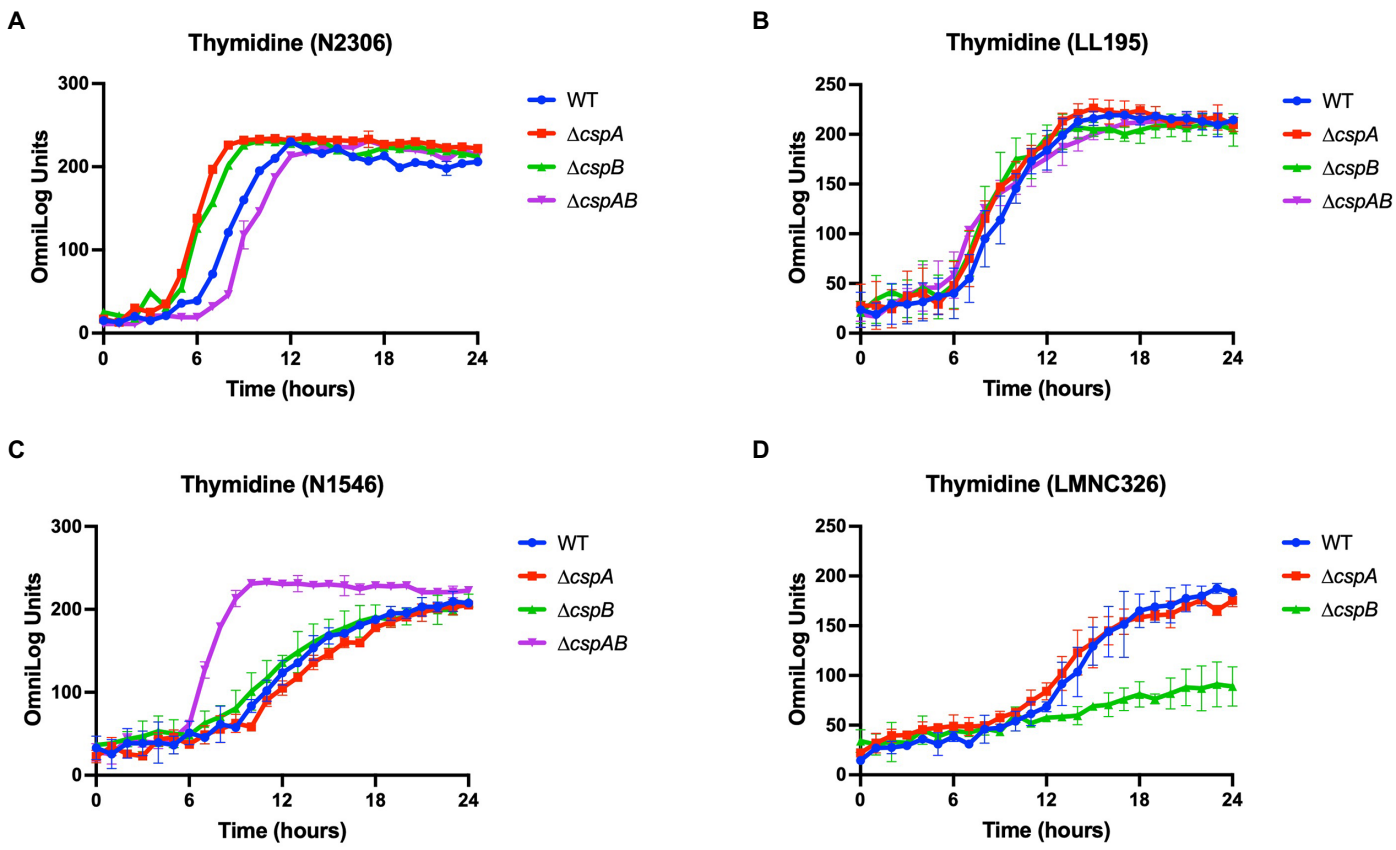
Variable effects of *csp* deletion mutations on thymidine utilization observed in **(A)** *Lm* N2306 (LI; CC4), **(B)** LL195 (LI; CC1), **(C)** N1546 (LII; CC8), and **(D)** LMN328 (LIII; CC70) genetic backgrounds. The data presented are means and their corresponding standard deviation (error bars) from two biological replicates of PM derived metabolic activity curves at 37°C with thymidine as the sole C-source.

Additional to EGDe, Csps involvement in C-source utilization within other *Lm* strains and genetic backgrounds was examined. Deleting *cspA* and *cspB* genes in the genetic backgrounds of *Lm* N1546 (LII, CC8), LL195 (LI, CC1), N2306 (LI, CC4), N16-0044 (LI, CC6), LMNC318 (LIII, CC70), and LMNC328 (LIII, CC70) strains significantly (p < 0.05) affected utilization of 10, 12, 21, 11, 5, and 8 C-sources, respectively (Table 2; Supplementary Tables S5–S6; Figures S1B–G, S2B–G). Observed effects of *csp* deletion on C-source utilization displayed genetic background-associated differences in some cases (Figure 4). The deletion of *cspA* and *cspB* genes had minimum effects in LI strains (N2306, LL195, and N16-0044) on thymidine utilization, but there were more significant effects in LII (EGDe and N1546) and LIII (LMNC318 and LMNC326) strains (Figure 4; Supplementary Figure S1). Although ∆*cspA* significantly reduced glycerol utilization in LIII strains, such a mutation had no detectable impact in the tested LI and LII strains. A ∆*cspAB* mutation in N1546 (LII) and N2306 (LI) backgrounds reduced lactose utilization, while in other *Lm* strains, it had no significant influence. Removal of *cspA* and *cspB* reduced g-cyclodextrin utilization in all three LI strains (N2306, LL195, and N16-0044), while LII (N1546) and LIII (LMNC318 and LMNC326) strains were not affected (Supplementary Figure S2B–G). Similar to *Lm* EGDe (LII; CC9), L-rhamnose utilization was significantly reduced without *cspA* and *cspB* in N2306 (LI; CC4) and LL195 (LI; CC1), while allose utilization was decreased in N1546 (LII; CC8; Table 2). On the other hand, glycerol utilization was altered in ∆*cspAB* mutants of strains from all three lineages (Table 2).

**Table 2:** Impact of *csp* deletion mutations on carbon source utilization in LI, LII, and LIII representative strains.

**PM condition** ^**a**^	**LII**				**LI**												**LIII**					
		**N1546**			**N2306**					**LL195**				**N16-0044**			**LMNC318**			**LMNC326**		
	**WT**	∆***A***	∆***B***	∆***AB***	**WT**	∆***A***	∆***B***	∆***AB***	**WT**	∆***A***	∆***B***	∆***AB***	**WT**	∆***A***	∆***B***	∆***AB***	**WT**	∆***A***	∆***B***	**WT**	∆***A***	∆***B***
Glycerol	**+**	**+**	**+**	**+**	**+**	**+**	**+**	**+**	**+**	**+**	**+**	**+**	**+**	**+**	**+**	**+**	**+**	**+**	**+**	**+**	**+**	**+**
L-Rhamnose	**+**	**+**	**+**	**+**	**+**	**+**	**+**	**+**	**+**	**+**	**+**	**+**	**+**	**+**	**+**	**+**	**+**	**+**	**+**	**+**	**+**	**+**
β-D-Allose	**+**	**+**	**+**	**+**	**−**	**−**	**−**	**−**	**−**	**−**	**−**	**−**	**−**	**−**	**−**	**−**	**−**	**−**	**−**	**−**	**−**	**−**
Dextrin	**+**	**+**	**+**	**+**	**+**	**+**	**+**	**+**	**+**	**+**	**+**	**+**	**+**	**+**	**+**	**+**	**+**	**+**	**+**		**+**	**+**
α-D-Lactose	**+**	**+**	**+**	**+**	**+**	**+**	**+**	**+**	**−**	**−**	**−**	**−**	**−**	**−**	**−**	**−**	**−**	**−**	**−**	**−**	**−**	**−**
Inosine	**+**	**+**	**+**	**+**	**+**	**+**	**+**	**+**	**+**	**+**	**+**	**+**	**+**	**+**	**+**	**+**	**+**	**+**	**+**	**+**	**+**	**+**
D-Tagatose	**−**	**−**	**−**	**−**	**−**	**−**	**−**	**+**	**−**	**−**	**−**	**−**	**−**	**−**	**−**	**−**	**+**	**+**	**+**	**+**	**+**	**+**
Pectin	**−**	**−**	**−**	**−**	**−**	**−**	**−**	**−**	**−**	**−**	**−**	**+**	**−**	**−**	**+**	**+**	**−**	**−**	**−**	**−**	**−**	**−**
2′-Deoxy-adenosine	**−**	**−**	**−**	**+**	**−**	**−**	**−**	**−**	**+**	**+**	**−**	**+**	**+**	**+**	**+**	**−**	**−**	**−**	**−**	**−**	**−**	**−**
pH5		**+**	**+**	**+**	**+**	**+**	**+**	**+**		**+**	**+**	**+**		**+**	**+**	**+**		**+**	**+**		**+**	**+**
NaCl	**+**	**+**	**−**	**+**	**+**			**−**	**+**	**+**	**+**	**−**	**+**	**+**	**+**	**+**	**+**	**−**	**+**	**+**	**+**	**−**
Na^+^ Formate	**−**	**+**	**−**	**+**	**−**			**+**	**+**	**+**	**+**	**−**	**+**	**+**	**+**	**+**	**+**	**+**	**+**	**+**	**+**	**+**
Na^+^ Nitrite	**−**	**−**	**−**	**−**	**−**			**−**	**−**	**−**	**+**	**+**		**+**	**+**	**+**	**−**	**−**	**+**	**−**	**−**	**+**

a PM derived table generated using opm based AUC comparison. The symbols indicate: **–**, no metabolic activity; **+**, active metabolism while colors represent; no difference (white), significantly higher (green), and lower metabolic activity (red), compared to the mutant’s respective WT strain identified using the Tukey HSD test (*p* < 0.05). Key: WT wildtype strain, ∆A: ∆*cspA*, ∆B: ∆*cspB*, and ∆AB: ∆*cspAB*.

Comparable to *Lm* EGDe, epistatic interactions, functional redundancies, and hierarchical trends were similarly observed among the different strains tested. For example, in N2306 (LI; CC4), *cspB* (∆*cspB*) deletion alone did not affect lactose utilization, whereas *cspA* (∆*cspA*) deletion increased utilization. The deletion of both genes (N2306 ∆*cspAB*), however, resulted in reduced α-D-lactose utilization capacity (Table 2). The ability of the study strains to utilize some C-sources, such as allose and lactose, varied among the WT strains, precluding comparison of Csps roles in the utilization of these C-sources across the different genetic backgrounds.

### The influence of Csps on stress resistance and chromogenic substrate catabolism in *Lm* EGDe

PM analysis showed that removing *csp* genes significantly changes *Lm* EGDe sensitivity to several osmotic stressors including NaCl, potassium chloride, urea, sodium formate, sodium benzoate, sodium nitrate, and sodium nitrite (Supplementary Table S7; Figure S3A). The phenotypes obtained were overall consistent with varying functional roles and epistatic interactions among the *csp* genes in their regulation of osmotic and acidic- or alkaline-stress tolerance. NaCl-stress tolerance was reduced upon deletion of any of the three *csp* genes with an overall hierarchical trend of *cspD* > *cspB* > *cspA* in *csp* gene importance observed (Supplementary Table S7; Figure S3A). Additionally, a significant impact of *csp* genes was observed on the capacity of *Lm* EGDe to exploit different compounds for NaCl stress tolerance. The EGDe WT strain was better at utilizing substances such as betaine, carnitine, glycerol, and trehalose for NaCl stress protection than its *csp* mutants (Supplementary Figure S3A). The most significant variability was observed in trehalose utilization under NaCl (6%) stress were a growth trend of WT>∆ *cspB* >∆*cspA*>∆*cspD* > ∆*cspBD* > ∆*cspAD* >∆*cspAB* > ∆*cspABD* was observed. These osmotic stress tolerance phenotypes indicate both functional redundancy and regulatory interactions between individual *csp* genes. Eliminating single *csp* genes, for example, increased sodium benzoate osmotic stress tolerance (∆*cspA* > ∆*cspD* > ∆*cspB*). On the other hand, deleting more than one *csp.,* as in Δ*cspAD* and Δ*cspAB* strains, reduced tolerance except for the ∆*cspBD* mutation, which increased tolerance compared to the WT (Supplementary Figure S3A). Deleting *cspD* (∆*cspD*) alone increased sodium nitrite stress tolerance while deletion of other *csp* genes, in single (∆*cspA* and ∆*cspB*) or double (∆*cspAB*, ∆*cspAD*, and ∆*cspBD*), and ∆*cspABD* deletion mutants, increased sodium nitrite stress sensitivity (Supplementary Figure S3A).

**Figure 5:**
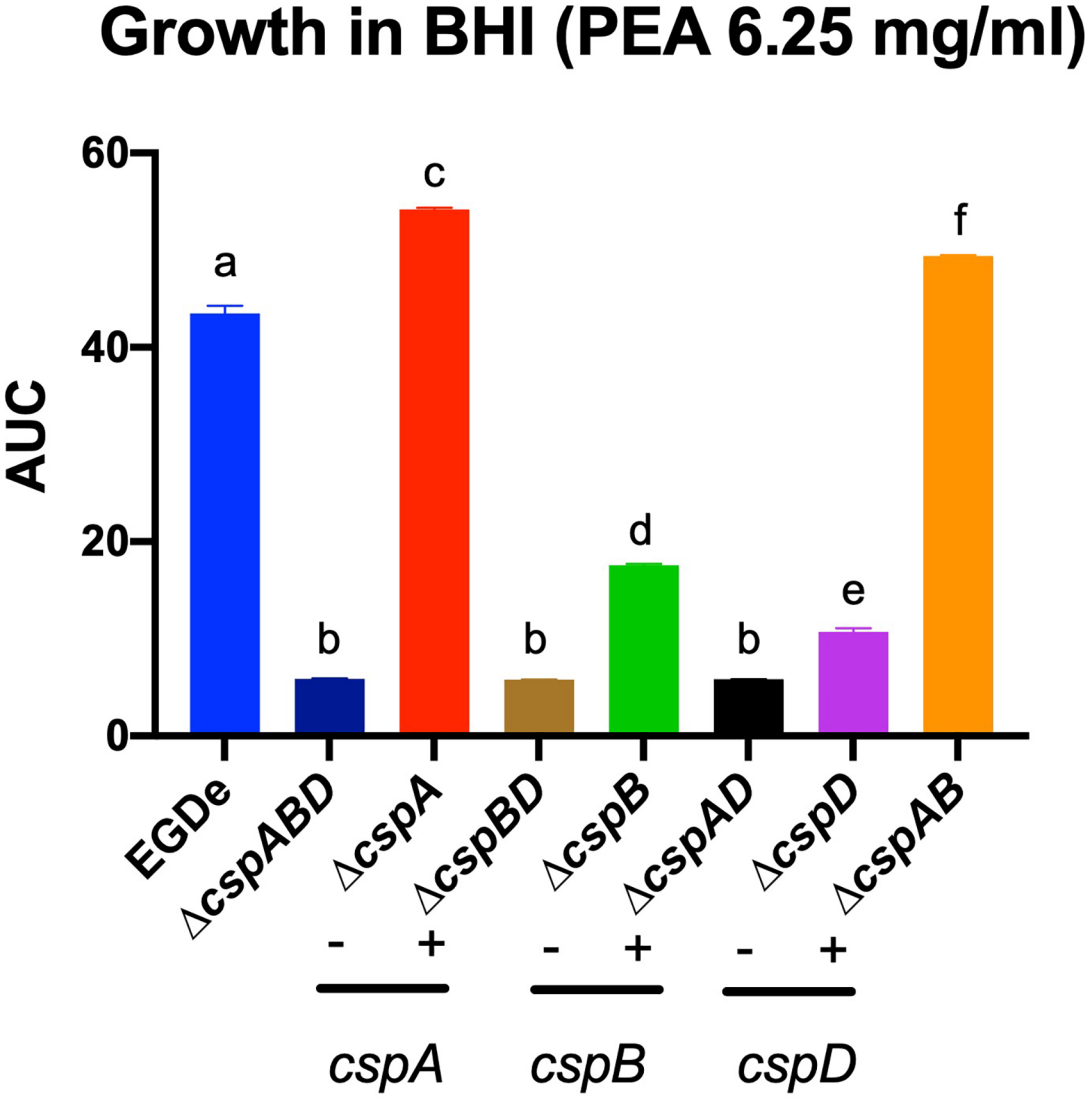
Metabolic activity profiles observed for the EGDe *csp* mutants on PM under PEA stress could be corroborated using OD-based growth phenotypic assays. Area under the curve (AUC) bar graphs derived from OD-based growth curves generated from EGDe WT and its *csp* mutants cultivated in BHI supplemented with PEA 6.25 mg/ml at 37°C. Means and their corresponding standard deviation (error bars) from three independent biological replicates conducted in triplicate for each strain are presented. No significant differences in growth parameters were observed from the strains in BHI with no stress hence the AUC data presented are directly comparable. Different letters denote significant differences between strains, detected using Tukey’s post-hoc test pairwise comparison following one-way ANOVA (*p* < 0.05).

In the case of acid stress conditions (pH 5), we found that while all *csp* deletion mutants were more sensitive, the ∆*cspA* mutant was more tolerant than the EGDe WT strain. On the other hand, at pH 6–10, all the EGDe *csp* mutants were more sensitive than the WT strain (Supplementary Figure S4A). Under alkaline stress conditions at pH 9.5, the inclusion of PEA, cadaverine, putrescine, and tyramine caused growth/metabolic activity inhibition in all EGDe strains. PEA phenotypic impacts uncovered on PMs were further corroborated using OD-based growth assays that showed functional redundancy and epistatic interactions among the *csp* genes in regulating *Lm* EGDe PEA stress response (Figure 5). An overall hierarchical trend of *cspD* > *cspB* > *cspA* was observed regarding the individual Csp contributions to PEA tolerance. A ∆*cspABD* increased PEA sensitivity. Amongst single *csp* gene deletion mutants, a Δ*cspA* mutation increased PEA resistance, while a ∆*cspD* mutation induced the most PEA sensitive phenotype. Analyzing double deletion mutants carrying single *csp* genes revealed that a ∆*cspAB* mutation retaining only *cspD* increased PEA stress tolerance to levels higher than all the other double *csp* mutants and the WT strain (Figure 5).

Deletion of *csp* genes also caused a reduction in the rate of utilization of chromogenic substrates used in the development of selective chromogenic media such as ALOA agar. The most significant variability emerged in the catabolism of 5-bromo-4-chloro-3-indolyl caprylate and 5-bromo-4-chloro-3-indolyl α-D-glucoside, where EGDe ∆*cspAB* and ∆*cspABD* mutants were the most affected. Such phenotypes were further validated when strains were grown on ALOA agar plates with these two mutants being slower in developing the characteristic blue-green color (data not shown). Csps might thus contribute to the regulation of expression of metabolic pathways or proteins such as β-glucosidases involved in utilization of such chromogenic substances. A notion supported by the observation that utilization of D-salicin, an alcoholic β-glucoside, was also reduced in the EGDe_∆*cspABD* mutant (Supplementary Figure S2A).

#### The contributions of Csps to pH and osmotic stress tolerance vary with *Lm* genetic background

The contributions of Csps to pH and osmotic stress tolerance were further examined using strains of other *Lm* genetic backgrounds in addition to the EGDe reference strain (Supplementary Table S1). Significant alterations in pH and osmotic stress tolerance, including in some cases, strain specific differences, were observed from analysis of such *csp* gene deletion mutants (Table 2; Supplementary Tables S7, S8, Figures S3B–G, S4B–G). Although several stress sensitivity profile alterations were detected among the examined strains upon loss of *csp* genes, only a few specific examples are discussed here. The ∆*cspAB* mutants of LI strains N2306, LL195, and N16-0044 were impaired under NaCl and urea stress conditions, but a similar mutation in N1546, a LII strain, increased tolerance of such stress conditions, as also seen in *Lm* EGDe (Table 2). Moreover, a ∆*cspAB* mutation reduced sodium benzoate stress tolerance in N2306 and N16-0044 (both LI) strains but not in LL195 (LI) and N1546 (LII) strains. Sodium benzoate tolerance was increased for single *cspA* or *cspB* deletion mutants (∆*cspB* > ∆*cspA* > WT) of LIII (LMNC318 and LMNC326) strains, but there was no impact observed in LI (N2306, LL195, and N16-0044) single *csp* gene deletion mutants (Supplementary Table S7; Figures S3B–G). N2306 strain ∆*cspAB* mutant had the most sensitive osmotic stress phenotype among the ∆*cspAB* mutants tested (Supplementary Table S7; Figures S3B–G). Strain and genetic background-specific trends were observed in the sensitivity of different *csp* mutants against the food preservative sodium formate (E-number: 237). A ∆*cspAB* mutation increased sodium formate tolerance for N2306 (LI) and N1546 (LII) strains while reducing it in *Lm* EGDe (LII), LL195 (LI), and N16-0044 (LI) strains. A ∆*cspB* mutation while increasing NaCl stress sensitivity in N16-0044 (LI), N1546 (LII), and LMNC326 (LIII) strains caused an opposite effect in LL195 (LI) and LMNC318 (LIII) strains, where it increased tolerance (Table 2). ∆*cspA* mutations in LIII strains LMNC318 and LMNC326 increased NaCl sensitivity, while in *Lm* N1546, a LII strain, such a mutation decreased sensitivity, and there were no phenotypic impacts observed for the LI strains LL195 and N16-0044 (Table 2). Among the *csp* mutants examined, we found that the LL195_∆*cspAB* mutant benefited the most when considering protective effects induced through osmo-protectants such as betaine and carnitine. On the other hand, a N2306_∆*cspAB* mutant became more sensitive to NaCl stress upon the inclusion of potassium chloride or carnitine (Supplementary Table S7; Figures S3B–G).

A *cspA* and *cspB* double deletion mutation (∆*cspAB*) increased acid stress sensitivity in *Lm* N2306, N1546, LL195, and N16-0044 strains, similar to observations with EGDe (Supplementary Table S8; Figures S4B–G). In contrast, ∆*cspA* and ∆*cspB* mutations in N1546 strain reduced (4.3- and 2.4-fold, respectively) acid stress sensitivity. Meanwhile, tolerance of alkaline stress was increased upon inclusion of L-proline and L-tyrosine in all strains and *csp* deletion mutants (Supplementary Table S8; Figures S4A–G). On the other hand, the addition of cadaverine, putrescine, PEA, and tyramine decreased tolerance. Compared to the WT and ∆*cspAB*, tyramine inclusion increased alkaline stress (pH 9.5) sensitivity in N2306 ∆*cspA* and ∆*cspB* mutants. N2306 ∆*cspA* and ∆*cspB* on the other hand, showed better PEA tolerance than the WT and ∆*cspAB* strains. In LL195, LMNC318, and LMNC326, all the *csp* mutants were more sensitive to alkaline stress in the presence of PEA and tyramine compared to the WT. As observed in *Lm* EGDe, a ∆*cspA* mutation in *Lm* N16-0044 also increased PEA tolerance. The inclusion of substances such as glycine, agmatine, L-asparagine, L − threonine, or L-serine at pH 9.5 lowered N2306_∆*cspAB* and LL195_∆*cspAB* tolerance to alkaline stress (Supplementary Table S8; Figures S4A–G). PM-derived data on PEA stress sensitivity was corroborated in BHI at neutral pH using an OD-based approach, revealing similar strain-specific inhibition trends (Supplementary Figure S5).

### The impacts of Csp loss on chemical stress resistance profiles in *Lm* EGDe

**Figure 6:**
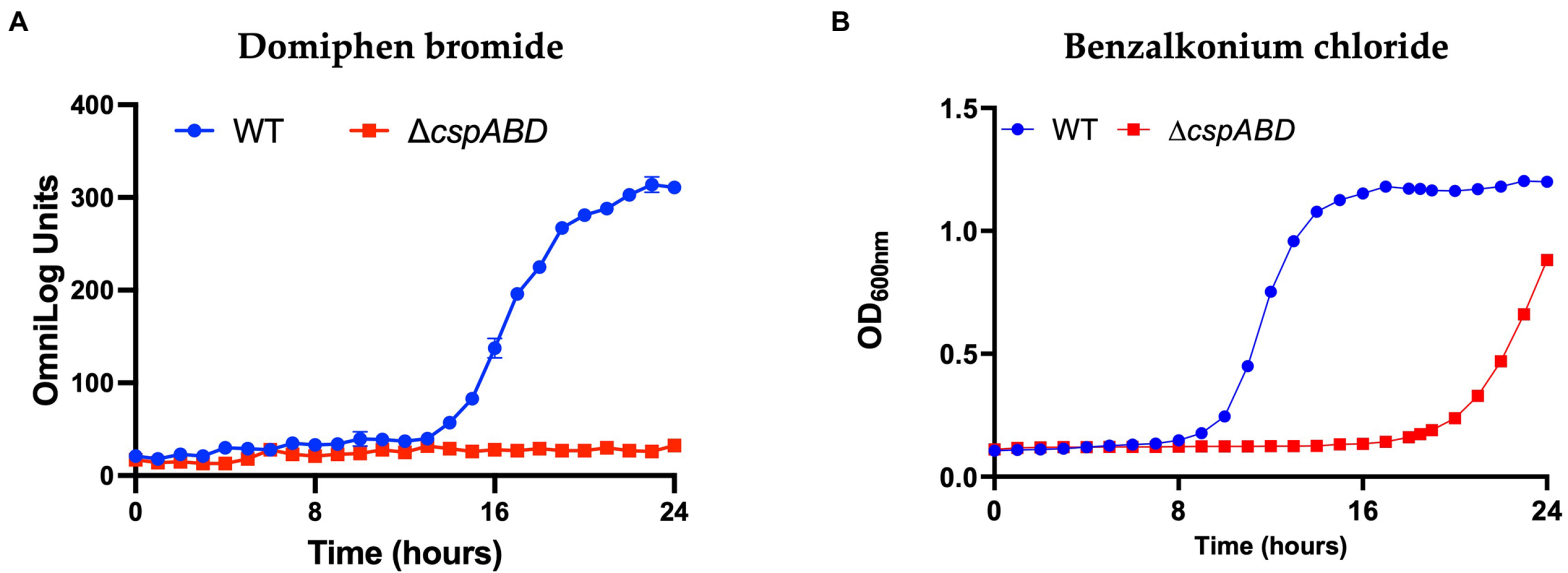
Csp loss increases *Lm* sensitivity to quaternary ammonium compounds. Presented data are means and their corresponding standard deviation (error bars) from **(A)**, PM generated metabolic activity curves and **(B)**, OD-based growth curves generated at 37°C in the presence of **(A)**, domiphen bromide and **(B)**, benzalkonium chloride (BHI) from two and three independent biological replicates, respectively.

The Δ*cspABD* mutation in *Lm* EGDe increased sensitivity to 56 chemicals that included different classes of antibiotics (Table 3; Supplementary Table S9), while sensitivity to 5 other chemical substances, including ketoprofen, was reduced. An overall trend emerged showing that Csp loss increased sensitivity to antibiotics targeting metabolic pathways (e.g., trimethoprim), the cell wall (e.g., carbenicillin), the cell membrane (e.g., domiphen bromide), and 30S ribosomal subunit (e.g., neomycin; Table 3; Supplementary Table S9). A selection of these PM-based observations was corroborated using OD-based growth curves and disk or E test based antimicrobial sensitivity profile comparisons (Table 4; Figure 6; Supplementary Table S2). Based on this, Csps seem relevant in the regulation of protein synthesis and metabolic pathways related to general cell, cell membrane, and cell wall homeostasis.

**Table 3:** Impact of Csp loss on *Lm* EGDe chemical stress tolerance.

PM09 to PM20^a^	EGDe	∆*cspABD*	PM09 to PM20^a^	EGDe	∆*cspABD*
9% NaCl	**+**	**−**	Iodoacetic Acid	**−**	**+**
200 mM Sodium Benzoate + pH 5.2	**+**	**−**	Ketoprofen	**−**	**+**
β-Phenylethylamine + pH 9.5	**+**	**−**	3,4-Dimethoxybenzyl Alcohol	**−**	**+**
Cloxacillin	**+**	**−**	Azlocillin	**−**	**+**
Cephalothin	**+**	**−**	Sodium Bromate	**−**	**+**
Carbenicillin	**+**	**−**	Cadmium Chloride	**+**	**−**
Sulfamethoxazole	**+**	**−**	Cesium Chloride	**+**	**−**
Trimethoprim	**+**	**−**	Guanidine Hydrochloride	**+**	**−**
Tylosin	**−**	**−**	Copper [II] Chloride	**+**	**−**
Neomycin	**+**	**−**	Sodium Arsenate	**+**	**−**
Domiphen Bromide	**+**	**−**	Sodium Dichromate	**+**	**−**

a Key: –, negative reaction, strain not metabolically active; +, positive reaction, strain metabolically active under assay conditions.

**Table 4:** Antibiotic sensitivity comparison of WT and *csp* deletion mutant strains corroborated Phenotype microarray determined Csp relevance in *Lm* responses against antibiotics.

**Antibiotic (E test)**	**N1546** ^**a**^		**LL195** ^**a**^		**LMNC326** ^**a**^		**EGDe** ^**a**^		
	**WT**	**∆*cspAB***	**WT**	**∆*cspAB***	**WT**	**∆*cspAB***	**WT**	**∆*cspAB***	**∆*cspABD***
Doxycyclline	0.173 ± 0.06	0.072 ± 0.01	0.253 ± 0.09	0.038 ± 0.01	0.232 ± 0.13	0.094 ± 0.02	0.252 ± 0.08	0.173 ± 0.06	0.146 ± 0.06
Trimethoprim/Sulfamethaxazole	0.013 ± 0.00	0.023 ± 0.00	0.013 ± 0.00	0.005 ± 0.00	0.011 ± 0.00	0.020 ± 0.00	0.011 ± 0.00	0.016 ± 0.01	0.014 ± 0.01
Erythromycin	0.168 ± 0.04	0.188 ± 0.06	0.273 ± 0.10	0.021 ± 0.00	0.337 ± 0.08	0.115 ± 0.02	0.157 ± 0.06	0.253 ± 0.09	0.147 ± 0.04
Gentamicin	0.147 ± 0.04	0.147 ± 0.04	0.168 ± 0.04	0.117 ± 0.02	0.110 ± 0.02	0.141 ± 0.03	0.110 ± 0.05	0.064 ± 0.00	0.138 ± 0.03

a MIC (μg/ml). Colors indicate; green; significantly reduced and red; significantly increased sensitivity of the respective *csp* deletion mutant compared to its WT strain identified using the t-test for comparison of independent samples (*p* < 0.05). Presented are mean MIC values and the standard deviations representing three biological replicates derived from E test-based antibiotic sensitivity profiling of the WT and their respective *csp* deletion mutants.

#### Strain-dependent variation of Csp roles in *Lm* chemical stress tolerance

Besides *Lm* EGDe, the elimination of *csp* genes in other strains also changed their chemical stress sensitivity profiles, either decreasing (e.g., lithium chloride) or increasing (e.g., carbenicillin and lincomycin) sensitivity (Table 5). Analogous to observations in *Lm* EGDe and its ∆*cspABD* mutant, analysis of the ∆*cspAB* mutants of *Lm* LL195 (LI) and N1546 (LII) revealed altered sensitivity to metabolic pathway, cell wall, cell membrane, and 30S ribosomal subunit targeting chemicals, confirming Csps involvement in general cell homeostasis in other *Lm* genetic backgrounds (Tables 4, 5; Supplementary Tables S2, S11). Compared to their WT strains, LL195 and N1546 ∆*cspAB* mutants were more tolerant to cell membrane targeting cationic antimicrobial peptides (e.g., colistin and polymyxin B), but more sensitive to quintenary ammonium compounds (e.g., benzethonium chloride and domiphen bromide; Table 5; Supplementary Table S11). Strain specific differences were observed regarding the impact of *csp* deletion on strain sensitivities to amoxicillin, erythromycin, sorbic acid, cetylpyridium chloride, and sulfisoxazole (Tables 4, 5; Supplementary Tables S2, S11). Such observations are suggestive of strain or genetic background-specific variations in Csp roles in stress response systems against these substances. A selection of antibiotic sensitivity profiles, revealed through PM analysis, were corroborated using E test and disc diffusion based antimicrobial sensitivity tests (Table 4; Supplementary Table S2). Relative to its respective WT, LL195_∆*cspAB* (LI) displayed increased sensitivity to trimethoprim while N1546_∆*cspAB* (LII) showed reduced sensitivity, observations indicative of genetic lineage associated differences in Csp roles in relation to trimethoprim stress tolerance mechanisms.

**Table 5:** Csp loss alters stress tolerance in different *Lm* strain genetic backgrounds.

**PM Condition** ^**a**^	**Lineage I**		**Lineage II**	
	**LL195**	**∆*cspAB***	**N1546**	**∆*cspAB***
9% NaCl	+	−	−	+
pH5	+	+	+	−
5% Sodium formate	+	−	−	+
D-Serine	+	−	+	−
Carbenicillin	+	−	+	−
Lincomycin	+	−	+	−
Spectinomycin	+	−	+	−
Domiphen bromide	+	−	+	+
Benzethonium chloride	+	−	+	+
Sorbic acid	+	−	−	+
Lithium chloride	−	+	−	+

a Presented are selected PM derived results from PM09 to PM20 evaluating osmotic, pH, and chemical stress sensitivity profiles of *Lm* LL195 and N1546 and their corresponding ∆*cspAB* mutants that express *cspD* only. Key: –, negative reaction, strain not metabolically active; +, positive reaction, strain metabolically active under assay conditions.

### Csp loss increases *Lm* sensitivity to cold and osmotic stress

**Figure 7:**
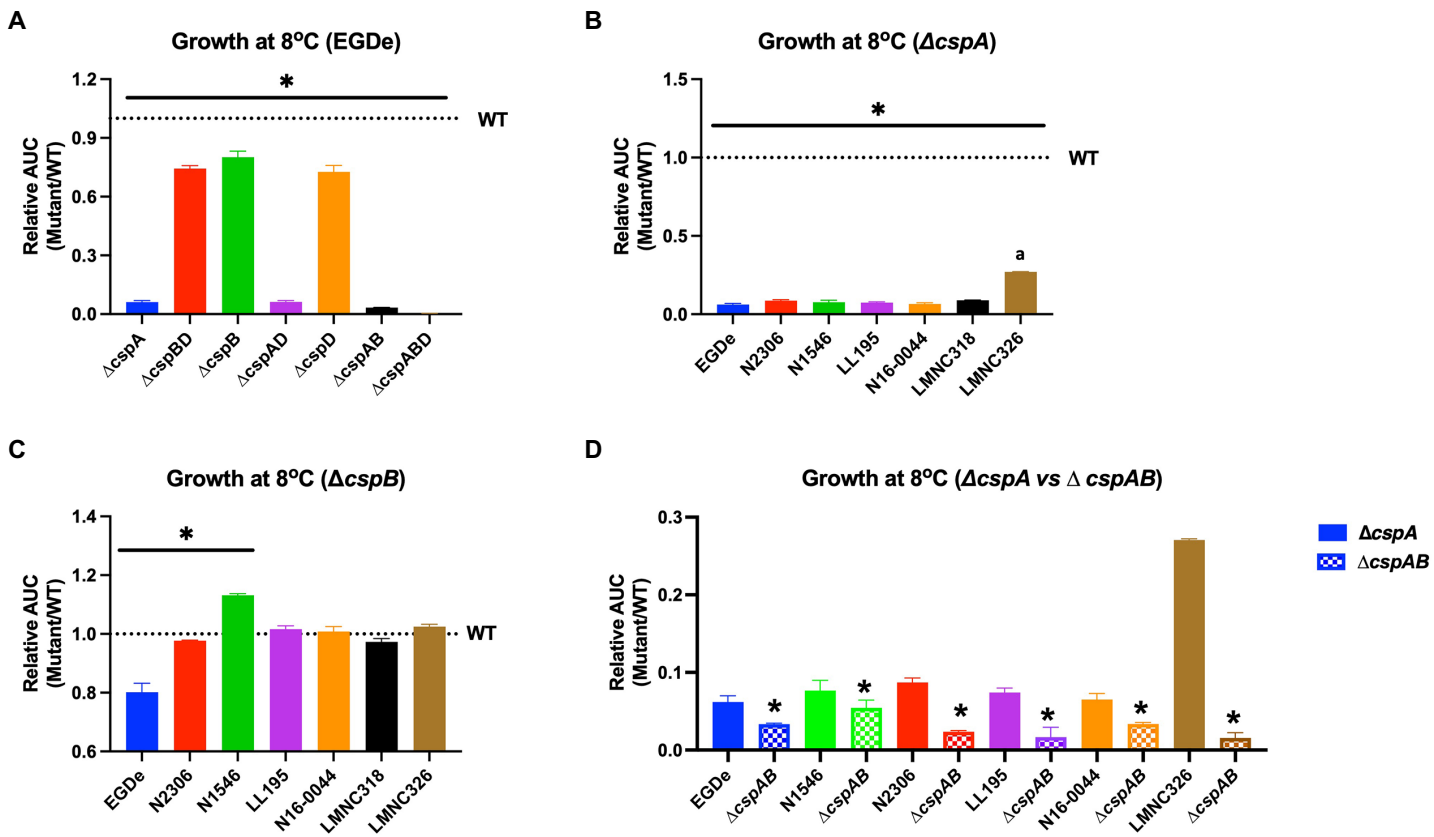
Impact of *csp* deletion on cold growth varies with *Lm* strains and molecular subtypes. Presented are mean relative area under the curve (AUC) and their corresponding standard deviation (error bars) generated from three biological replicates of OD-based growth curves comparing different *Lm* WT strains and their respective *csp* deletion mutants in BHI at 8°C. The WT strains levels are denoted by the dotted lines. **(A–C)** The star (*) and line indicate that mutants under the line are significantly different to their respective WT strains based on ANOVA and Tukey’s post-hoc test (*p* < 0.05). **(B)** The letter (a) denotes that LMNC326 is significantly different from the other strains. (**D**) * Indicates significant difference between corresponding ∆*cspA* and ∆*cspAB* mutants from the same strain determined using the t-test for comparison of independent samples.

Since *Lm* encounters cold and osmotic stress during food storage, its ability to grow under such conditions is critical for achieving infectious doses on contaminated foods. All mutants lacking *cspA* (∆*cspA* and ∆*cspAB*) in different *Lm* strains virtually lost their ability to grow at 4 and 8°C (Figure 7; Supplementary Figures S6, S7). In contrast, all ∆*cspB* mutants were able to grow albeit slower (EGDe, N2306, and LMNC318), better (N1546), or similar (LL195, N16-0044, and LMNC326) to their WT strains (Figure 7C; Supplementary Figure S6). Overall, these results confirmed *cspA* as the major *csp* gene required for cold growth across different *Lm* genetic backgrounds and revealed division of labor and hierarchical importance among the *csp* genes in cold growth. Both *cspB* and *cspD* seem less critical for cold growth in the presence of *cspA*. In *Lm* EGDe, N1546, N2306, and LL195 strains, we found out that *cspB* and *cspD* contributions to cold tolerance varied depending on the growth phase. In this case, the hierarchical *csp* gene importance for cold growth within the first week of incubation was *cspA* > *cspB* > *cspD* switching to *cspA* > *cspD* > *cspB* as incubation was prolonged (Supplementary Figure S7C–D). These strain-dependent differences in the effects of *csp* removal might be influenced by overall cold stress sensitivity variations between the WT parental strains of these mutants. For instance, *Lm* N2306 and N1546 strains were the most impaired in growth under cold stress amongst the WT strains (Supplementary Figures S6A, S7B). Meanwhile, when exposed to combined cold and NaCl (6.5%) stress conditions, which might occur on several food products, only strains expressing *cspA* (WT, ∆*cspB*, ∆*cspD,* and ∆*cspBD*) were able to grow (Supplementary Figure S8). In *Lm* EGDe, complementation of ∆*cspA* and ∆*cspABD* mutant strains with *cspA* restored growth under such dual stress, confirming *cspA* as the most important *csp* gene for *Lm* growth under dual cold and NaCl stress conditions (Supplementary Figures S7, S8). A hierarchical trend of *cspA* > *cspB* ≥ *cspD* in their importance against this dual stress was observed. Once again, *Lm* N1546 (LII, CC8) and its *csp* mutants were the most severely impaired in growth under this dual stress, with the mutants seemly performing better than the WT strain (Supplementary Figure S8). These observations with N1546 agree with increased NaCl osmotic stress tolerance observed with its *csp* deletion mutants on PM (Table 2).

### Csps act in part through regulation of *Lm* gene expression

**Figure 8:**
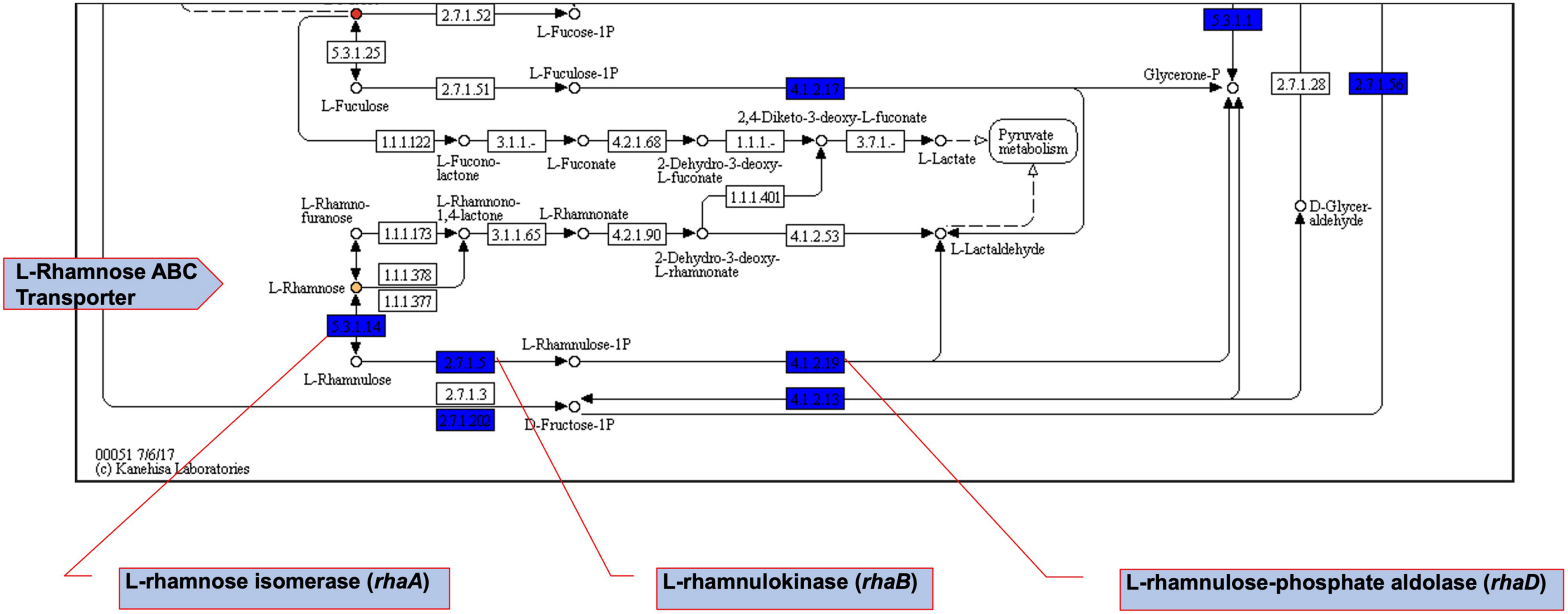
Fructose and mannose metabolism pathway map extract. Using DuctApe-based phenome-genome link analysis we identified potential Csps targets. Boxes represent reactions while circles represent compounds. Core reactions found in all strains are coloured blue, compounds present in PM plates are filled with gray. Yellow-or red-coloured circles highlight those compounds for which at least one mutant has metabolic activity significantly different with other mutants or the WT strain.

**Figure 9:**
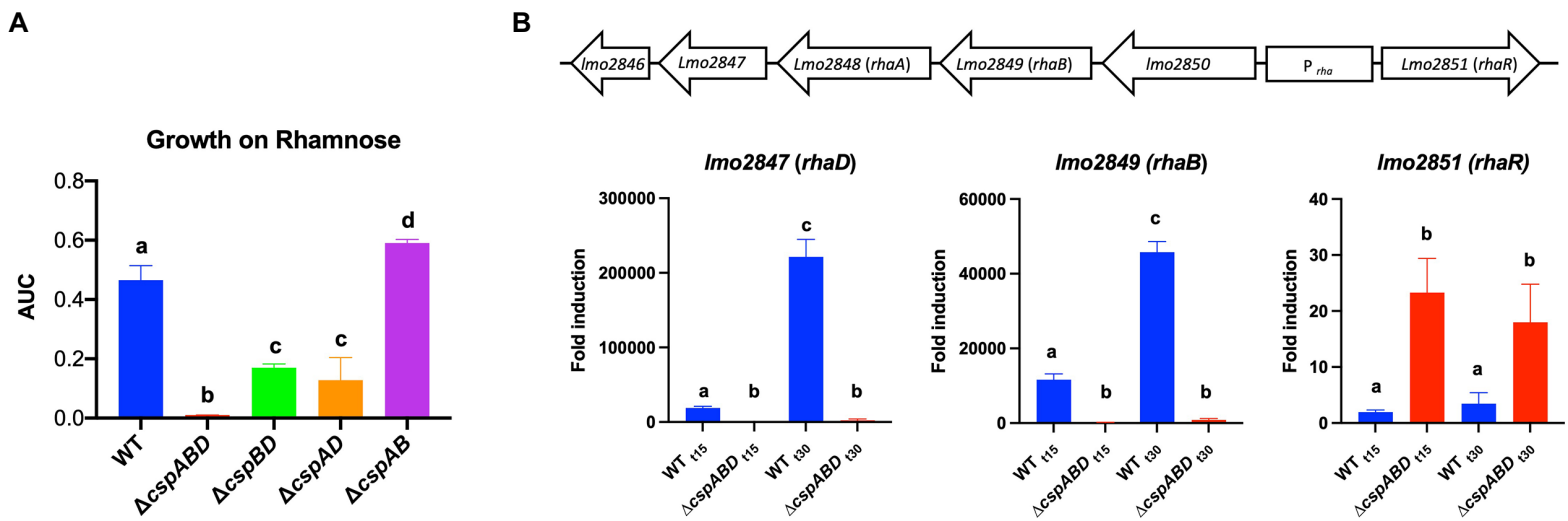
Expression of *Lm* rhamnose utilization operon genes is hindered without Csps, impairing growth on L-rhamnose as a sole C-source. (**A**) Area under the curve (AUC) bar graphs derived from OD-based growth curves for *Lm* EGDe (WT), double (∆*cspBD*, ∆*cspAD*, and ∆*cspAB*), and triple (∆*cspABD*) *csp* deletion mutants at 37°C in NB media supplemented with L-rhamnose (1% w/v) as a C-source. (**B**) Top: *Lm* rhamnose utilization operon representation modified from (Fieseler et al., 2012); below: bar graphs showing the fold induction (relative to t_0_) of *rhaB* (L-rhamnulokinase), *rhaD* (L-rhamnulose-phosphate aldolase) and *rhaR* (regulatory protein) mRNA levels using RT-qPCR in EGDe WT and ∆*cspABD* strains cultivated at 37°C in NB media containing L-rhamnose (1% w/v) as a C-source and sampled after 15 (t_15_) and 30 (t_30_) mins of incubation. Different letters denote significant differences between strains and sampling time points based on Tukey’s post-hoc test pairwise comparison following one-way ANOVA (*p* < 0.05). The data presented are means and their corresponding standard deviation (error bars) from three independent biological replicates.

**Figure 10:**
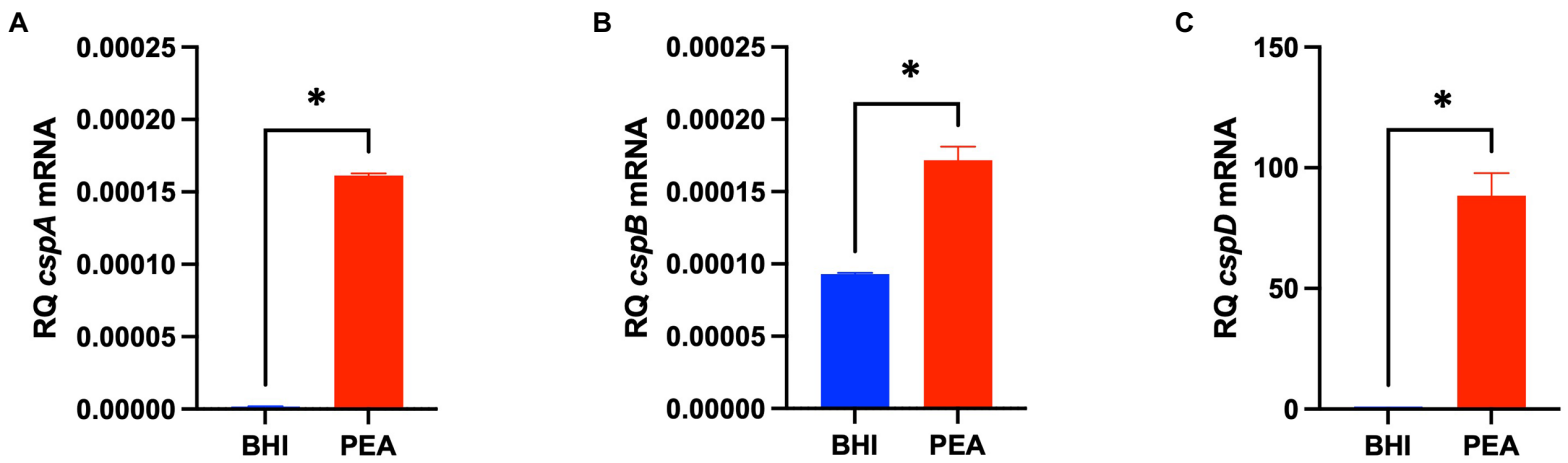
PEA stress exposure induces *Lm csp* gene expression. (**A–C**) Bar graphs showing quantification of *cspA*, *cspB*, and *cspD* mRNA levels using RT-qPCR in *Lm* EGDe cultivated 16 h at 37°C in BHI containing 0 and 6.25 mg/ml PEA. Relative quantities (RQ) of mRNA levels were normalized to 16S rRNA. The data presented are means and their corresponding standard deviation (error bars) from three independent biological replicates. *Denotes significant differences between treatments determined using the t-test (*p* < 0.0001).

The impact of Csps on gene expression was assessed to elucidate possible molecular mechanisms underlying phenotypic variability observed in *csp* mutants. Based on phenome-genome link analysis, we established that the elimination of *csp* genes in EGDe might impact expression of genes related to C-source metabolism (Figure 8). Phenotypic variability without Csps occurred on C-sources metabolized through the starch and sucrose, galactose, fructose and mannose, and pentose phosphate metabolism pathways. Similarly phenotypic variability on C-sources utilized through these pathways were noted with strains from other genetic backgrounds. Using EGDe and its *csp* deletion mutants, we confirmed that the reduced ability of *Lm* EGDe *csp* gene deletion mutants to utilize L-rhamnose is associated with altered expression of rhamnose metabolism related genes (Figures 8, 9). Gene expression analysis at mRNA level confirmed that expression of genes of the rhamnose operon, *rhaB* (*lmo284*9: L-rhamnulokinase) and *rhaD* (*lmo2847*: rhamnulose-1-phosphate aldolase) and its regulator *rhaR* (*lmo2851*) were altered in the EGDe_Δ*cspABD* mutant (Figure 9B). *rhaR* was more strongly induced in the mutant (12-fold), while *rhaB* and *rhaD* were more strongly induced in the WT strain (88-and 346-fold, respectively). Our result thus indicated that loss of Csps significantly alters expression of some C-source metabolism genes in *Lm*. To examine for molecular links between Csps and PEA stress responses, *csp* gene expression induction patterns under PEA stress were assessed, revealing that the three *csp* genes were variably induced in *Lm* EGDe cells exposed to PEA stress (Figure 10). The most upregulated *csp* gene was *cspD* (>100-fold), confirming that CspD plays a significant role in PEA tolerance. In agreement, phenotypic analysis demonstrated the highest PEA stress tolerance when *cspD* was expressed alone compared to *cspA* and *cspB* among the EGDe double *csp* gene deletion mutants (Figure 5).

### Genome differences might be responsible for variability of Csp roles across different genetic backgrounds

To identify possible reasons for differences observed in the effects of *csp* deletion in different *Lm* strain genetic backgrounds, whole genome sequence (WGS) analysis was done. Overall, *csp* genes were highly conserved, with only two of the fifty-six genomes examined displaying an amino acid changing SNP (G22S) in CspB. This suggests that the differences in roles of Csp functions are due to differences in the regulated target genes and proteins. Accordingly, various lineage and CC-associated genomic differences were found in nutrient utilization and stress tolerance genes encoding functions that were altered in the absence of Csps. For instance, in the strains used in this study, some of the enzymes involved in folate biosynthesis have genetic background-associated distribution and/or SNP-induced amino acid differences (Supplementary Table S12; Figure S9). A T91P amino acid change was detected in dihydrofolate reductase of LI strains, whereas there was a CC8-specific SNP detected in *Lm* N1546 dihydrofolate reductase that resulted in an H29Q amino acid change. Several lineage-specific amino acid altering SNPs also exist in folate biosynthesis proteins such as dihydropteroate synthase, dihydrofolate synthase or tetrahydrofolate synthase (Lmo1551), para-aminobenzoate synthetase, and molybdenum cofactor guanylyltransferase (Supplementary Table S12). Dihydroneopterin triphosphate diphosphatase [EC:3.6.1.67] distribution might have a lineage or serotype-associated trend as we have only detected it in serotype 4b strains so far. If such genes are regulated through Csps, their genetic differences might result in altered Csp-nucleic acid interactions, inadvertently altering the effects of *csp* gene removal on sensitivity to antimicrobials targeting the folate biosynthesis pathway such as trimethoprim and sulfamethoxazole.

## Discussion

Previous characterization of *Lm csp* mutants in LII CC9 (reference strain *Lm* EGDe) and CC8 (*Lm* 568 and 08–5,578) strains uncovered impaired virulence and stress resistance (reviewed by Muchaamba et al., 2021a). An extensive comparative phenotypic analysis of Csp roles in *Lm* EGDe and six additional strains representing different *Lm* genetic backgrounds (LI-III) was conducted in the current study. PM arrays and various targeted phenotypic assays were applied, examining nutrient utilization and stress resistance responses in these strains and their *csp* mutants. Expanding on the current knowledge about Csp roles in this bacterium, we have shown that Csp regulatory inputs are crucial for optimal C-source nutrient utilization and protection against cold, pH, osmotic and chemical stress conditions, including antibiotics.

*Lm* efficiently utilizes available nutrients to survive and thrive in different niches encountered in food environments and infected hosts. The ability to utilize nutrients, such as rhamnose, glucose, and palatinose, available in the environment, for instance, in decaying vegetation or contaminated food, increases *Lm* survival chances and multiplication. On the other hand, efficient utilization of glycerol and phosphate sugars promotes intracellular multiplication and virulence (Grubmüller et al., 2014). No significant growth differences were observed when *csp* deletion mutants and their corresponding parental WT strains were grown under optimal conditions at 37°C in nutrient-rich (BHI) media, confirming previous reports (Schmid et al., 2009). However, we found that under defined nutrient source conditions, the ability of *csp* deletion mutants to utilize various C-sources, including glucose, rhamnose, and glycerol, is compromised. Strain and genetic background-associated differences were also observed in the C-sources affected by *csp* absence and in view of individual Csps that played the most important role on specific C-sources. Collectively, our findings indicate that Csps regulation is crucial for the ability of *Lm* to utilize different nutrient sources. In support of such a hypothesis, we found that mRNA expression levels of some genes involved in rhamnose utilization are altered without Csps. This is consistent with observations in bacteria such as *Brucella melitensis* and *Staphylococcus aureus*, where *csp* gene deletions caused differential expression of nutrient transport and metabolism-associated genes (Wang et al., 2016; Caballero et al., 2018). Supporting *csp* involvement in *Lm* metabolism, a separate study showed that mRNA levels of pyruvate dehydrogenase complex (*pdhABC*), the enzyme that links glycolysis with the in-complete *Listeria* citric acid cycle, were significantly lower in *Lm csp* deletion mutants compared to their parent strains during desiccation (Kragh et al., 2020; Kragh and Truelstrup, 2020). Interestingly, we saw a stronger induction of the regulatory protein encoding by the *rhaR* gene in the *∆cspABD* mutant, which displayed reduced rhamnose utilization capacity compared to the WT. Though not confirmed, this might indicate that RhaR represses the *Lm* rhamnose utilization operon since *rhaB* and *rhaD* were strongly induced in the WT, which had lower *rhaR* mRNA abundance compared to the mutant. Similarly, RhaR acts as a repressor of the rhamnose utilization operon in *B. subtilis* (Hirooka et al., 2015). Overall, our data indicate that Csps play critical roles in C-source metabolism pathway regulation in *Lm*. Going forward, a more global transcriptomic and proteomic approach is therefore warranted to establish in more detail the mechanistic roles linking Csps to metabolism or nutrient utilization pathways in this bacterium. Understanding such interactions might, in the future, lead to novel hurdle techniques targeting Csps or their regulatory targets to disrupt nutrient utilization, reducing *Lm* growth potential on food and within infected hosts.

*Lm*’s ability to metabolize glycerol, a key intracellular C-source, influences growth within the host, ultimately affecting pathogenicity (Joseph et al., 2006; Grubmüller et al., 2014). Across the tested study strains, the removal of *cspA* and *cspB* reduced glycerol utilization, with the most severe reduction observed for the triple deletion mutant (∆*cspABD*). This impaired glycerol utilization and the previously described reduced expression of key virulence factors such as PrfA and LLO might, therefore, in part explain why EGDe double (Δ*cspBD*, Δ*cspAB*, Δ*cspAD*) and triple (Δ*cspABD*) *csp* gene deletion mutants are hypovirulent and avirulent, respectively (Eshwar et al., 2017). Reduced glycerol utilization could also contribute to the reduced growth and survival of these EGDe ∆*csp* mutants observed within macrophages (Loepfe et al., 2010; Eshwar et al., 2017). Expression of virulence factors is tightly regulated and is minimized outside the host. On PMs, most WT strains in this study did not metabolize D-glucose-6-phosphate, another important intracellular C-source. An exception was EGDe WT and its ∆*cspBD* and ∆*cspABD* mutants, which utilized this C-source, albeit EGDe WT doing so weakly. The intracellular utilization of D-glucose-6-phosphate is in part facilitated by the expression of Hpt permease, which is tightly regulated by the central virulence regulator PrfA (Ripio et al., 1997; Chico-Calero et al., 2002; Joseph et al., 2006). The ability of ∆*cspBD* and ∆*cspABD* to utilize this hexose phosphate under extracellular conditions might indicate dysregulation of *hpt* gene expression in these mutants. These observations thus suggest that Csps contribute to fine-tuning nutrient utilization and virulence gene expression regulation in *Lm*.

Hurdle procedures such as low temperatures, low pH, and osmotic stress are applied to minimize bacteria food contamination and growth (Wiktorczyk-Kapischke et al., 2021). *Lm* adapts and grows under such harsh conditions through mechanisms that include H^+^ ion efflux pumps, cell membrane content and structural changes, and accumulation of compatible solutes (Angelidis and Smith, 2003; Wemekamp-Kamphuis et al., 2004; Soni et al., 2011; Singh et al., 2011; Wiktorczyk-Kapischke et al., 2021). Improvement of hurdle procedures against this pathogen requires full a understanding of the mechanisms underlying these stress resistance phenotypes. We have revealed and confirmed Csps contributions to cold, osmotic, and pH stress tolerance in other strains and genetic backgrounds beyond the *Lm* reference strain EGDe (Schmid et al., 2009). The different *csp* deletion mutants examined differed in tolerance to cold, pH, and osmotic stress in comparison to their WT strains, with the mutants being more sensitive to most of these stresses. These results corroborate earlier findings (Schmid et al., 2009) on EGDe and its *csp* null mutants, which also revealed significant variation in resistance to cold and osmotic stress conditions that are relevant to host and food-associated environments.

*Lm* cross-protection against several hurdle techniques, including pH, osmotic, and cold stress, has been observed (Bergholz et al., 2012, 2013; Begley and Hill, 2015; Burgess et al., 2016; Abeysundara et al., 2019; Wiktorczyk-Kapischke et al., 2021; Wu et al., 2021). Such a phenomenon might involve the activation of common genes and proteins under these stresses. Measures for cold stress tolerance include the accumulation of compatible solutes and alterations to cell membrane structure that are also employed against pH and osmotic stress (Gandhi and Chikindas, 2007; Abeysundara et al., 2019; Wu et al., 2021). Csps, by virtue of their involvement in tolerance of these stressors might therefore contribute to the regulation of molecular mechanisms underlying such stress cross-protection responses. CspA might be one of the Csps regulating such stress cross-protection responses, as we demonstrated here that it is the main Csp contributing to *Lm* growth under dual cold and osmotic stress conditions. CspA functions alone, however, are not sufficient to achieve the WT phenotype level, indicating that an overall coordinated function of all three Csps is necessary for full protection against combined cold and osmotic stress. CspB and CspD functional contributions under such conditions are dependent on CspA, as when expressed alone, these two Csps were not capable of restoring any detectable *Lm* growth under these dual stress conditions. Meanwhile, the fact that Csps promote *Lm* adaptation against pH, cold, and osmotic stress has significant implications in view of food safety measures. Since combined or sequential exposure of *Lm* to these three stresses within food environments might inadvertently induce cross-protection responses through activation of Csp expression. Substances such as betaine, creatine, creatinine, L-carnitine, and D-trehalose improved NaCl stress tolerance of *Lm* EGDe and its *csp* deletion mutants. Notably, the EGDe WT strain utilized such substances better than its *csp* mutants, indicating that Csps contribute to optimal expression regulation or functional efficiency of proteins involved in the utilization of these compounds such as glycine betaine (BetL and Gbu) and carnitine (OpuC) transporters (Wemekamp-Kamphuis et al., 2004; Burgess et al., 2016; Bucur et al., 2018). The most significant variability observed between *Lm* WT and the ∆*csp* strains occurred during growth under NaCl (6%) stress upon D-trehalose supplementation. Interestingly, trehalose accumulation or overproduction provides important osmo-and stress protectant functions in *Lm* and other bacteria such as *Escherichia coli* and *Ralstonia solanacearum* (Purvis et al., 2005; Ells and Truelstrup, 2011; MacIntyre et al., 2020). In the future, analysis of the effects of Csp deficiency on pathways associated with trehalose metabolism is therefore, warranted. Meanwhile, *Lm* strains from other genetic backgrounds examined here showed no significant differences between their *csp* mutants and WT strains when grown under NaCl (6%) stress supplemented with most of the tested compatible solutes. Possible explanations could be that Csps do not influence the expression of compatible solute transporters in these genetic backgrounds or that the strains used are more tolerant to osmotic stress compared to EGDe. Systems for accumulating compatible solutes in these strains might only become critical or strongly induced at higher NaCl stress levels than those applied here.

In the journey from the environment through food until the host cell, *Lm* is confronted with pH stress in acidic foods, during gastric passage and in the macrophage phagosomes (Cotter and Hill, 2003; Gahan and Hill, 2014). *Lm* overcomes these obstacles by utilizing several stress adaptation strategies (Gandhi and Chikindas, 2007). Csps have been implicated in pH stress response in other bacteria, including *C. botulinum* (Derman et al., 2015; Keto-Timonen et al., 2016). We also established here that there are functional contributions of Csps towards *Lm* pH stress tolerance responses, which might thus also influence growth and survival of *Lm* on low pH foods as well as during transit within the gut and macrophages to influence infection establishment. Since Csps are induced under cold and osmotic stress, these observations imply that cold or osmotic stressed bacteria could be more tolerant to acid stress, which might affect the efficiency of combining these food preservation techniques due to Csp-mediated cross-protection. Prior exposure to acid and osmotic stress has been postulated to prime *Lm* against gastric stress (Barbosa et al., 2012; Pettersen et al., 2019), Csps might thus also play a critical role in this process. Although the mechanisms of action for Csp involvement in most of the above-mentioned stress responses are yet to be elucidated, it is plausible that Csps might affect function or expression of the compatible solute transporter systems, cell membrane component biosynthesis and modification pathways, and ion or proton pumps. Meanwhile, their chaperone activity might facilitate damaged DNA repair or altered DNA and RNA structures ensuring transcription and translation progression (Phadtare, 2004; Keto-Timonen et al., 2016).

To better prevent and control antimicrobial resistance (AMR), an improved understanding of mechanisms giving rise to increased antibiotic tolerance is essential. *Lm* is not well-known for AMR, however, recent studies have demonstrated AMR for some strains (Olaimat et al., 2018; Kayode et al., 2021). Similar to previous observations in other bacteria (Etchegaray and Inouye, 1999; Cruz-Loya et al., 2019), we also found that Csps contribute to the modulation of antibiotic stress tolerance in *Lm*. In *Lm* EGDe, LL195, and N1546 strains, we observed that *csp* mutants were less tolerant to various chemicals and antibiotics, which target multiple systems, including protein synthesis, cell wall, cell membrane, and general homeostasis systems. Such observations thus confirm that Csps in this bacterium are involved in the regulation of various other response pathways beyond nutrient utilization and general stress response systems. Moreover, the emergence and spread of increased tolerance against cleaning and disinfection chemicals, such as quaternary ammonium compounds, in *Lm* is greatly concerning (Meier et al., 2017). We also observed that Csps contribute to responses against such stressors, implying that conditions that induce *csp* expression might increase tolerance to such disinfectants. Combined use of chemicals or conditions that downregulate *csp* gene expression and disinfectants might result in improved decontamination efficiency. At the mechanistic level, *csp* mutants were more sensitive to the cell wall and membrane-targeting substances such as cloxacillin and BC, suggesting a role for Csps in the proper function of these cellular systems. Recently, we demonstrated that, among other functions, Csps also affect the expression of Penicillin-binding protein genes and cell wall modification systems contributing to such stress responses (Muchaamba et al., 2021b). An explanation is that *csp* mutants could have altered cell wall structures that increase permeability to some chemicals and antibiotics thus increasing sensitivity. Furthermore, the cell envelope in a Δ*cspABD* mutant was more negatively charged than the WT, enhancing interaction and sensitivity to positively charged stressors such as nisin and BC (Muchaamba et al., 2021b). In the present study, the *csp* mutants were also more sensitive to 30S subunit targeting inhibitors suggesting that this subunit might be the Csps site of action on the ribosomes and that Csps might be involved in the initiation steps of protein synthesis (Weber et al., 2001).

On the other hand, *csp* mutants were more tolerant than the WT on some substances, e.g., EGDe_∆*cspABD* was more tolerant than its WT on five substances. We also previously found that *cspD* expressed alone in an EGDe ∆*cspAB* mutant increased tolerance to antimicrobials such as nisin and daptomycin (Muchaamba et al., 2021b). The mechanisms underlying these phenotypes are unknown, but we can only currently speculate that Csp loss might cause an alteration in expression of genes that increase resistance. Slower growth rates can increase tolerance to some antibiotics (Pontes and Groisman, 2019). EGDe_∆*cspABD*, N1546_∆*cspAB*, and LL195_∆*cspAB* showed slightly slower growth than their respective WT strains, which might have contributed to the increased tolerance of these mutants against some of the tested chemical or antibiotics. Overall, the data generated from these assays might provide valuable insights in the development and application of novel treatment and disinfection protocols against *Lm.* Identification of chemicals under which the *csp* deletion mutants outperformed the WT, e.g., ketoprofen can be exploited in the search for new antimicrobials and/or in listeriosis patients requiring drugs with analgesic and antipyretic effects.

Gain of function upon loss of some *csp* genes was observed on some C-sources and stress conditions. An EGDe_∆*cspAD* mutant, that only expresses *cspB,* for instance, gained functions allowing better utilization of 25 C-sources, while the EGDe_∆*cspABD* mutant became more tolerant than its WT to five chemical or antibiotic stressors. These observations are, therefore, suggestive of regulatory crosstalk between the Csps. Thus, Csps might have a negative or positive regulatory loop feedback system where overexpression or downregulation of one *csp* affects the expression levels of itself and other Csps. In the case of EGDe, CspA and CspD might be inhibitory to *cspB* expression or functional influences, consequently, there is gain of function of systems that CspB might positively regulate without *cspA* and *cspD* in EGDe_∆*cspAD*. A compensatory increase in *cspB* and *cspD* transcripts in *cspA* deletion mutants compared to their WT was previously observed in response to desiccation (Kragh et al., 2020). This might also explain increased tolerance observed for ∆*cspA* mutants exposed to PEA, for which we have also demonstrated CspD and CspB to be the most important Csps contributing to its tolerance. Interestingly, a similar phenomenon was observed under cold stress where *cspB* removal in strains *Lm* N1546 slightly enhanced cold growth capability. Absence of CspB in such strains might have resulted in compensatory upregulation of *cspA* and *cspD*, which are known to be the most essential for cold growth. This phenomenon was also observed in other bacteria species (Bae et al., 1997; Graumann et al., 1997; Keto-Timonen et al., 2016), indicating that Csps regulate themselves and each other’s expression, possibly in a negative feedback loop manner as observed in *S. aureus* and *E. coli* (Bae et al., 1999; Caballero et al., 2018; Catalan-Moreno et al., 2021). *Lm* cells previously exposed to a hurdle procedure that lowers CspA or induces CspD expression might thus be rendered more tolerant to anti-*Lm* interventions such as nisin and PEA addition (Muchaamba et al., 2021b). Such knowledge would be critical in the design of hurdle technique combinations to establish which procedures might result in cross-protection or increased pathogen susceptibility. A triple *csp* deletion (∆*cspABE*) in *Lactococcus lactis* did not affect growth characteristics of the bacterium, an observation attributed to increased expression of the remaining *csp* genes (Wouters et al., 2000). This functional redundancy means that a phenotype might only be observed when a significant number or all *csp* genes of a bacterium are deleted. As an example, removal of a single *csp* gene increased sodium benzoate tolerance (∆*cspA* > ∆*cspD* > ∆*cspB*), while deletion of more than one *csp* gene increases sensitivity to this stress. An exception being that the double deletion of *cspB* and *cspD* (∆*cspBD*) in EGDe demonstrated slightly increased sodium benzoate tolerance compared to the WT. These data are indicative of both epistatic interactions and functional hierarchies between individual *csp* genes regarding stress tolerance.

It seems that an intricate system of different transcriptional regulators and mechanisms exists to fine-tune *csp* gene expression under various conditions. Under osmotic stress, σ^B^ negatively regulates *cspD*, while in late logarithmic phase cultures σ^L^ assumes this role (Arous et al., 2004; Chaturongakul et al., 2008; Raengpradub et al., 2008). Conversely, HrcA, a stress response gene regulator, indirectly upregulates *cspD* expression (Hu et al., 2007; Chaturongakul et al., 2008). In strain *Lm* N2306, which encodes a truncated σ^B^ (Muchaamba et al., 2022), *csp* deletion mutants displayed increased sensitivity phenotypes compared to the other tested strains on conditions were CspD or other Csps are relevant, e.g., NaCl, sodium formate, and sodium lactate stress. Such phenotypic differences might thus be related to disrupted σ^B^ regulation of Csps and other genes in this strain.

The development of selective chromogenic media for detecting *Lm*, such as OCLA agar, relies on specific patterns in chromogenic substrate catabolism (Willis et al., 2006). We found here that the deletion of *csp* genes altered chromogenic substrate utilization efficiency on PMs and OCLA plates. Csps might have regulatory or functional roles affecting pathways or proteins such as β-glucosidases involved in the utilization of these chromogenic substances. Thus, chromogenic media developers must be aware of the possible limitations of identification or isolation procedures that incorporate selective pressure, which might alter *csp* expression, inadvertently affecting chromogenic substrate utilization.

WT strains displayed strain and genetic background-associated phenotypic differences under some conditions, e.g., allose utilization, osmotic and cold stress sensitivity. Therefore, some strain and/or phylogenetic diversity in *Lm csp* gene expression regulatory roles is expected. Previous studies demonstrated different functional roles and phenotypes regarding desiccation and biofilm production when *csp* genes were deleted from strains representing different genetic backgrounds (Kragh et al., 2020). Here we also demonstrated differences in the effects of *csp* removal on C-source utilization, cold, pH, osmotic, chemical, and antibiotic stress in different genetic backgrounds. Differences in gene absence-presence patterns as well as strain and genetic background-associated SNPs and InDels might result in altered Csp/DNA/mRNA/protein interactions, ultimately resulting in the different phenotypes observed. The differences in gene absence-presence patterns might result in the activation of different alternative pathways which might also result in these altered phenotypes. For example, we observed here that while a ∆*cspAB* mutation in LI strain LL195 increased sensitivity to folate biosynthesis targeting antibiotics, it caused reduced sensitivity to these antibiotics in the LII (EGDe and N1546) and LIII (LMNC326) strains. Strain and genetic background-specific gene absence-presence patterns and SNP-induced amino acid changes in proteins of the folate biosynthesis pathway in these LI, LII, and LIII strains could be underlying the varied effects observed upon *csp* deletion. Inversely, some Csp functions were maintained across all strains. For instance, *cspA* deletion resulted in increased cold stress sensitivity in all strains regardless of their molecular subtype or genetic background. However, strain-specific trends were observed regarding *cspB* contributions to cold stress tolerance. Opposing effects were sometimes observed upon the removal of *csp* genes in strains from the same lineage, for instance, *cspA* removal increased NaCl stress tolerance in N1546 (LII; CC8) but decreased it in EGDe (LII; CC9). Our data are suggestive of strain-dependent functional diversity of Csps roles, possibly due to the evolutionary differences in components of the stress response systems in these strains. Our findings thus highlight Csp phenotypic role conservation and diversity among strains from different *Lm* genetic backgrounds, supporting the need for future analysis of Csp roles to be conducted using strains from different genetic backgrounds, especially those of greater public health and food safety importance.

Functional redundancy is postulated to be a failsafe strategy to ensure that important phenotypes are conserved (Meier et al., 2017; Muchaamba et al., 2021a). *Lm* Csps display functional redundancy on several phenotypes. For instance, all Csps are involved in PEA stress tolerance, albeit at differing levels. PEA exposure induced expression of all three *csp* genes, although at different intensities. CspA is inhibitory, while CspB and CspD promote resistance to this stress in the hierarchy CspD>CspB. On the other hand, true to their name, all Csps promote tolerance to cold stress with the hierarchy CspA>CspD≥CspB. *Lm* Csps functional redundancies have also been previously demonstrated using EGDe and its ∆*csp* mutants on other phenotypes, including virulence, flagella-based motility, stress tolerance, and biofilm production (Schmid et al., 2009; Loepfe et al., 2010; Schärer et al., 2013; Eshwar et al., 2017; Kragh et al., 2020). The extent of this redundancy is, however, varied with the phenotype in question and strain. For example, single removal of either *cspA* or *cspB* alone in all strains had minimal to no effect on glycerol utilization. Based on this, it can be assumed that the impacts of individual *csp* genes deletion on glycerol metabolism pathways are minimal due to functional redundancies. This Csp functional redundancy is probably due to their high nucleotide and amino acid sequence conservation resulting in similar regulatory targets but having different influence intensities.

On cold growth, we observed phase variation in the importance of individual Csps. In the early phases of growth, the trend *cspA* > *cspB* > *cspD* differed from the trend *cspA* > *cspD* > *cspB* observed previously by Schmid et al. (2009) using the same strains and mutants. Growth measurements by Schmid et al. (2009) were done weekly while in our study, assessments were done every 24 to 48 h. Additionally, starting inoculums differed 10^3^ vs. 10^7^ CFU/ml in the old and current studies, respectively. These experimental setup differences allowed us to detect the early contributions of CspB to cold growth, which might have been missed in the previous study. After 7 days of incubation at 8°C, the cell density of the ∆*cspB* mutant was comparable with the WT, in agreement with the previous study. Another possible explanation is the influence of the inoculum. In the absence of CspB, the high starting cell numbers might have delayed cold stress adaptation, a feature probably influenced by quorum sensing. We have now thus refined knowledge on individual Csp involvement in cold growth. The trend CspA>CspB>CspD holds true for early phases of growth, while the trend CspA>CspD>CspB applies for later phases of growth, probably beyond the lag phase. Overall, CspA is the most critical for cold growth across all tested strains. This agrees with previous work, which observed that when *Lm* EGDe is cold shocked, *cspA* is significantly more upregulated compared to *cspB* and *cspD*, with *cspB* being the least induced (Schmid et al., 2009).

A caveat in assessing the functional role of individual *csp* genes in *Lm* using single gene deletion mutant backgrounds is that some phenotypes might be masked due to functional redundancy that exists between the three *csp* genes found in this bacterium. Therefore, data generated with single *csp* gene deletion mutants must be interpreted with caution as they might be influenced by these epistatic interactions and functional redundancies. EGDe_∆*cspABD* analysis allowed us to get a global overview of the regulatory contributions of *csp* genes unabated by this redundancy and epistasis. The ∆*cspABD* mutants for the other study strains are not yet available hence the full contributions of Csps in such strains are yet to be determined. Nonetheless, the currently available mutants have allowed for significant advancement of our understanding of Csp roles in other strains beyond EGDe. By extrapolation from other organisms and current data in *Lm*, Csps exert their influence through interacting with DNA and RNA altering gene expression or increasing mRNA stability (Schärer et al., 2013; Keto-Timonen et al., 2016; Eshwar et al., 2017; Meier et al., 2017).

In conclusion, this study has demonstrated that Csps have multiple and complex functions, either up- or down-regulating metabolic processes. We showed involvement of Csps in nutrient utilization, chemical, antibiotic, cold, pH, and osmotic stress tolerance responses. CspA seems to be most important for cold and osmotic stress tolerance. It is also interesting that in other stress situations, the removal of *cspA* enhanced tolerance, suggestive of CspA having a possible negative regulatory effect on some of the stress response systems involved. It might also be that systems positively upregulated by CspA are high energy consuming hence in its absence the cell has more resources to divert to stress responses. Combining virulence data and significant expansion of nutrient utilization capacity in strains expressing *cspB* only observed in this study and those by others (Eshwar et al., 2017), it seems CspB is more important for nutrient utilization or metabolism and virulence. CspD seems to play a more important role in stress tolerance against conditions such as osmotic, chemical, and antibiotic stress. Involvement of Csps in such processes has significant public health implications since some interventions might lead to their induction and induce cross-protection against other stress factors or prime the pathogen for host pathogenicity. Such knowledge paves way for improved design and application order and/or combination of hurdle techniques in the fight against *Lm* in food. Overall, the knowledge gained in this study can be used to optimize anti-*Listeria* hurdle procedures and create new strategies and interventions to control this pathogen, thus improving food safety. Moreover, data on growth inhibitors can potentially be exploited for development of novel anti-*Listeria* food additives.

## Data availability statement

The original contributions presented in the study are included in the article/Supplementary material, further inquiries can be directed to the corresponding author.

## Author contributions

FM and TT designed the study. TT, RS, and UA supervised the study. FM performed all the experiments, analyzed the data, wrote the first manuscript draft, and did final editing and review consolidation. TT and UA assisted in the experiments. Bioinformatic analyses were conducted by FM and MS. RS, UA, MS, and TT analyzed the data and edited the manuscript. All authors contributed to the article and approved the submitted version.

## Funding

The work was supported by the University of Zürich and Agroscope, Switzerland and done as a collaboration between the two organizations.

## Conflict of interest

The authors declare that the research was conducted in the absence of any commercial or financial relationships that could be construed as a potential conflict of interest.

## Publisher’s note

All claims expressed in this article are solely those of the authors and do not necessarily represent those of their affiliated organizations, or those of the publisher, the editors and the reviewers. Any product that may be evaluated in this article, or claim that may be made by its manufacturer, is not guaranteed or endorsed by the publisher.

## Footnotes

^1^ https://biolog.com

^2^ https://www.biolog.com/products-portfolio-overview/phenotype-microarrays-for-microbial-cells/

^3^ http://rast.nmpdr.org/

^4^ blast.ncbi.nlm.nih.gov/Blast.cgi

^5^ https://bigsdb.pasteur.fr/listeria
