# Supplementary material for: Deciphering the global roles of Cold shock proteins in Listeria monocytogenes nutrient metabolism and stress tolerance
Source: Front Microbiol. 2022 Dec 20;13:1057754. doi: 10.3389/fmicb.2022.1057754 (PMC9808409; doi:10.3389/fmicb.2022.1057754)
Supplement: Supplementary file 1 [file Presentation_1.PPTX]

## Slide 1
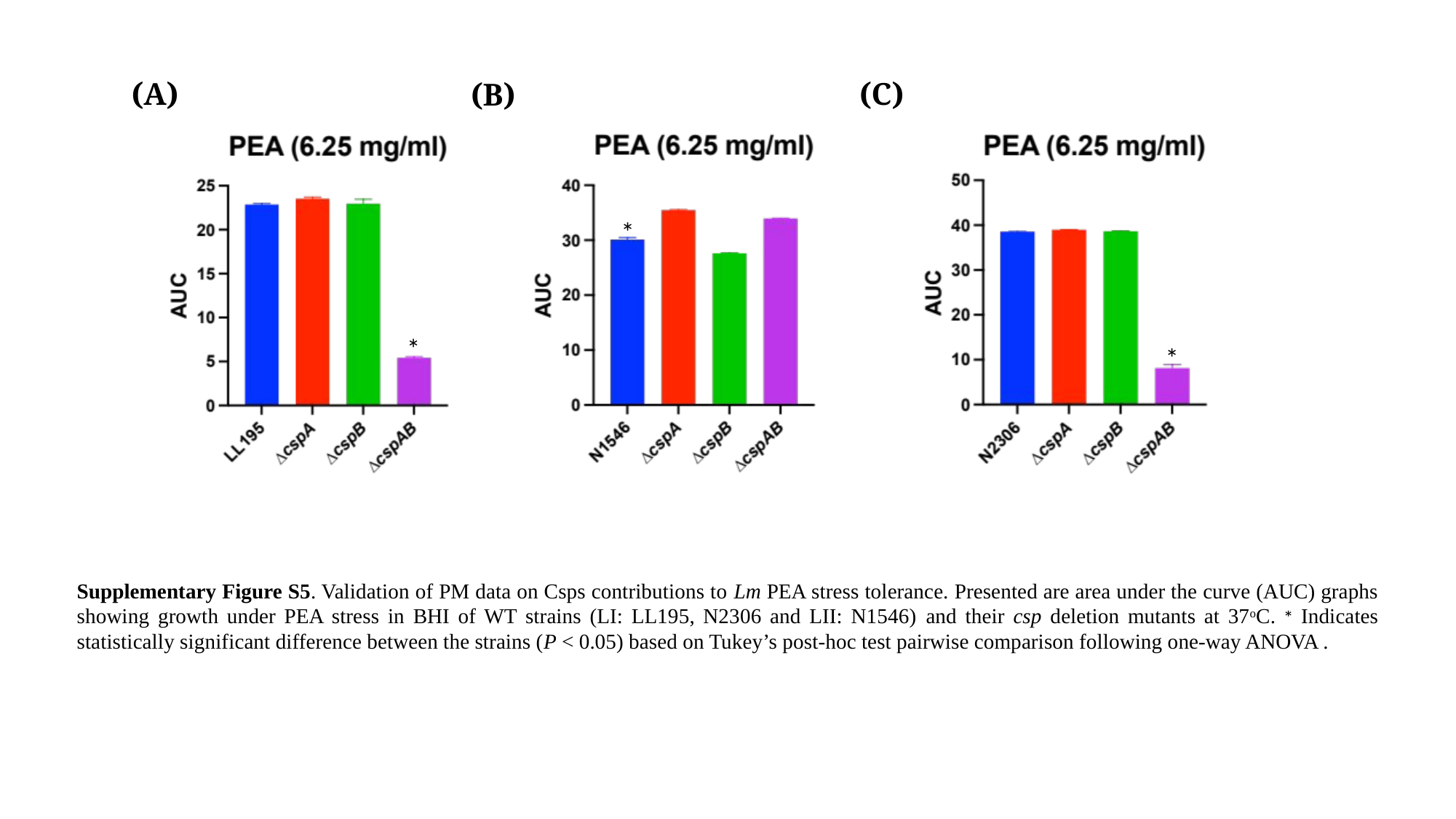

(A)
(C)
(B)
*
*
*
Supplementary Figure S5. Validation of PM data on Csps contributions to Lm PEA stress tolerance. Presented are area under the curve (AUC) graphs showing growth under PEA stress in BHI of WT strains (LI: LL195, N2306 and LII: N1546) and their csp deletion mutants at 37oC. ∗ Indicates statistically significant difference between the strains (P < 0.05) based on Tukey’s post-hoc test pairwise comparison following one-way ANOVA .

## Slide 2
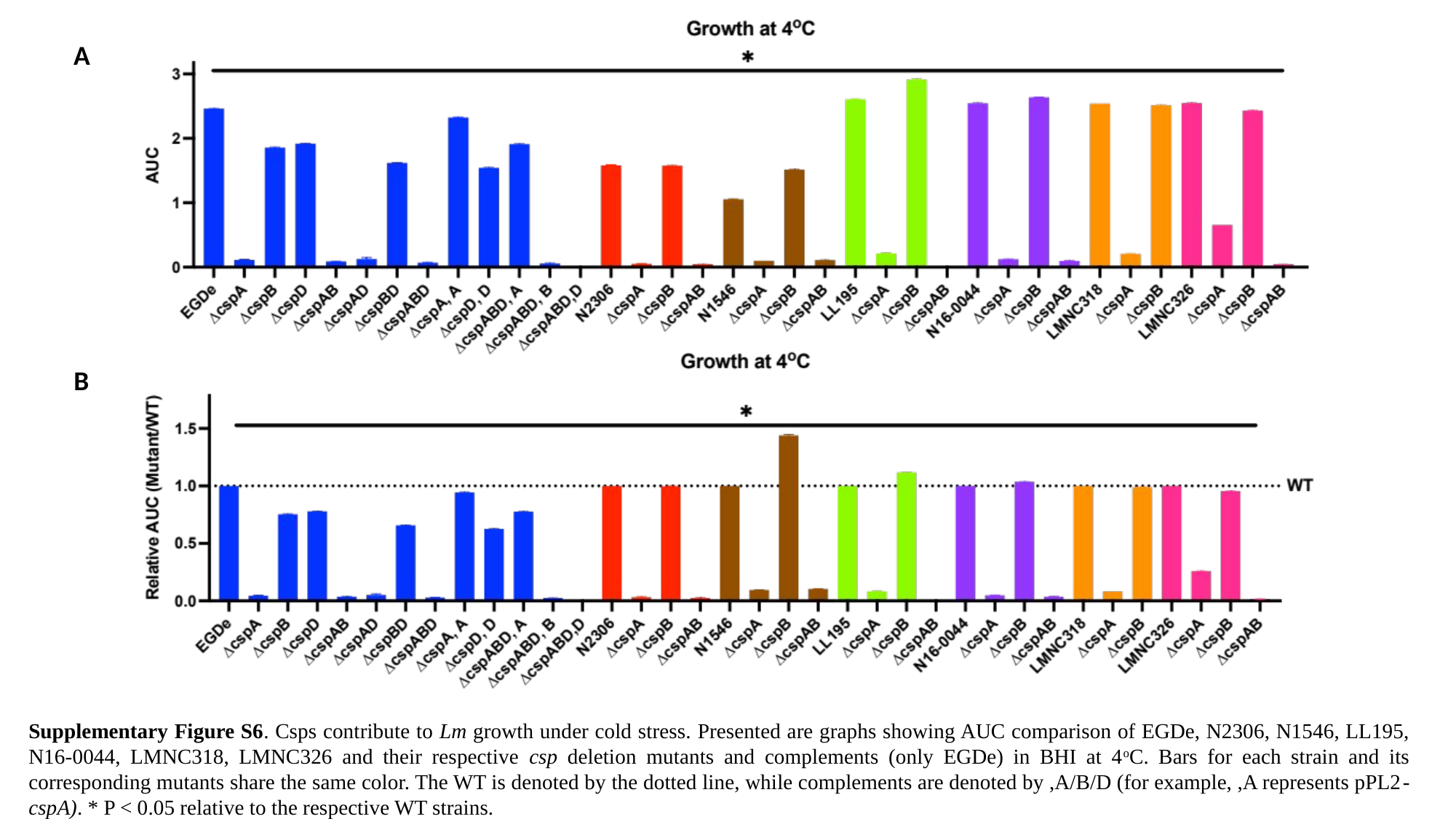

A
B
Supplementary Figure S6. Csps contribute to Lm growth under cold stress. Presented are graphs showing AUC comparison of EGDe, N2306, N1546, LL195, N16-0044, LMNC318, LMNC326 and their respective csp deletion mutants and complements (only EGDe) in BHI at 4oC. Bars for each strain and its corresponding mutants share the same color. The WT is denoted by the dotted line, while complements are denoted by ,A/B/D (for example, ,A represents pPL2-cspA). * P < 0.05 relative to the respective WT strains.

## Slide 3
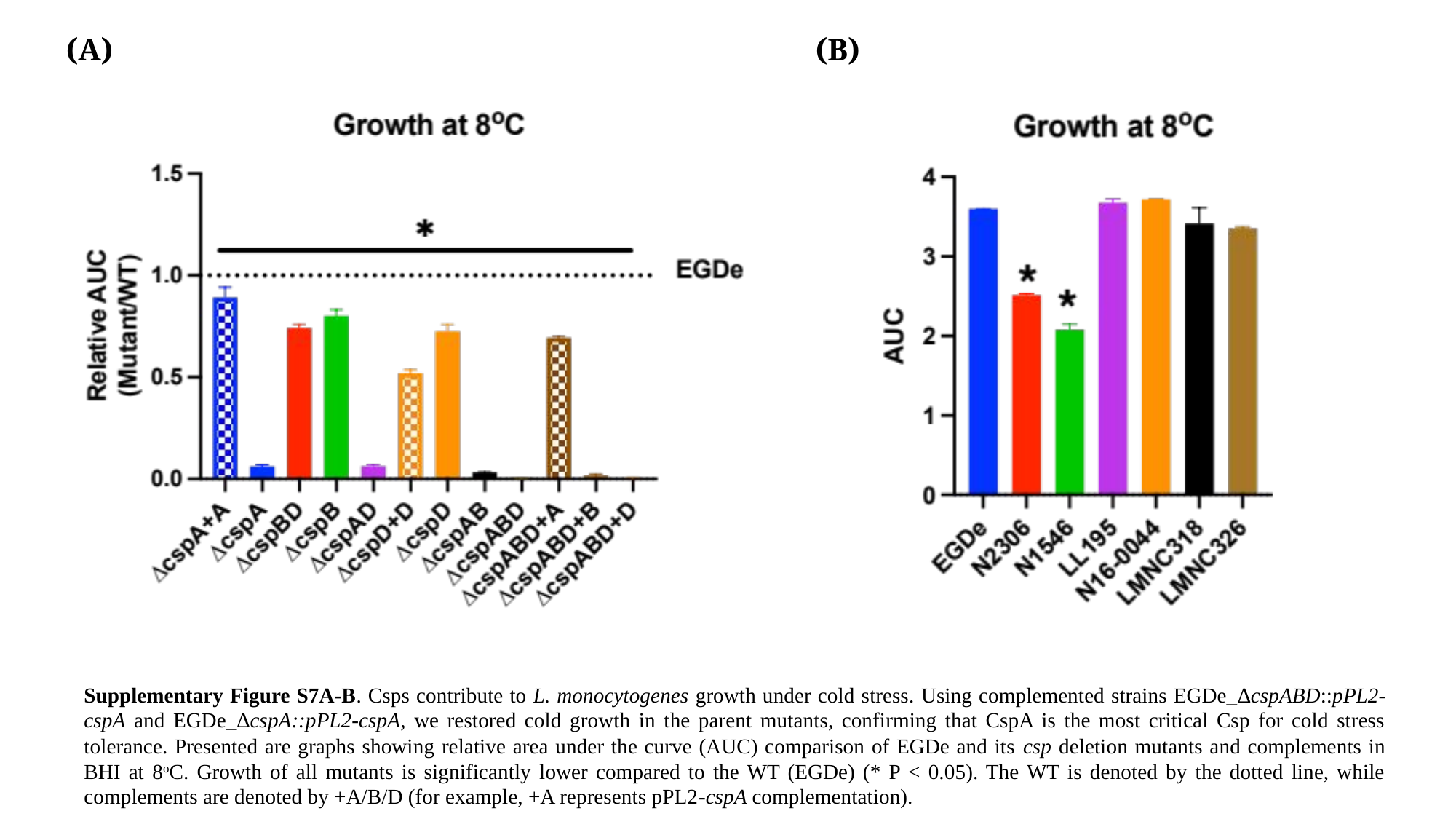

(A)
(B)
Supplementary Figure S7A-B. Csps contribute to L. monocytogenes growth under cold stress. Using complemented strains EGDe_∆cspABD::pPL2-cspA and EGDe_∆cspA­::pPL2-cspA, we restored cold growth in the parent mutants, confirming that CspA is the most critical Csp for cold stress tolerance. Presented are graphs showing relative area under the curve (AUC) comparison of EGDe and its csp deletion mutants and complements in BHI at 8oC. Growth of all mutants is significantly lower compared to the WT (EGDe) (* P < 0.05). The WT is denoted by the dotted line, while complements are denoted by +A/B/D (for example, +A represents pPL2-cspA complementation).

## Slide 4
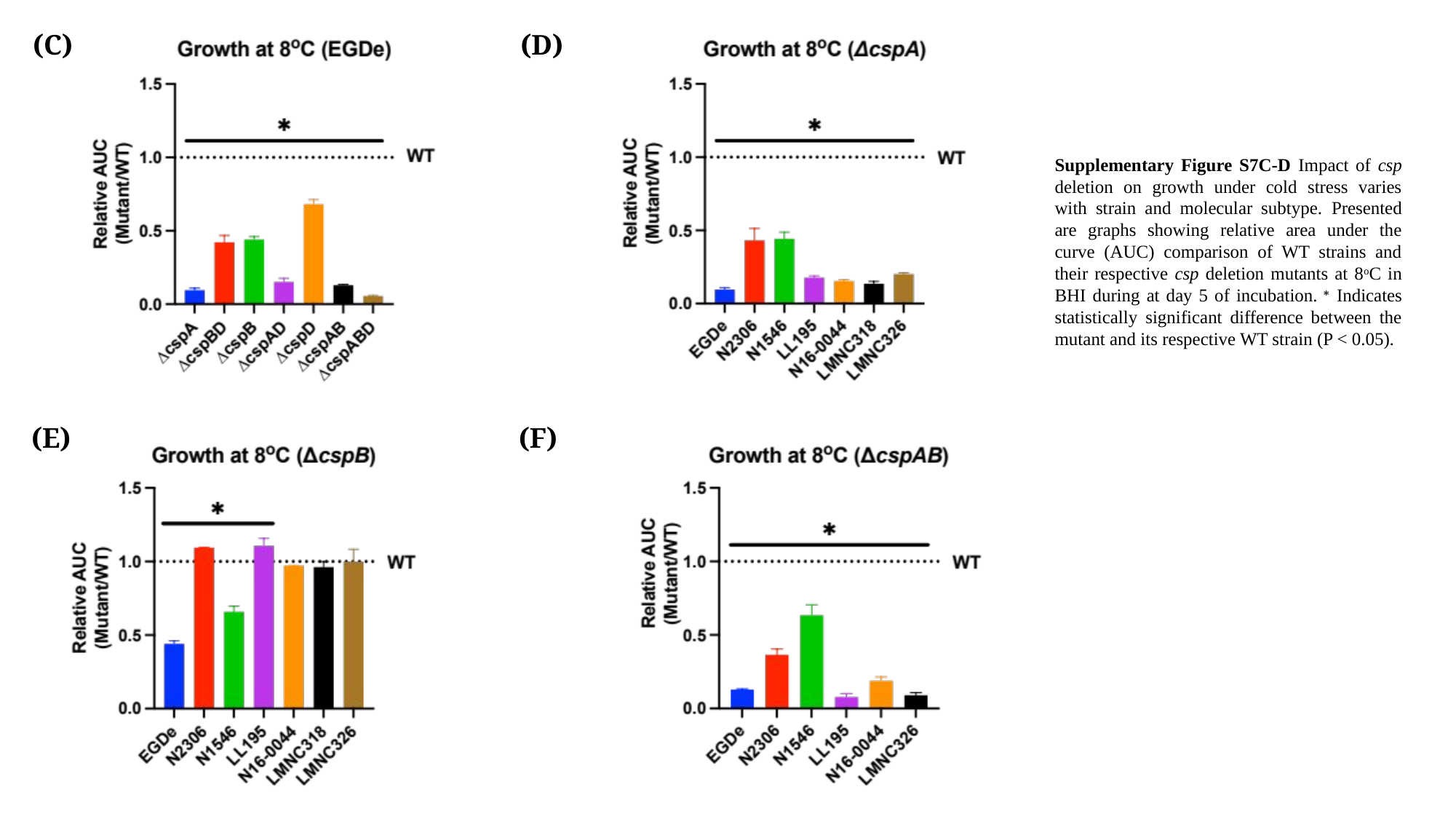

(C)
(D)
Supplementary Figure S7C-D Impact of csp deletion on growth under cold stress varies with strain and molecular subtype. Presented are graphs showing relative area under the curve (AUC) comparison of WT strains and their respective csp deletion mutants at 8oC in BHI during at day 5 of incubation. ∗ Indicates statistically significant difference between the mutant and its respective WT strain (P < 0.05).
(E)
(F)

## Slide 5
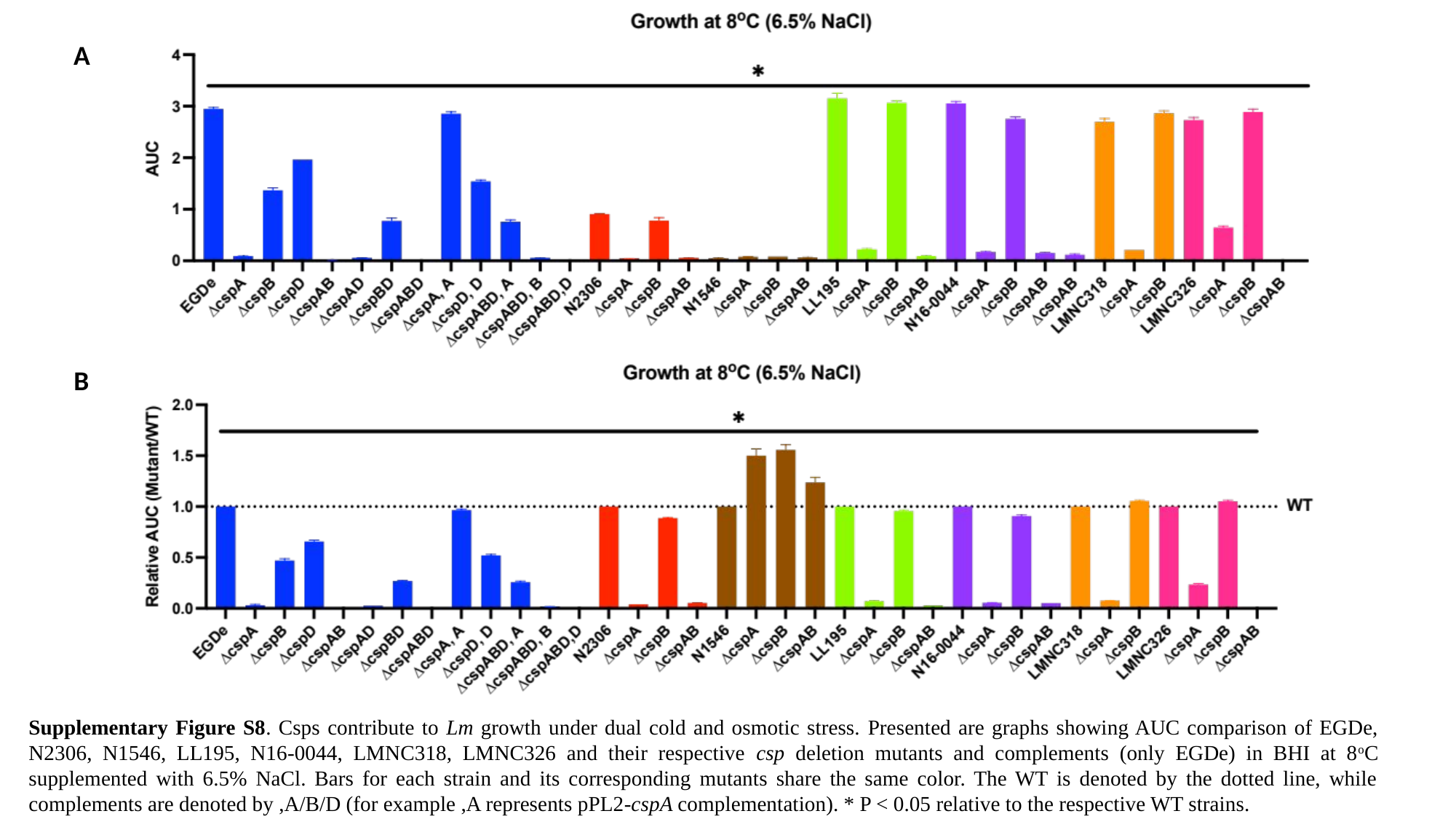

A
B
Supplementary Figure S8. Csps contribute to Lm growth under dual cold and osmotic stress. Presented are graphs showing AUC comparison of EGDe, N2306, N1546, LL195, N16-0044, LMNC318, LMNC326 and their respective csp deletion mutants and complements (only EGDe) in BHI at 8oC supplemented with 6.5% NaCl. Bars for each strain and its corresponding mutants share the same color. The WT is denoted by the dotted line, while complements are denoted by ,A/B/D (for example ,A represents pPL2-cspA complementation). * P < 0.05 relative to the respective WT strains.

## Slide 6
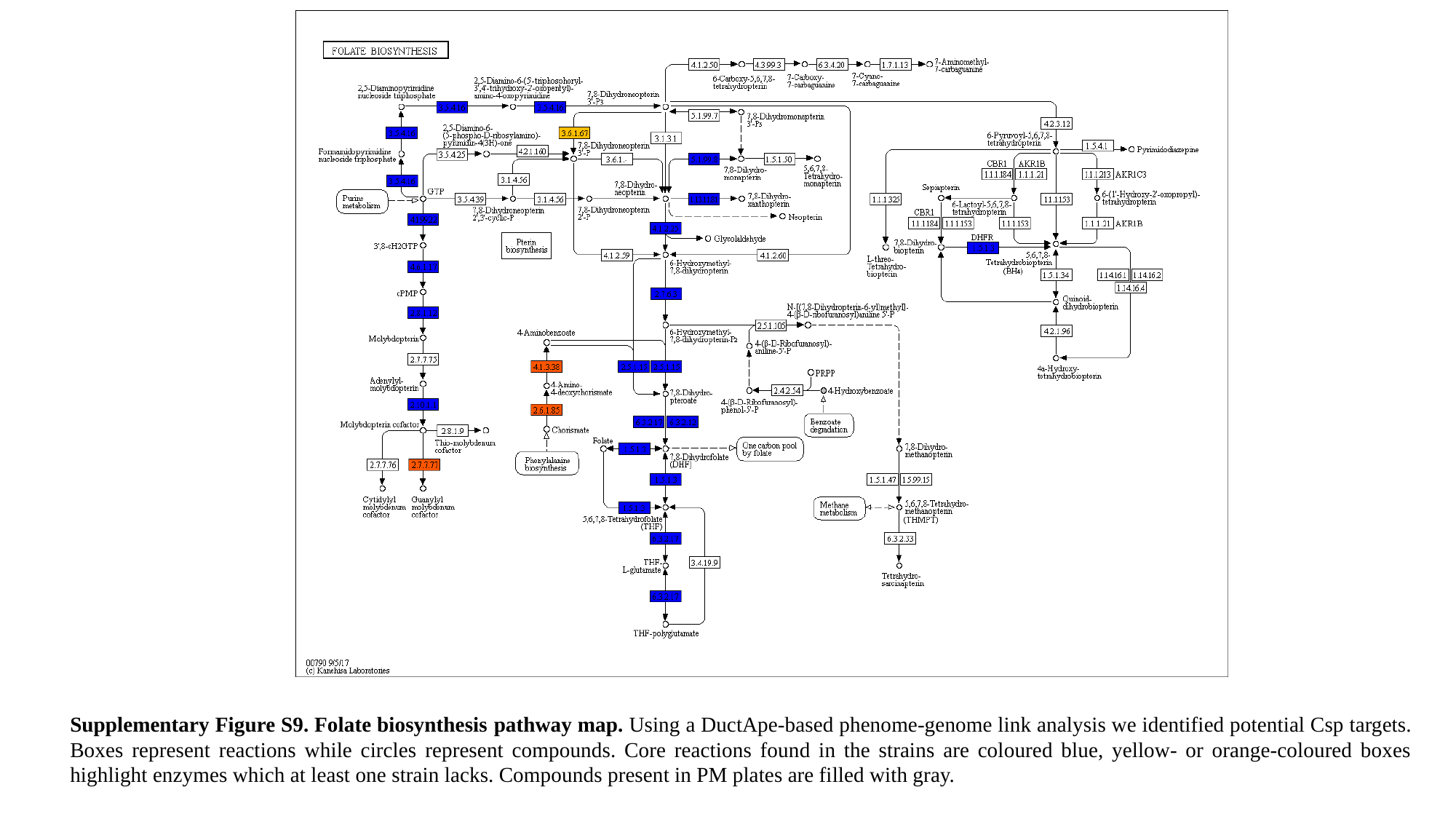

Supplementary Figure S9. Folate biosynthesis pathway map. Using a DuctApe-based phenome-genome link analysis we identified potential Csp targets. Boxes represent reactions while circles represent compounds. Core reactions found in the strains are coloured blue, yellow- or orange-coloured boxes highlight enzymes which at least one strain lacks. Compounds present in PM plates are filled with gray.
