# Supplementary material for: Deciphering the global roles of Cold shock proteins in Listeria monocytogenes nutrient metabolism and stress tolerance
Source: Front Microbiol. 2022 Dec 20;13:1057754. doi: 10.3389/fmicb.2022.1057754 (PMC9808409; doi:10.3389/fmicb.2022.1057754)
Supplement: Supplementary file 2 [file Data_Sheet_1.PDF]

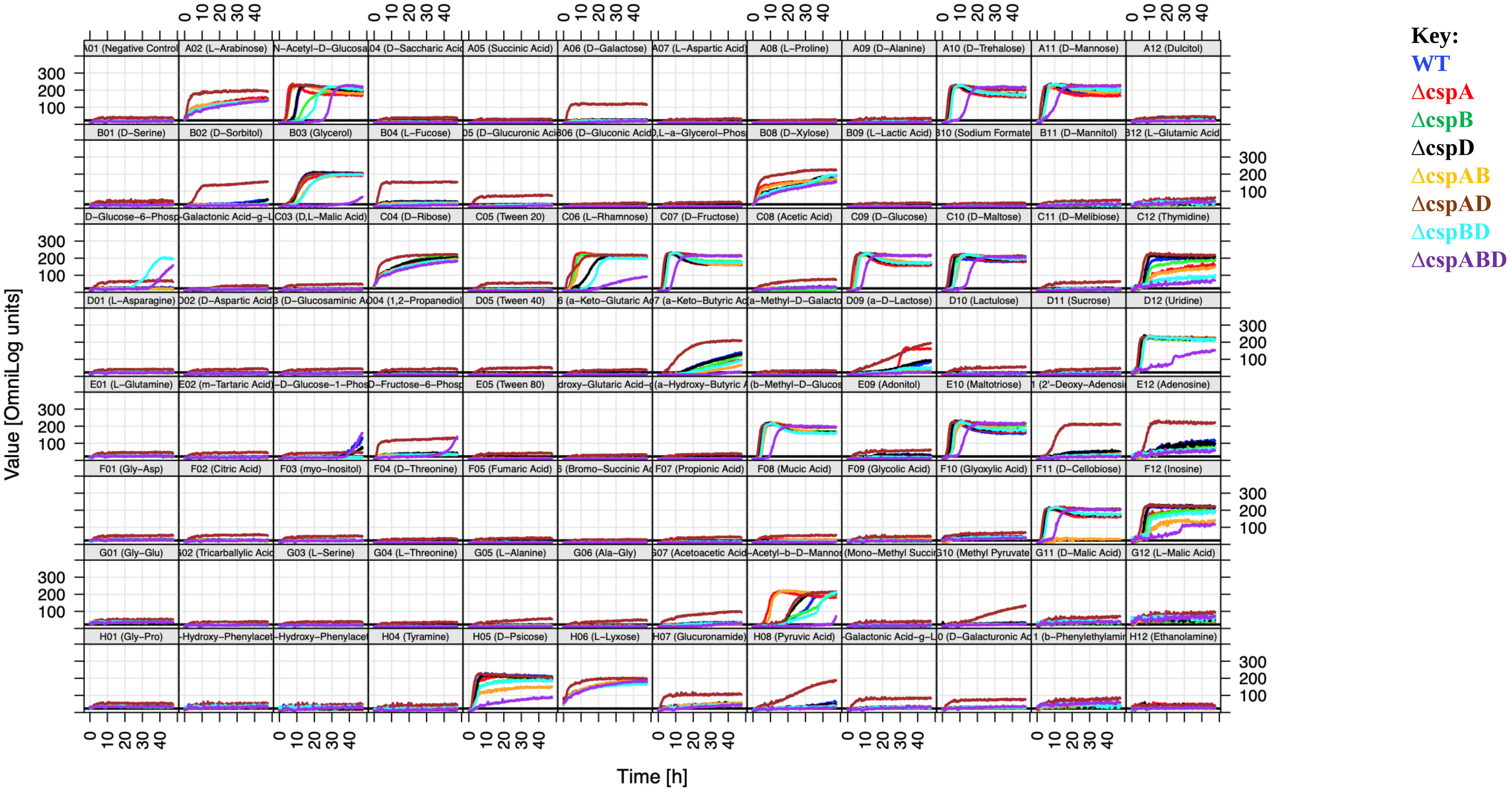

Supplementary Figure S1A: Opm generated graphs for PM01 (C-sources) generated using EGDe and its *csp* deletion mutants.

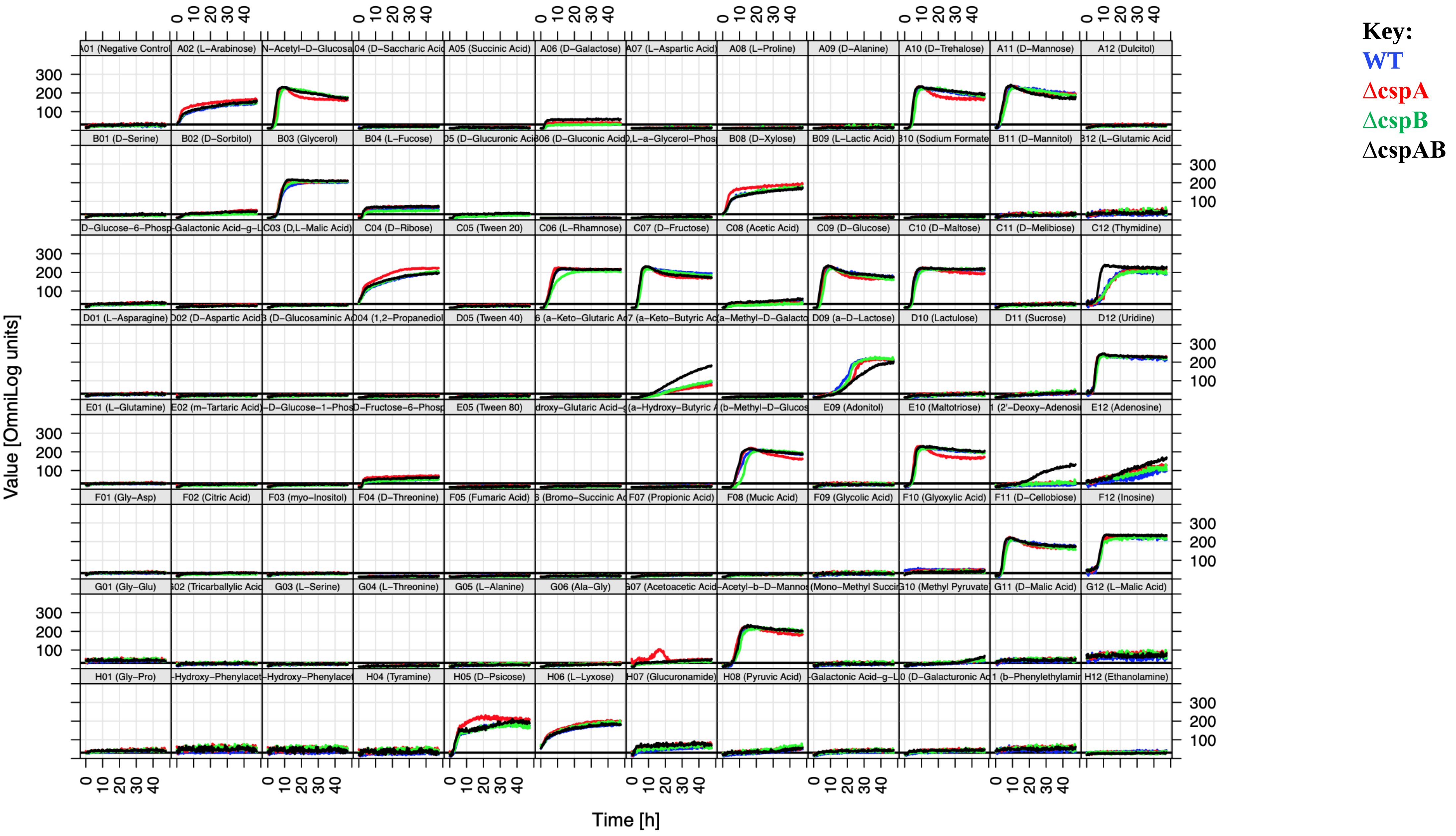

Supplementary Figure S1B: Opm generated graphs for PM01 (C-sources) generated using N1546 and its *csp* deletion mutants.

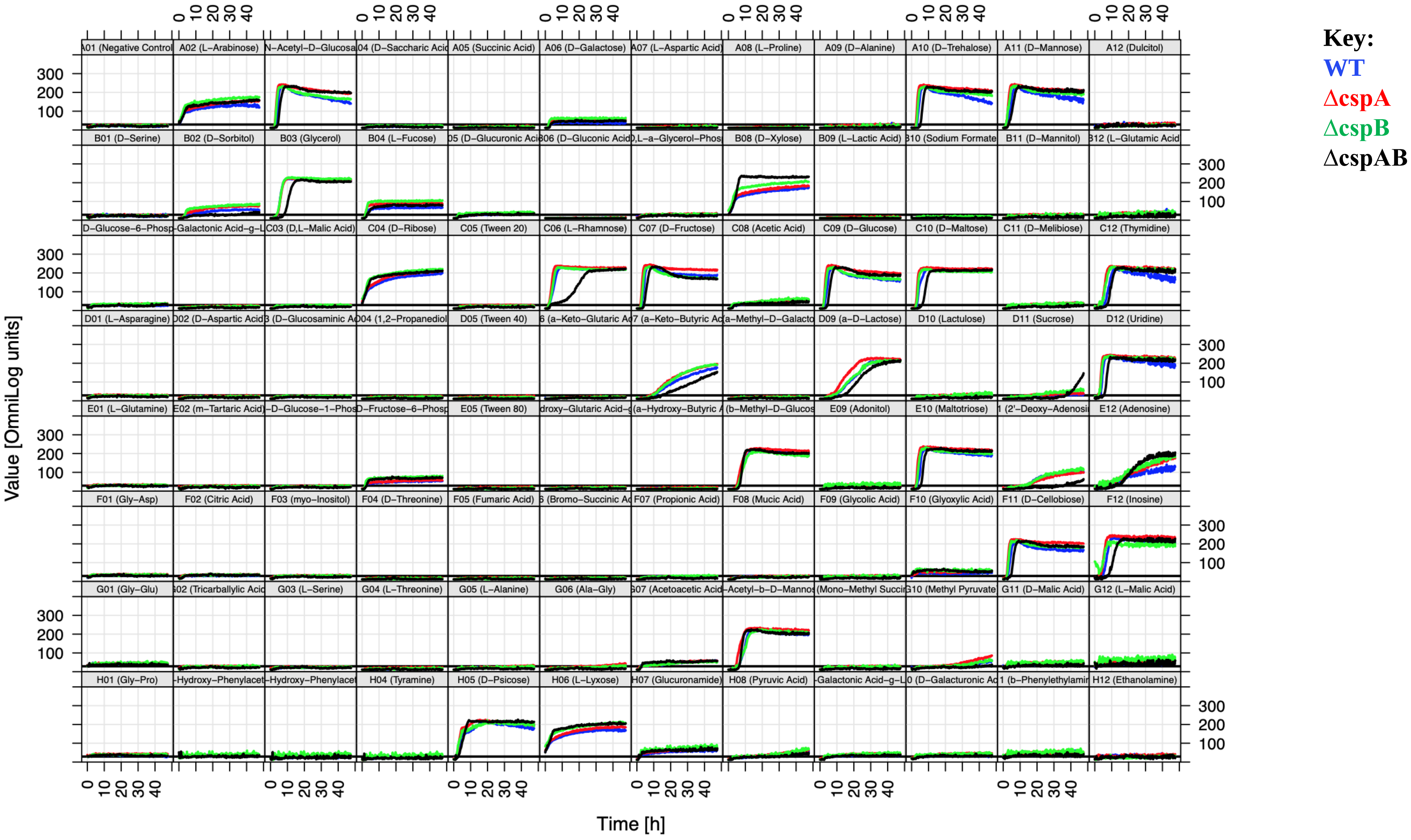

Supplementary Figure S1C: Opm generated graphs for PM01 (C-sources) generated using N2306 and its *csp* deletion mutants.

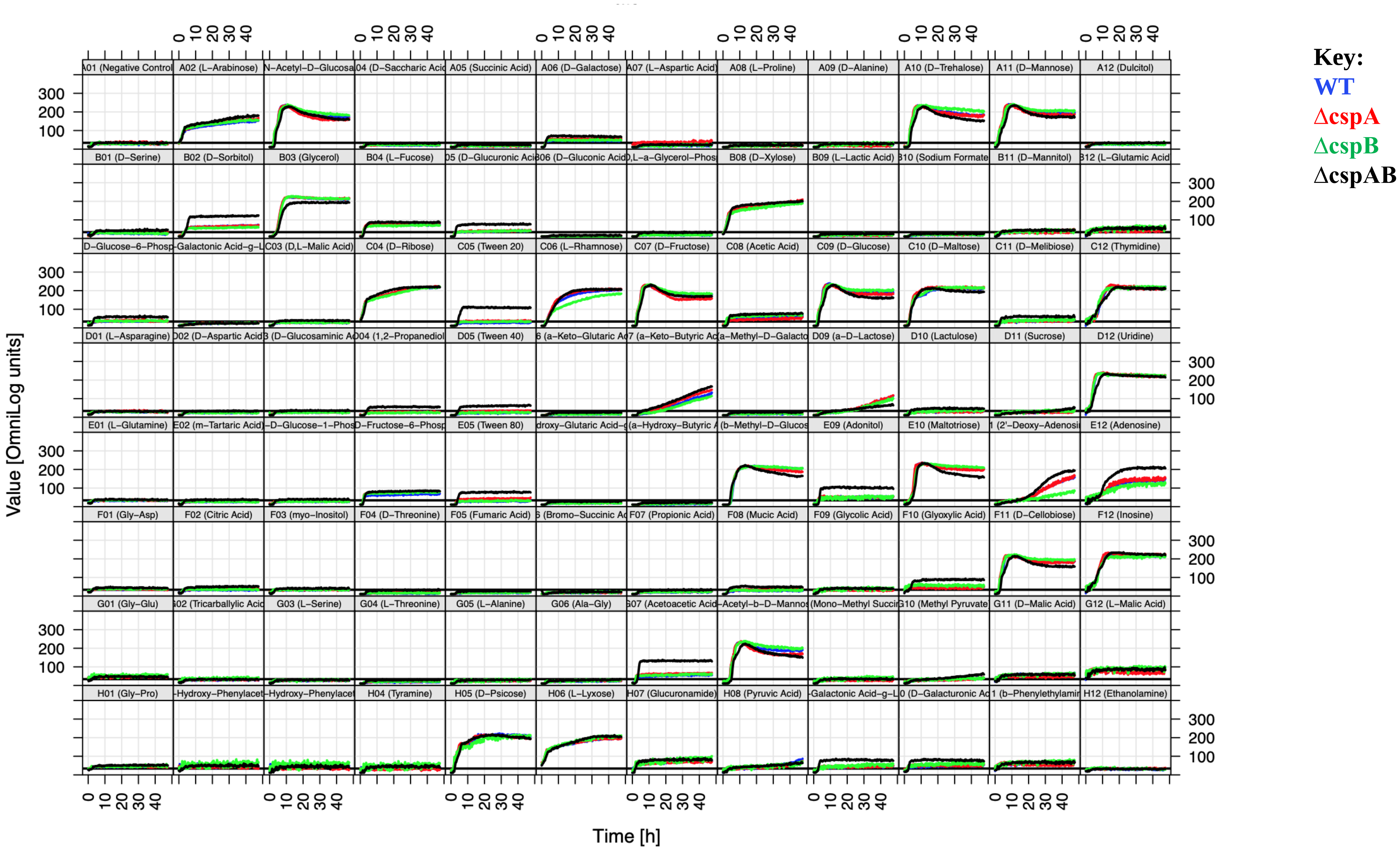

Supplementary Figure S1D: Opm generated graphs for PM01 (C-sources) generated using LL195 and its *csp* deletion mutants.

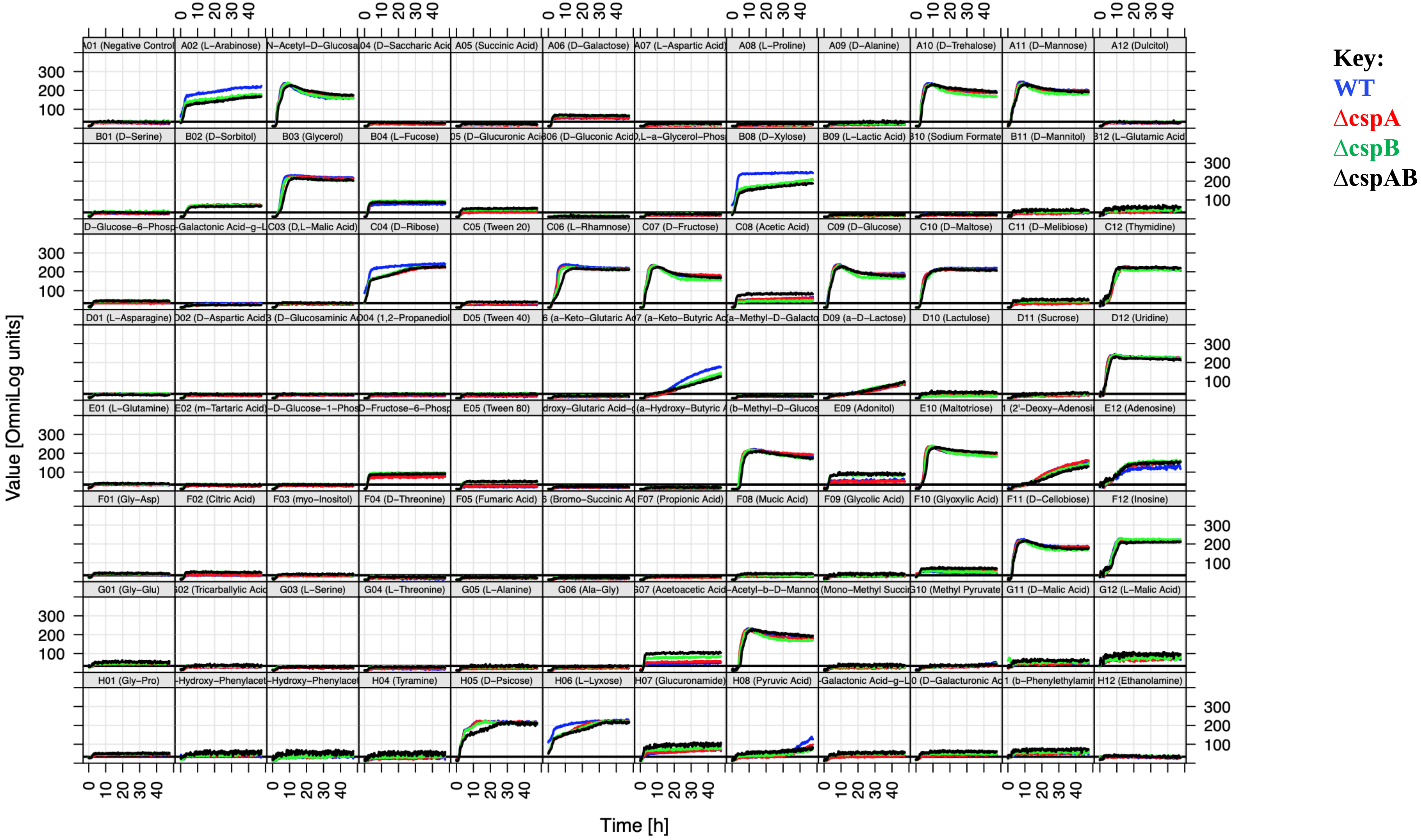

Supplementary Figure S1E: Opm generated graphs for PM01 (C-sources) generated using N16-0044 and its *csp* deletion mutants.

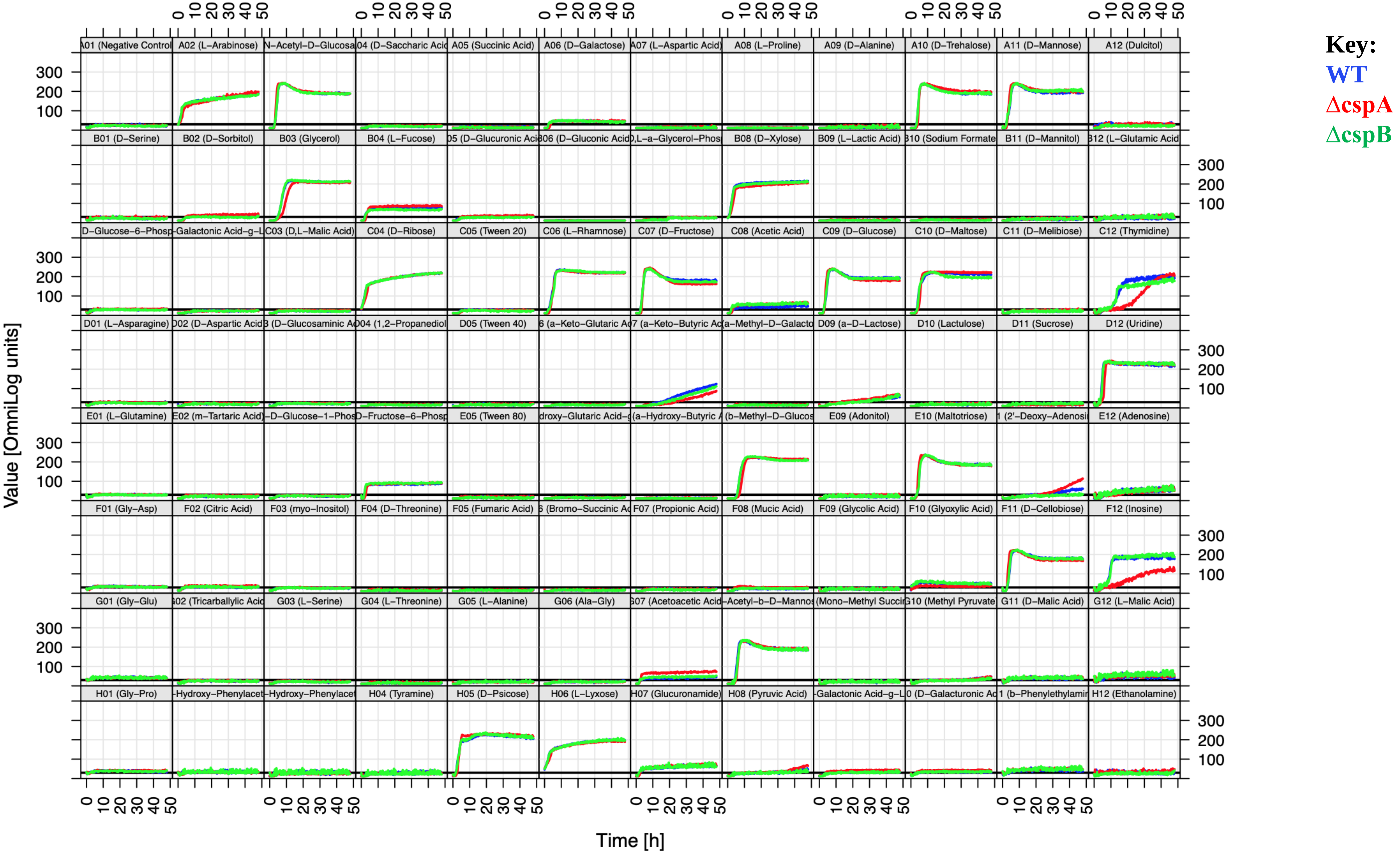

Supplementary Figure S1F: Opm generated graphs for PM01 (C-sources) generated using LMNC318 and its *csp* deletion mutants.

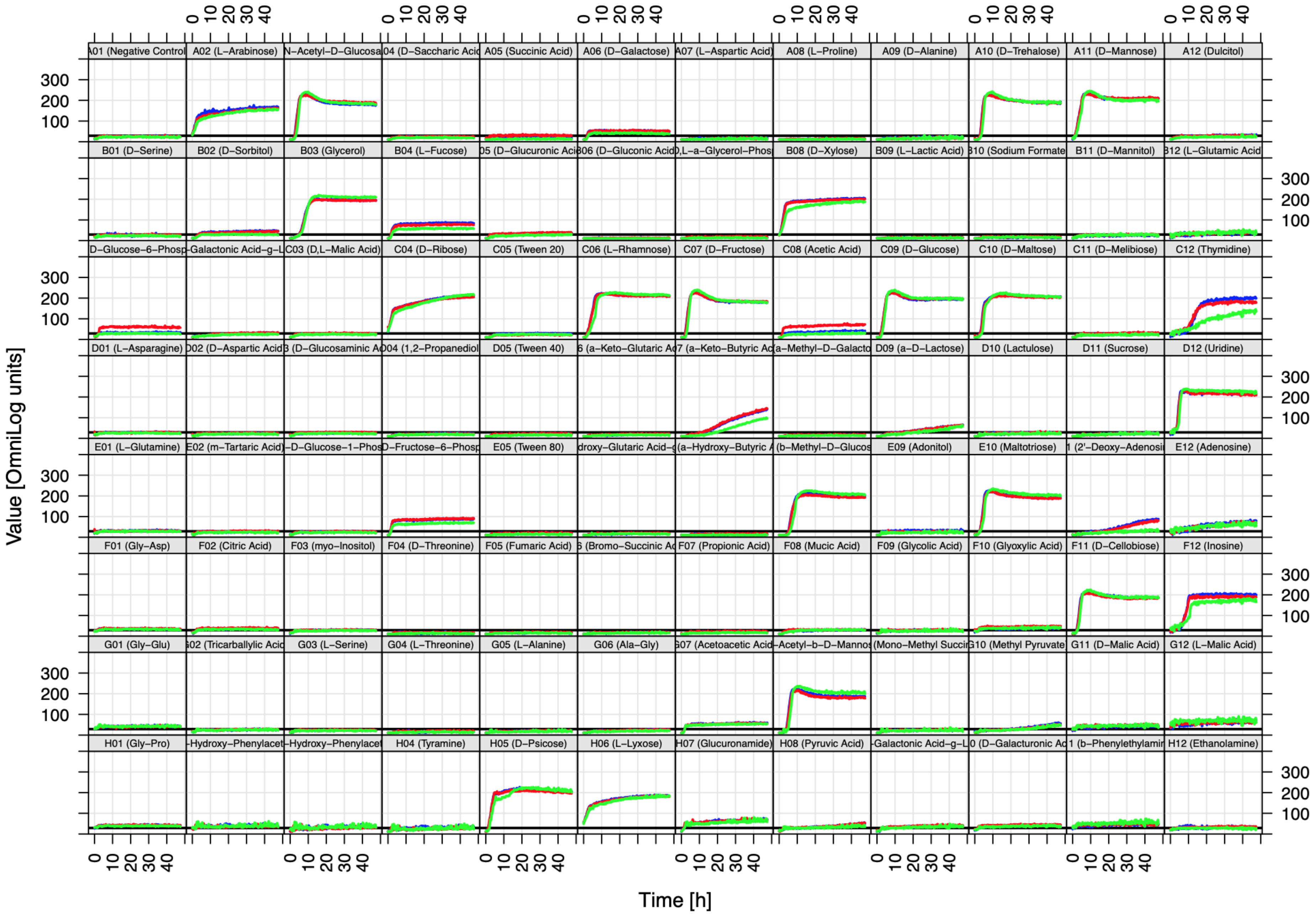

Key:
WT
ΔcspA
ΔcspB

Supplementary Figure S1G: Opm generated graphs for PM01 (C-sources) generated using LMNC326 and its *csp* deletion mutants.

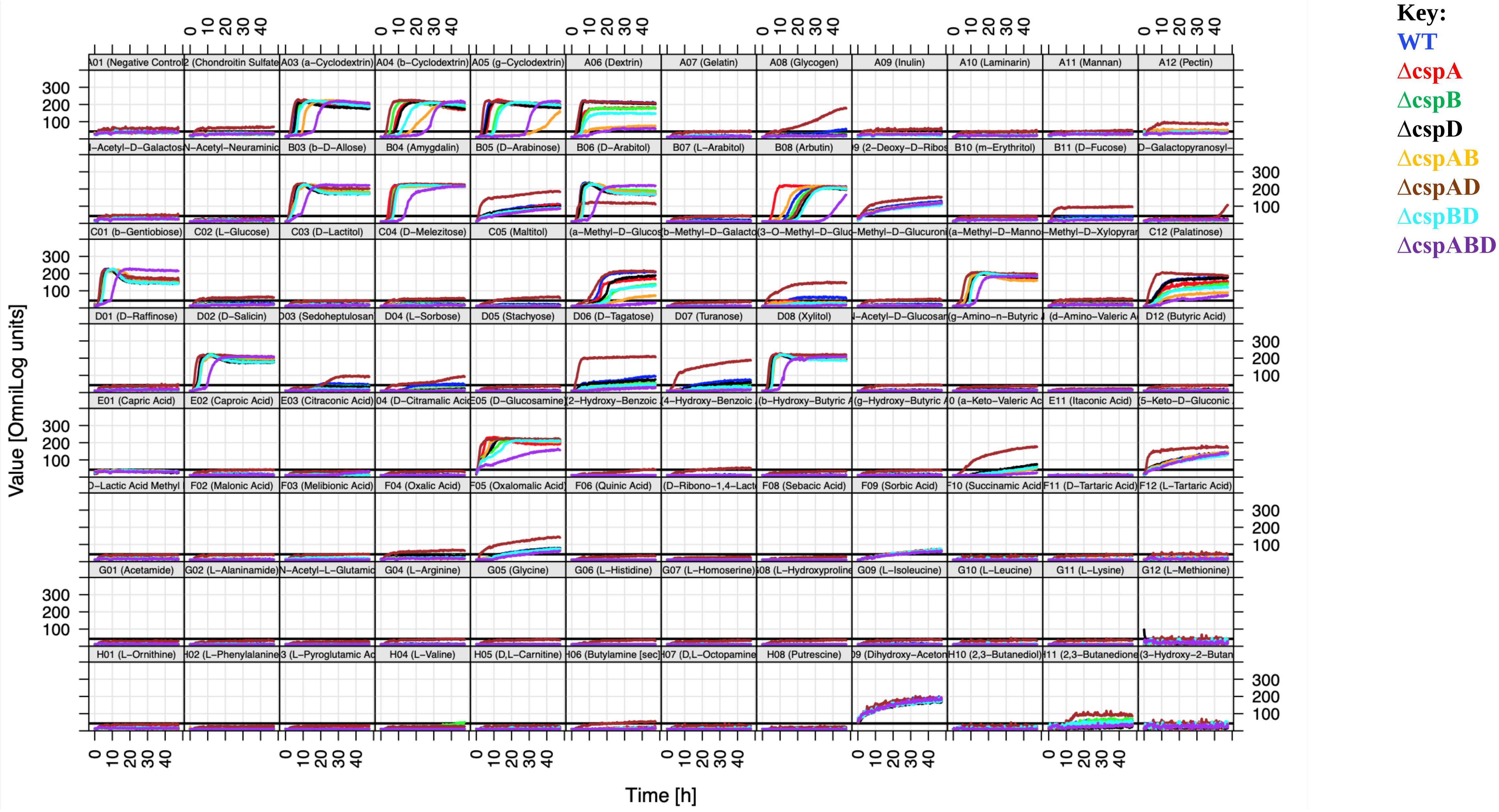

Supplementary Figure S2A: Opm generated graphs for PM02 (C-sources) generated using EGDe and its *csp* deletion mutants

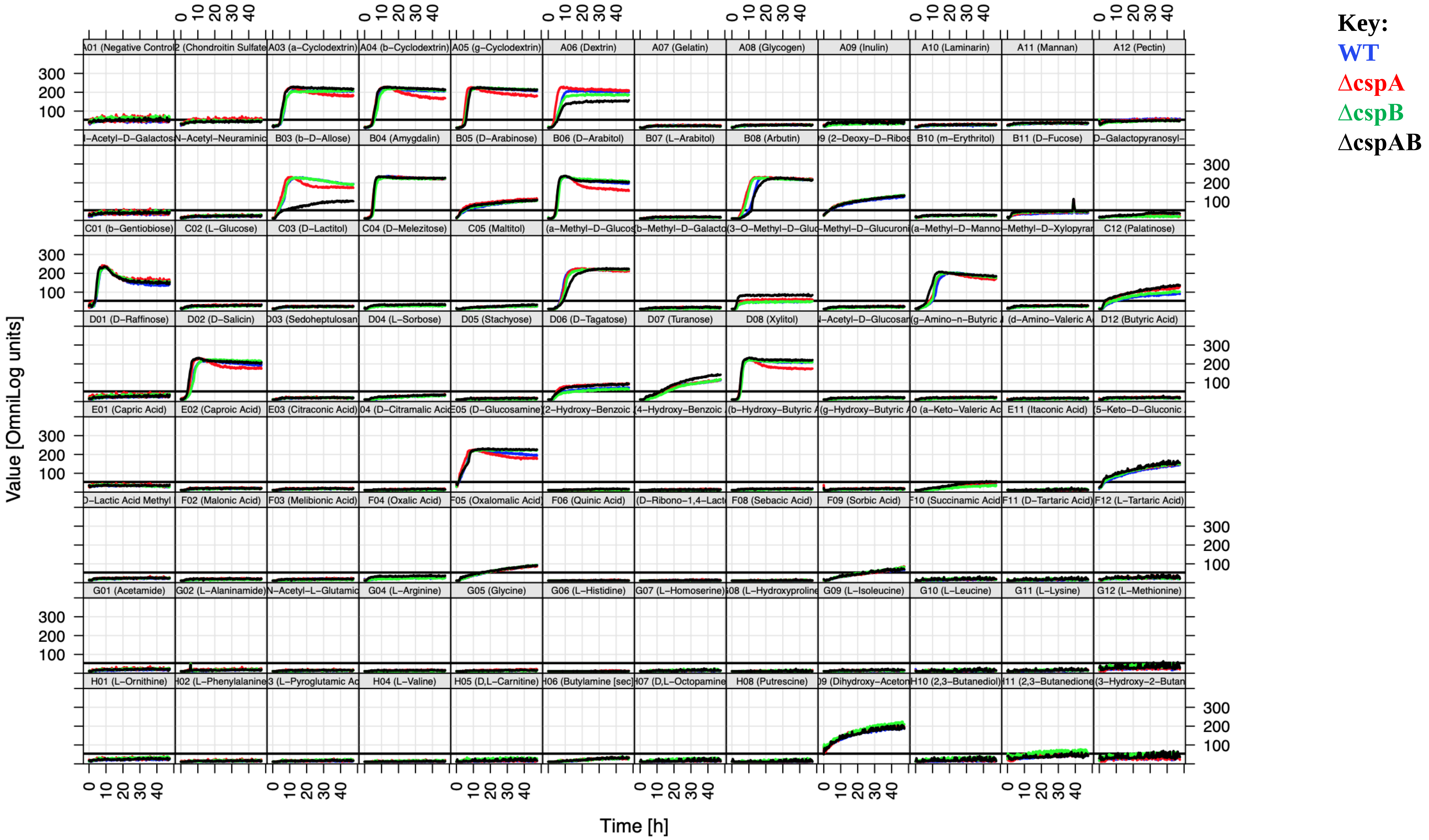

Supplementary Figure S2B: Opm generated graphs for PM02 (C-sources) generated using N1546 and its *csp* deletion mutants

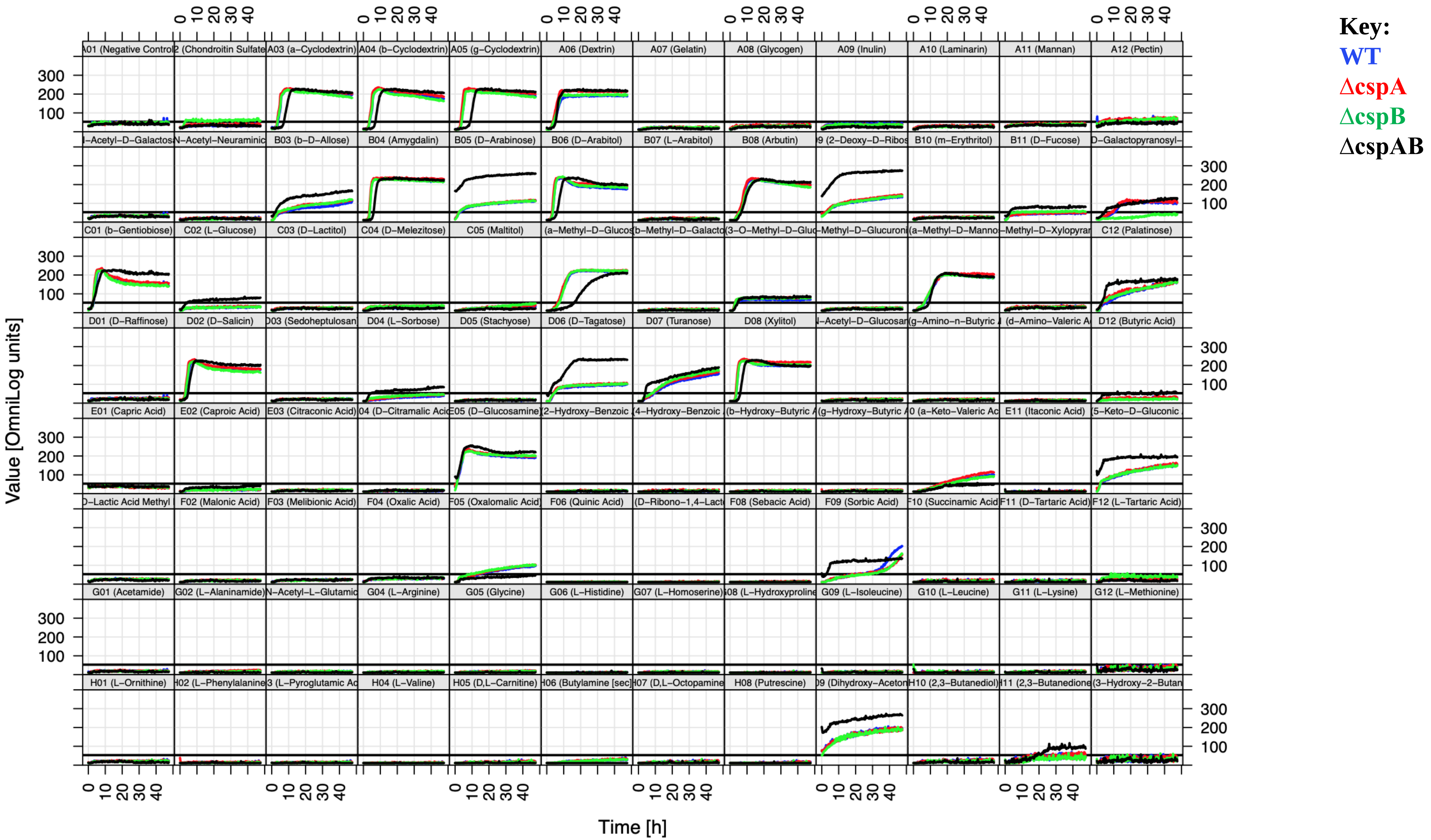

Supplementary Figure S2C: Opm generated graphs for PM02 (C-sources) generated using N2306 and its *csp* deletion mutants

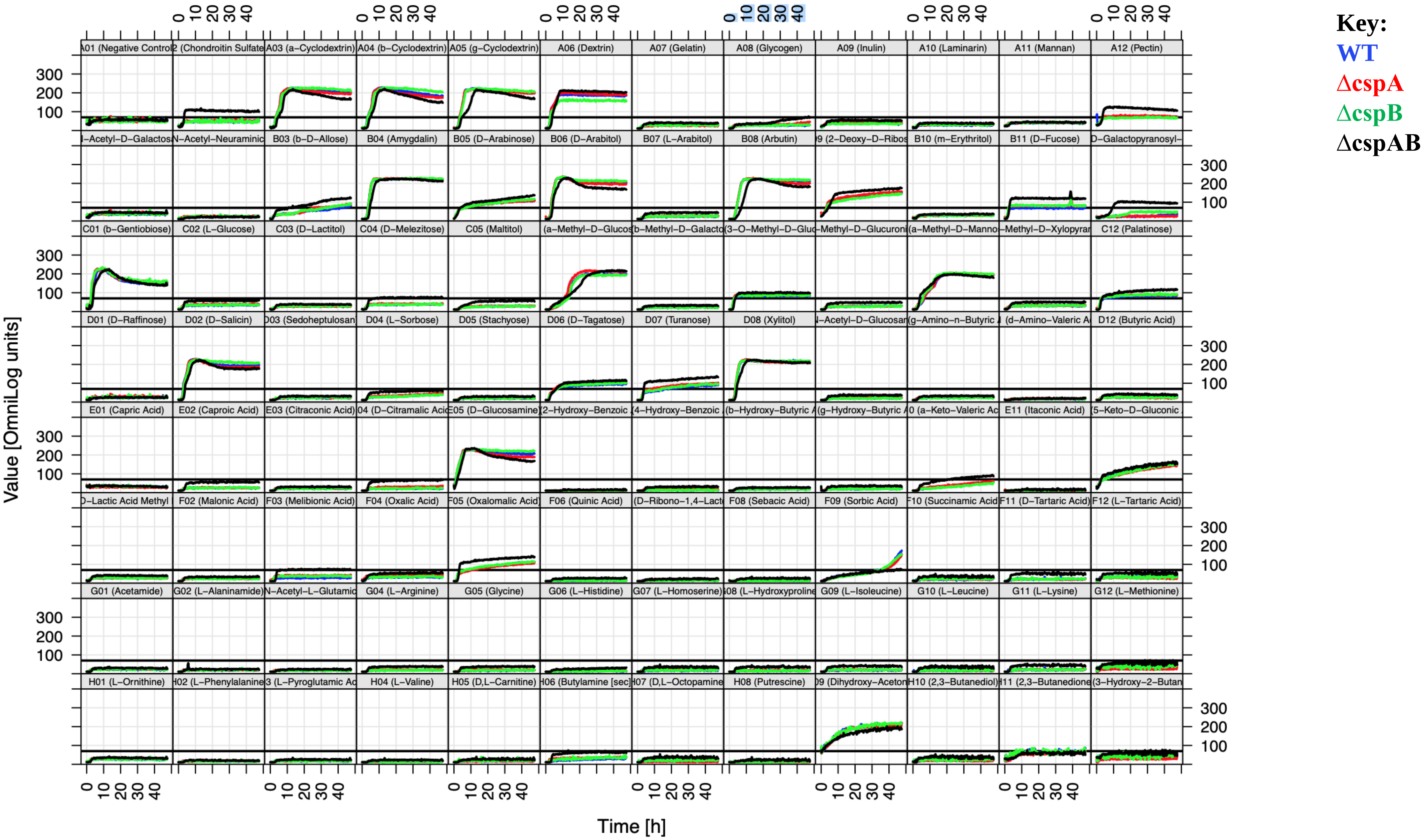

Supplementary Figure S2D: Opm generated graphs for PM02 (C-sources) generated using LL195 and its *csp* deletion mutants

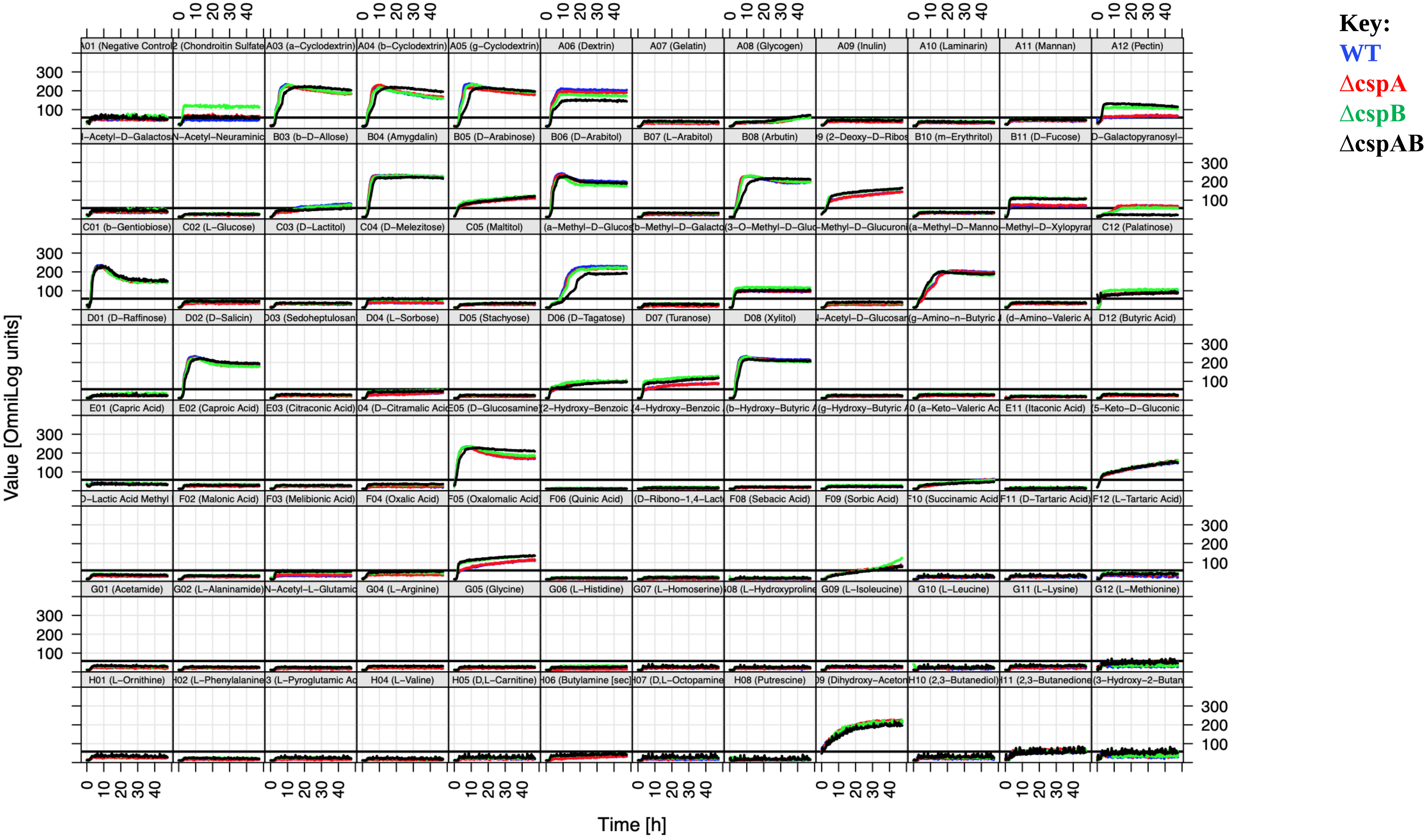

Supplementary Figure S2E: Opm generated graphs for PM02 (C-sources) generated using N16-0044 and its *csp* deletion mutants

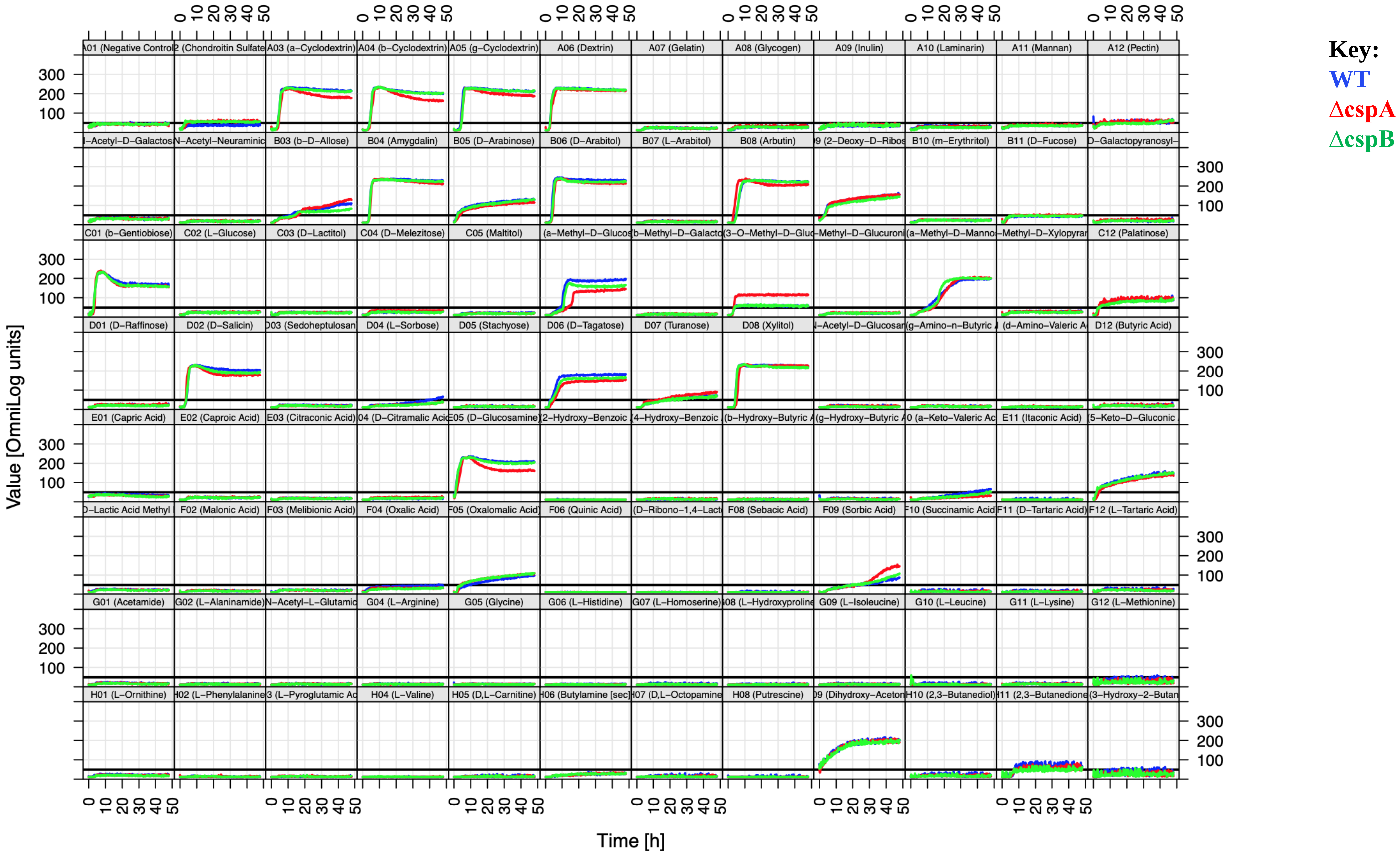

Supplementary Figure S2F: Opm generated graphs for PM02 (C-sources) generated using LMN318 and its *csp* deletion mutants

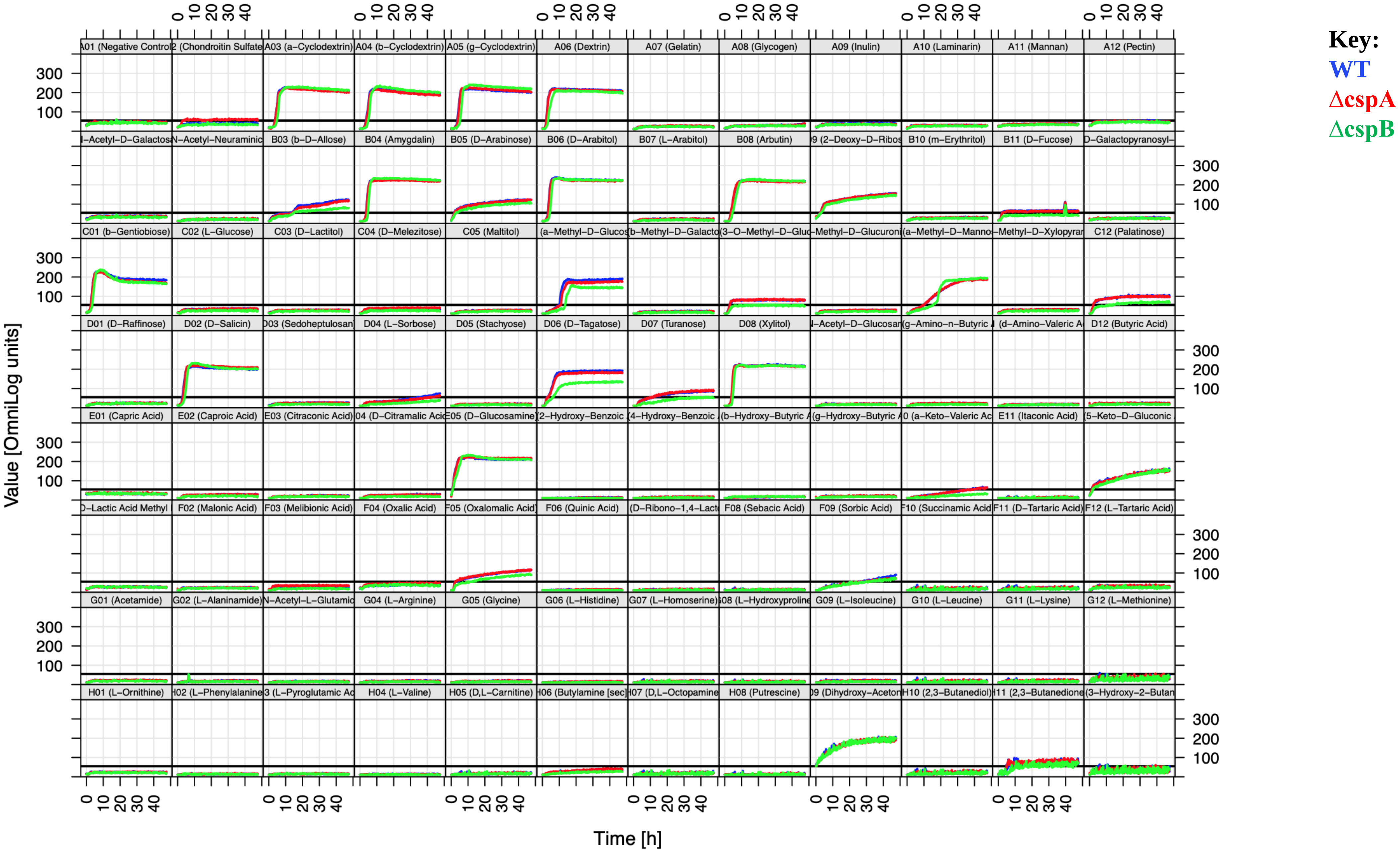

Supplementary Figure S2G: Opm generated graphs for PM02 (C-sources) generated using LMN326 and its *csp* deletion mutants

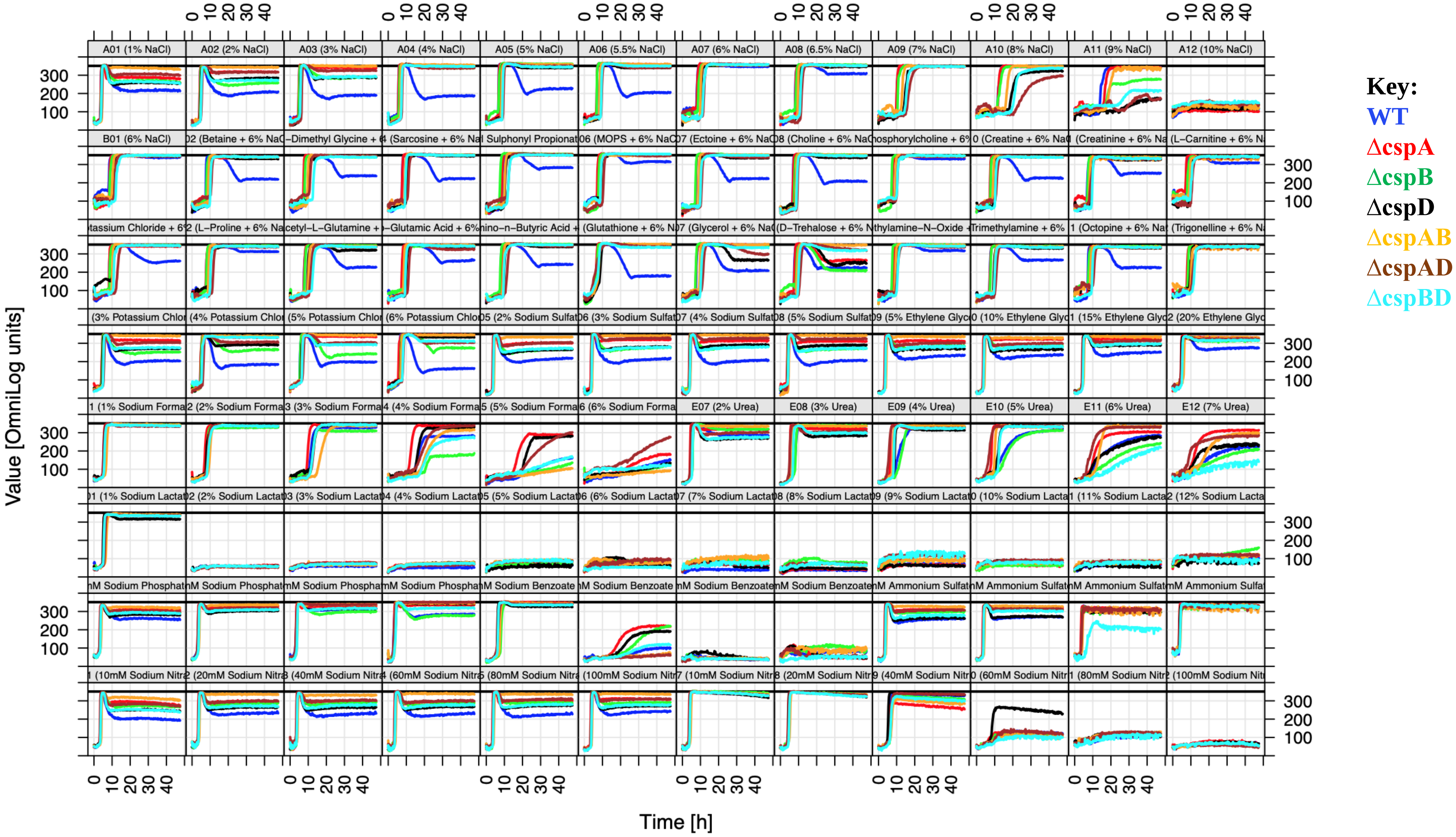

Supplementary Figure S3A: Opm generated graphs for PM09 (osmolytes) generated using EGDe and its *csp* deletion mutants

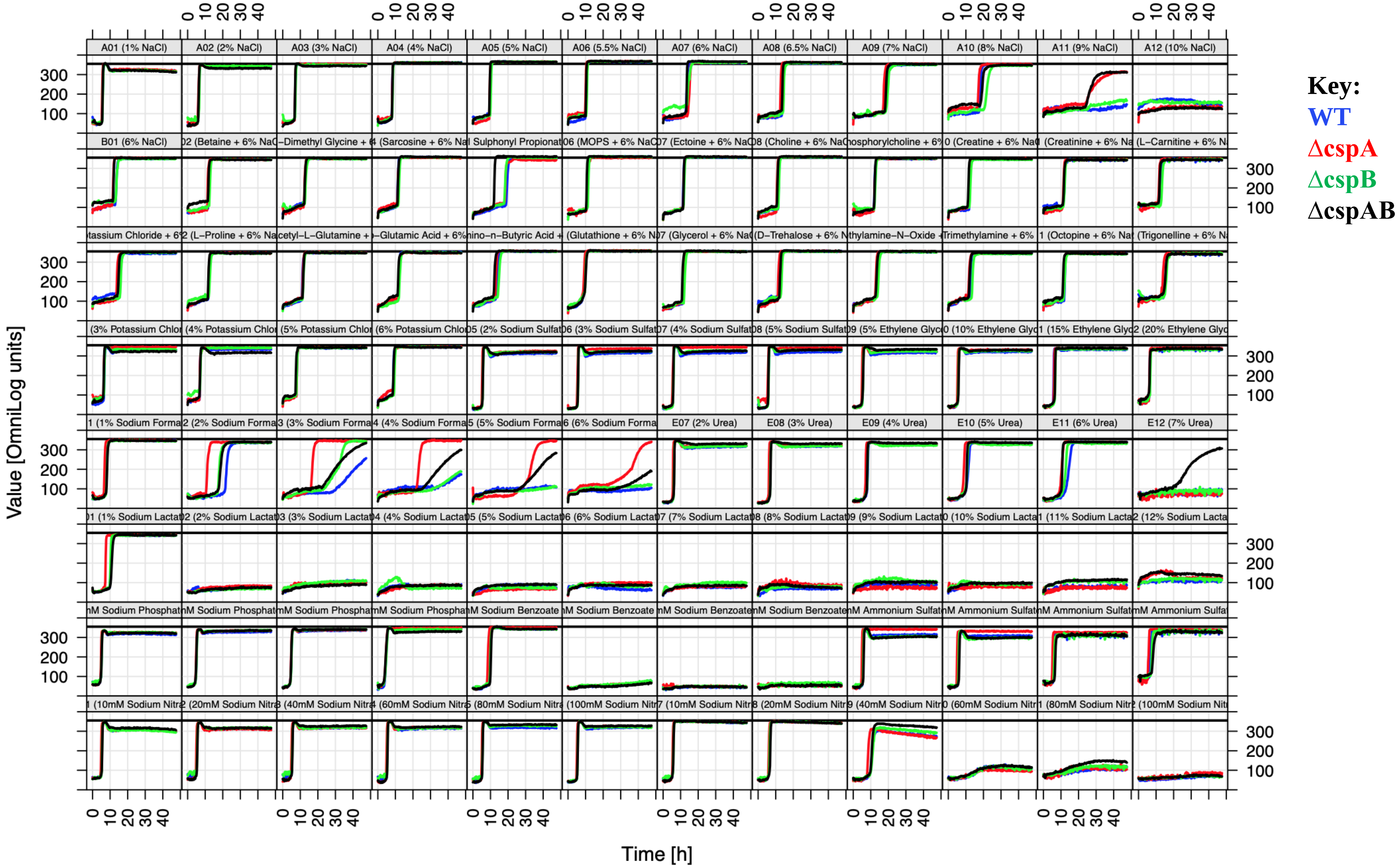

Supplementary Figure S3B: Opm generated graphs for PM09 (osmolytes) generated using N1546 and its *csp* deletion mutants

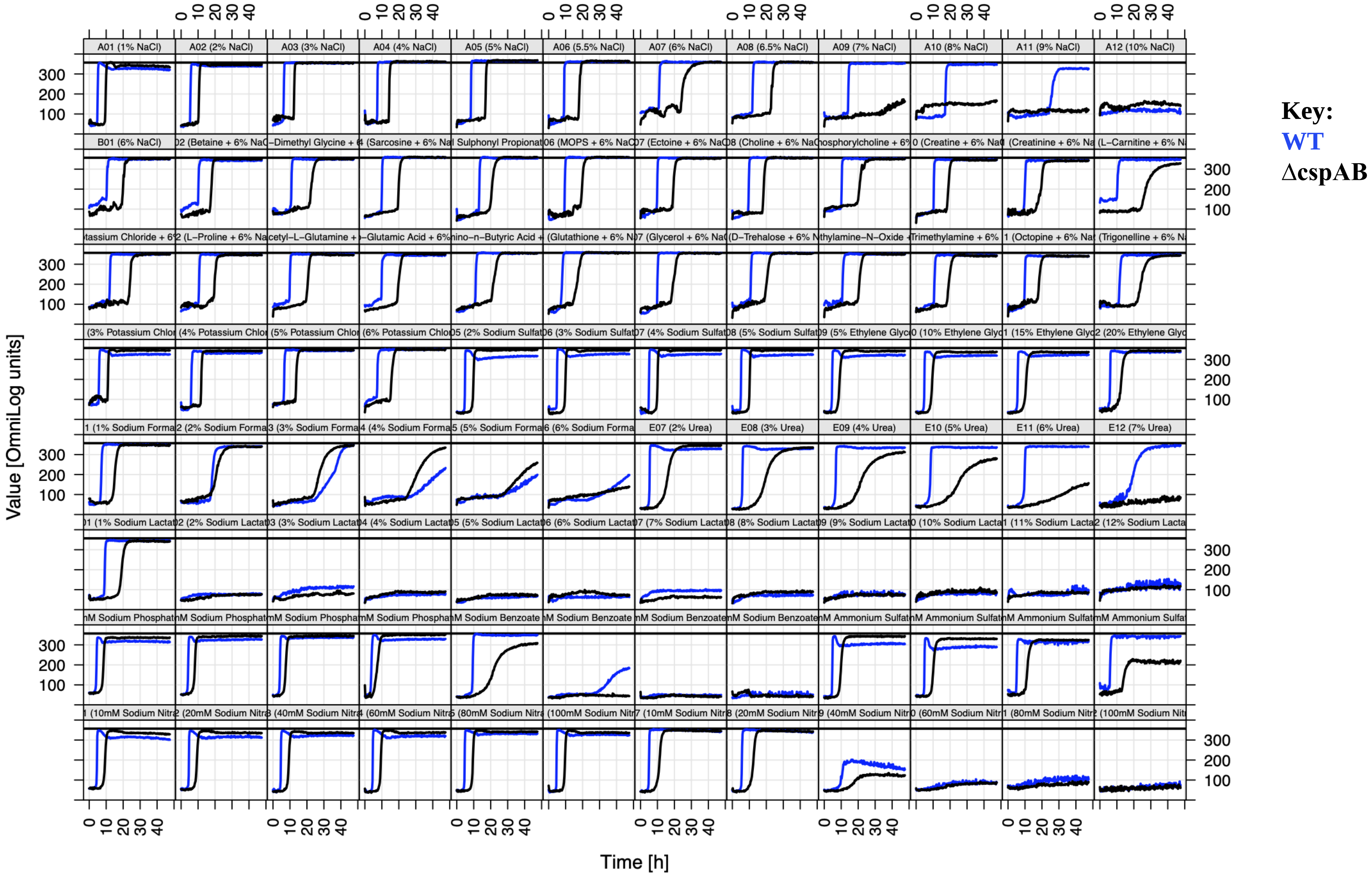

Supplementary Figure S3C: Opm generated graphs for PM09 (osmolytes) generated using N2306 and its *csp* deletion mutants

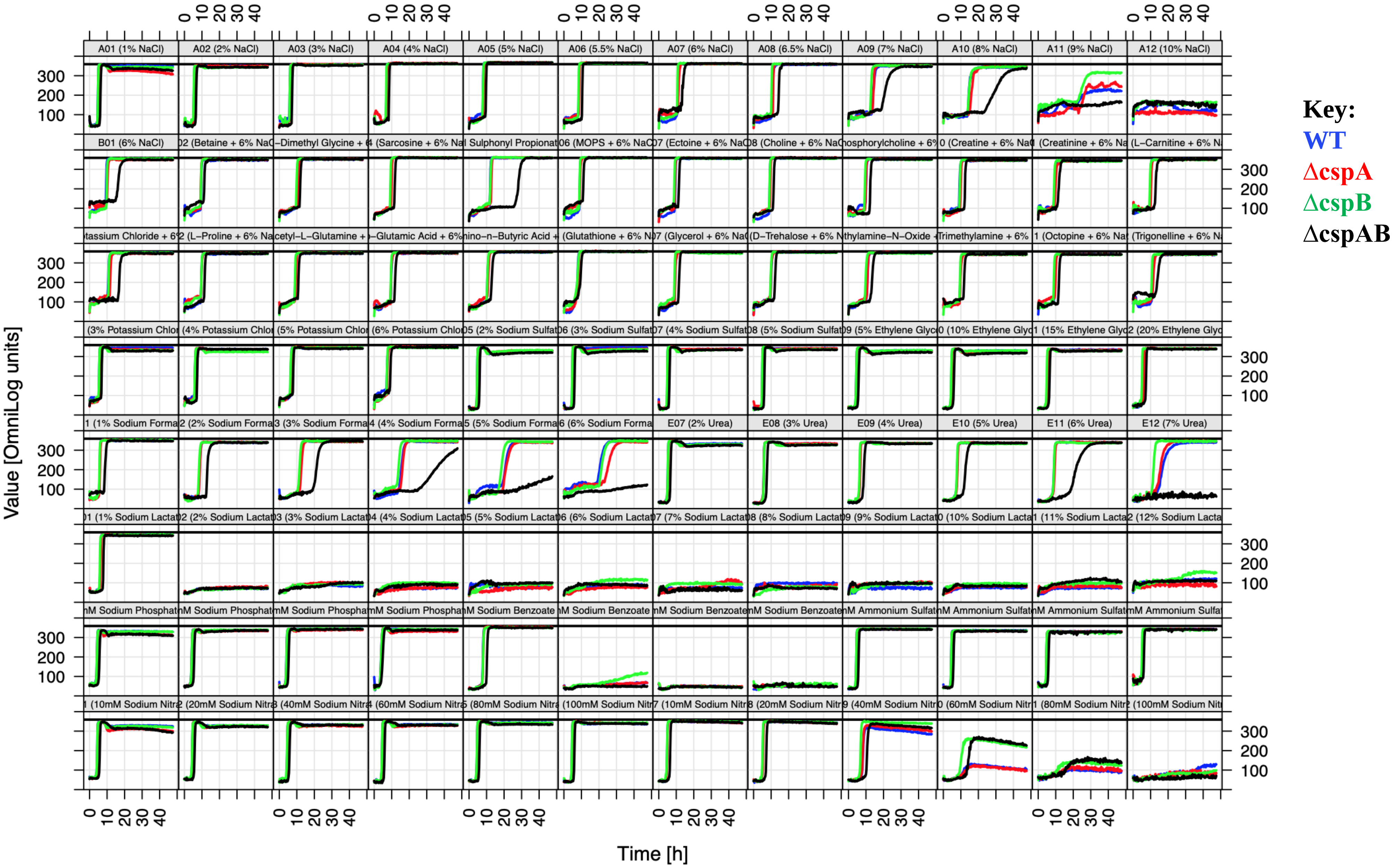

Supplementary Figure S3D: Opm generated graphs for PM09 (osmolytes) generated using LL195 and its *csp* deletion mutants

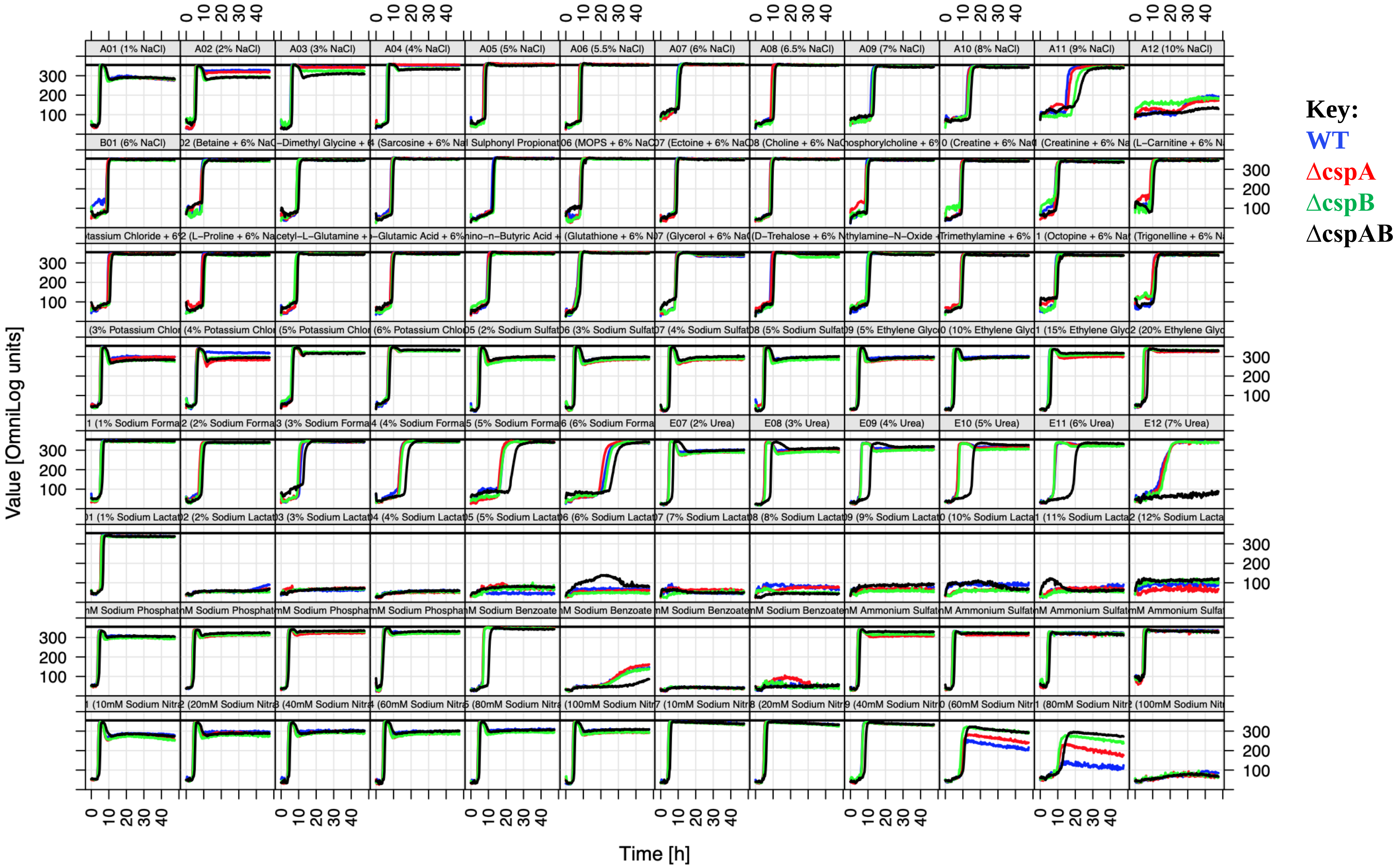

Supplementary Figure S3E: Opm generated graphs for PM09 (osmolytes) generated using N16-0044 and its *csp* deletion mutants

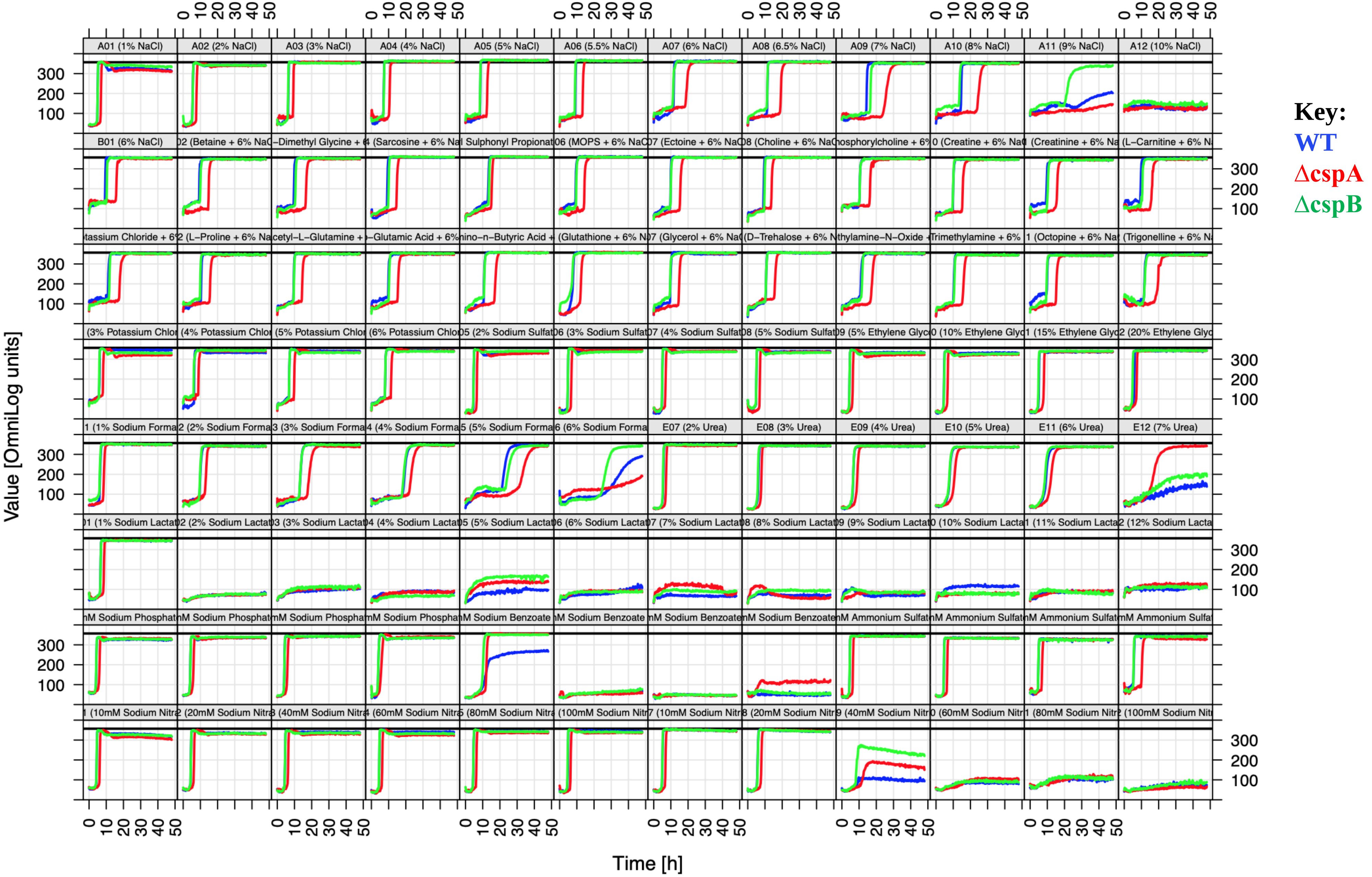

Supplementary Figure S3F: Opm generated graphs for PM09 (osmolytes) generated using LMNC318 and its *csp* deletion mutants

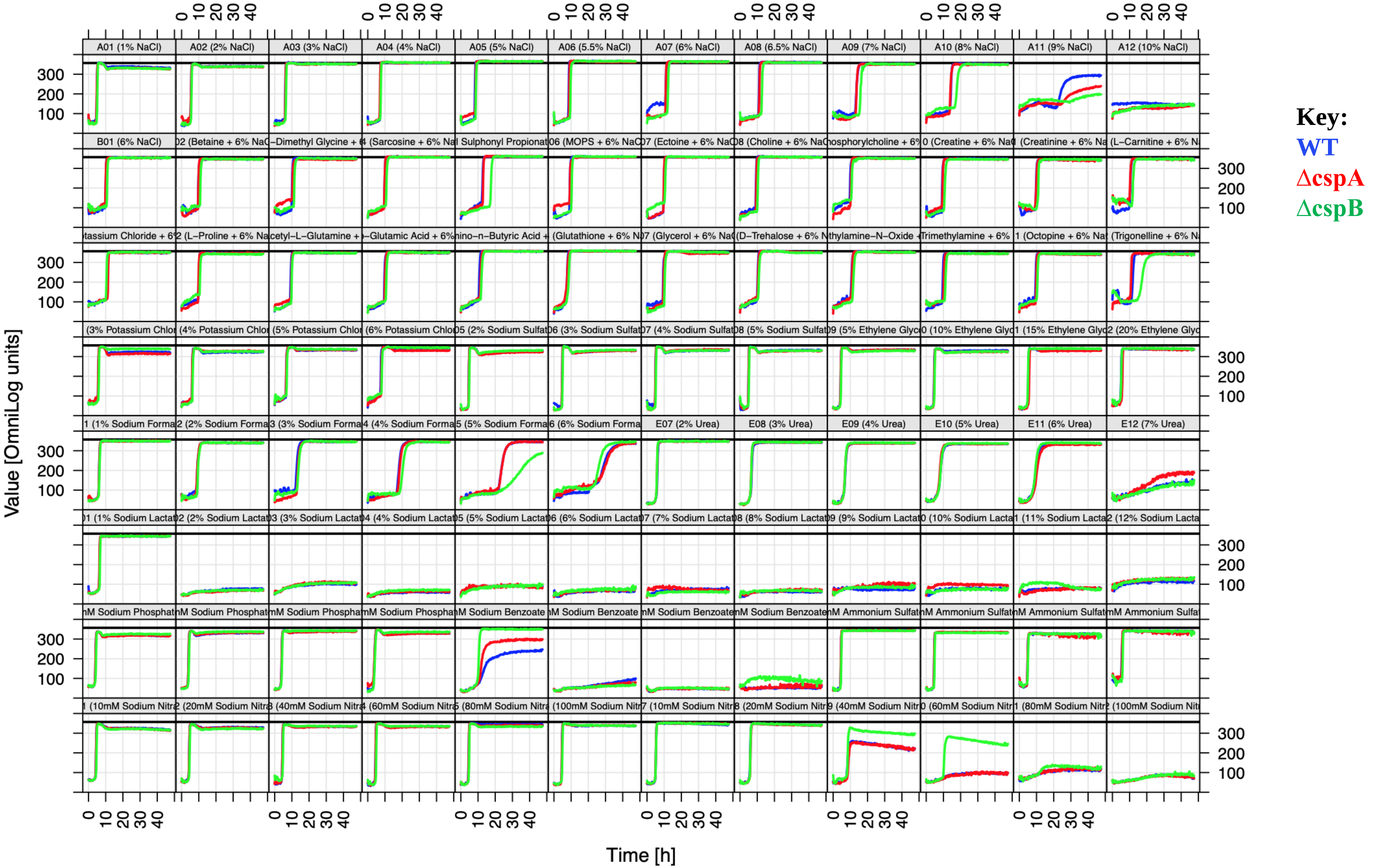

Supplementary Figure S3G: Opm generated graphs for PM09 (osmolytes) generated using LMNC326 and its *csp* deletion mutants

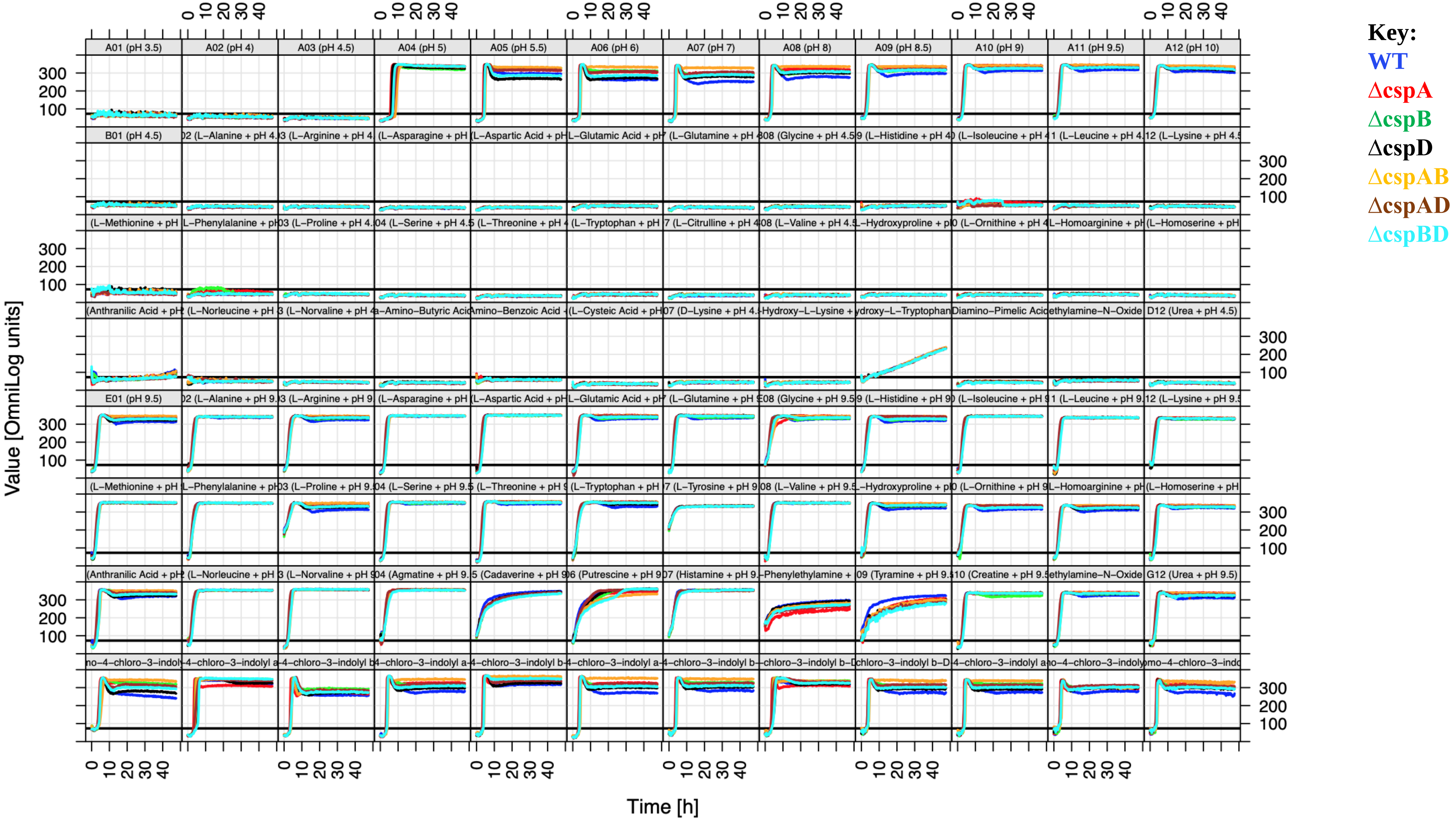

Supplementary Figure S4A: Opm generated graphs for PM10 (pH) generated using EGDe and its *csp* deletion mutants

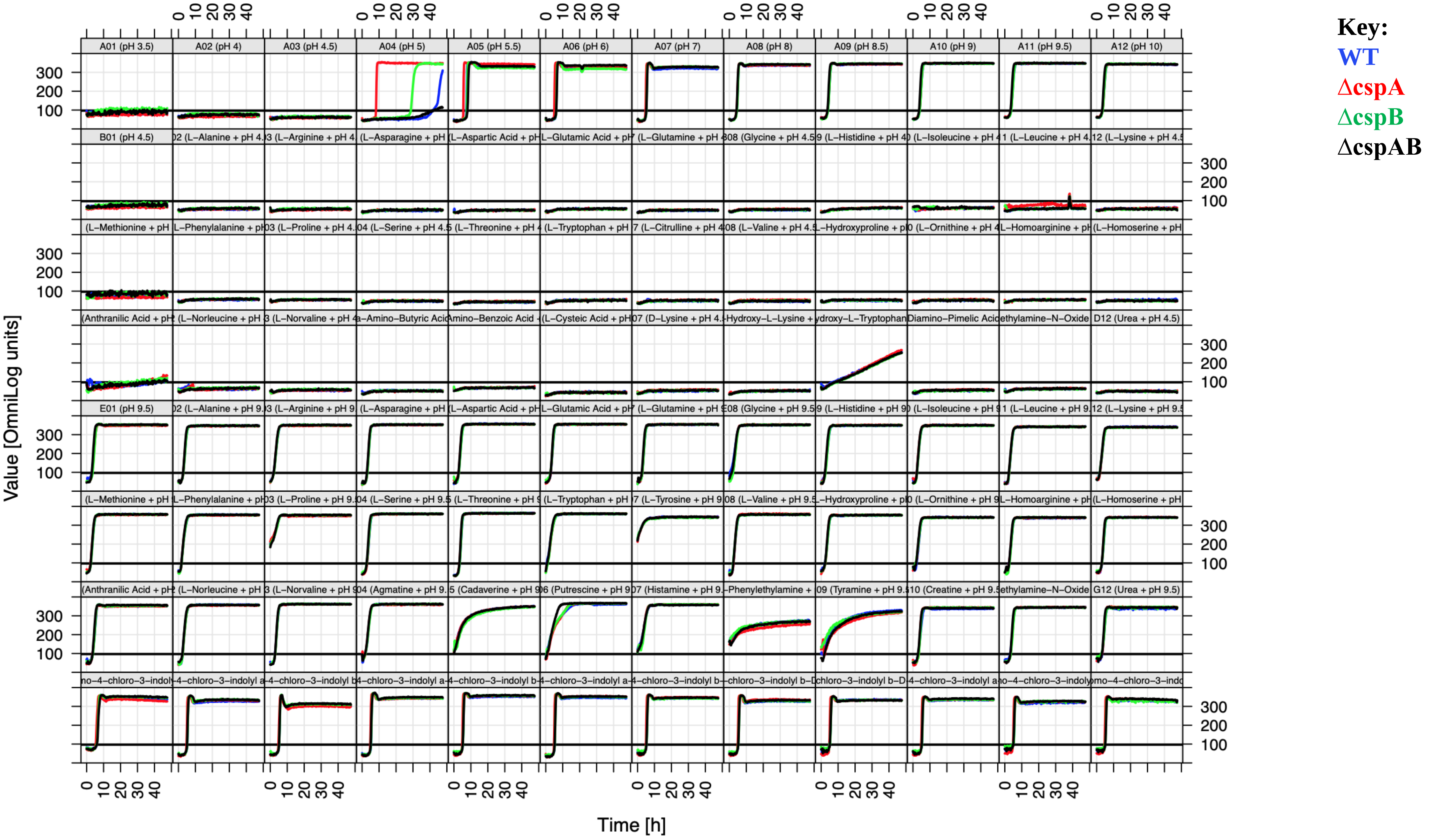

Supplementary Figure S4B: Opm generated graphs for PM10 (pH) generated using N1546 and its *csp* deletion mutants

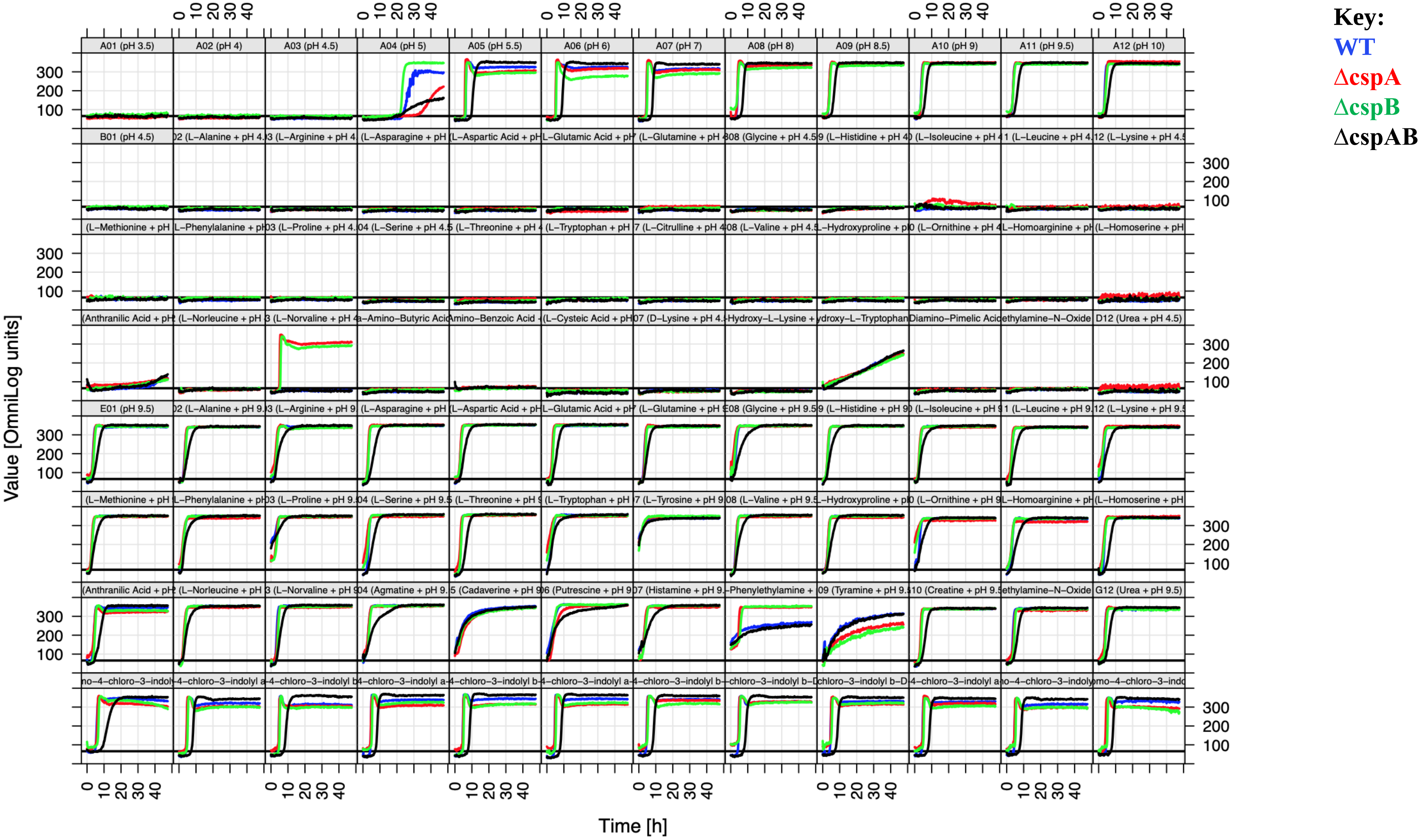

Supplementary Figure S4C: Opm generated graphs for PM10 (pH) generated using N2306 and its *csp* deletion mutants

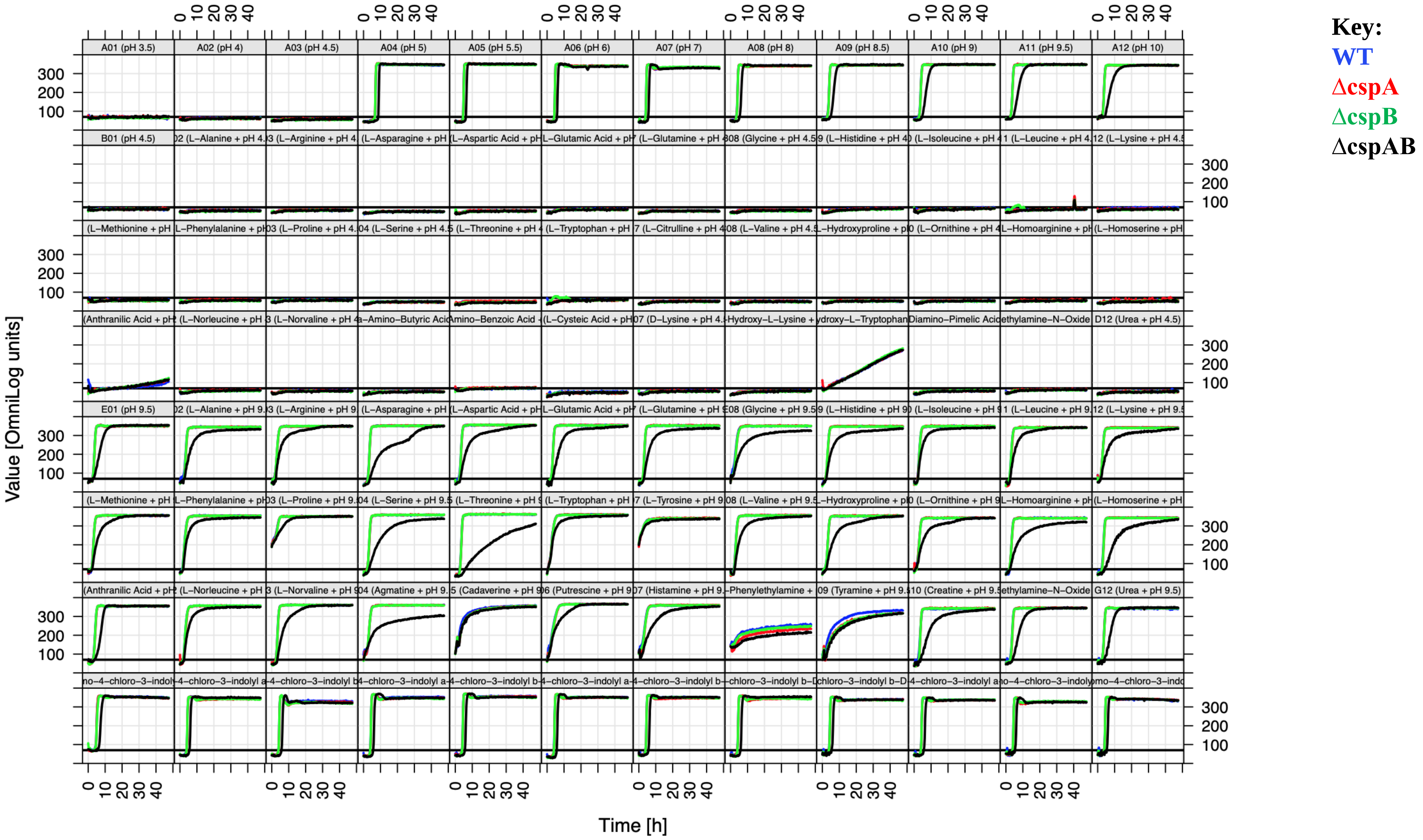

Supplementary Figure S4D: Opm generated graphs for PM10 (pH) generated using LL195 and its *csp* deletion mutants

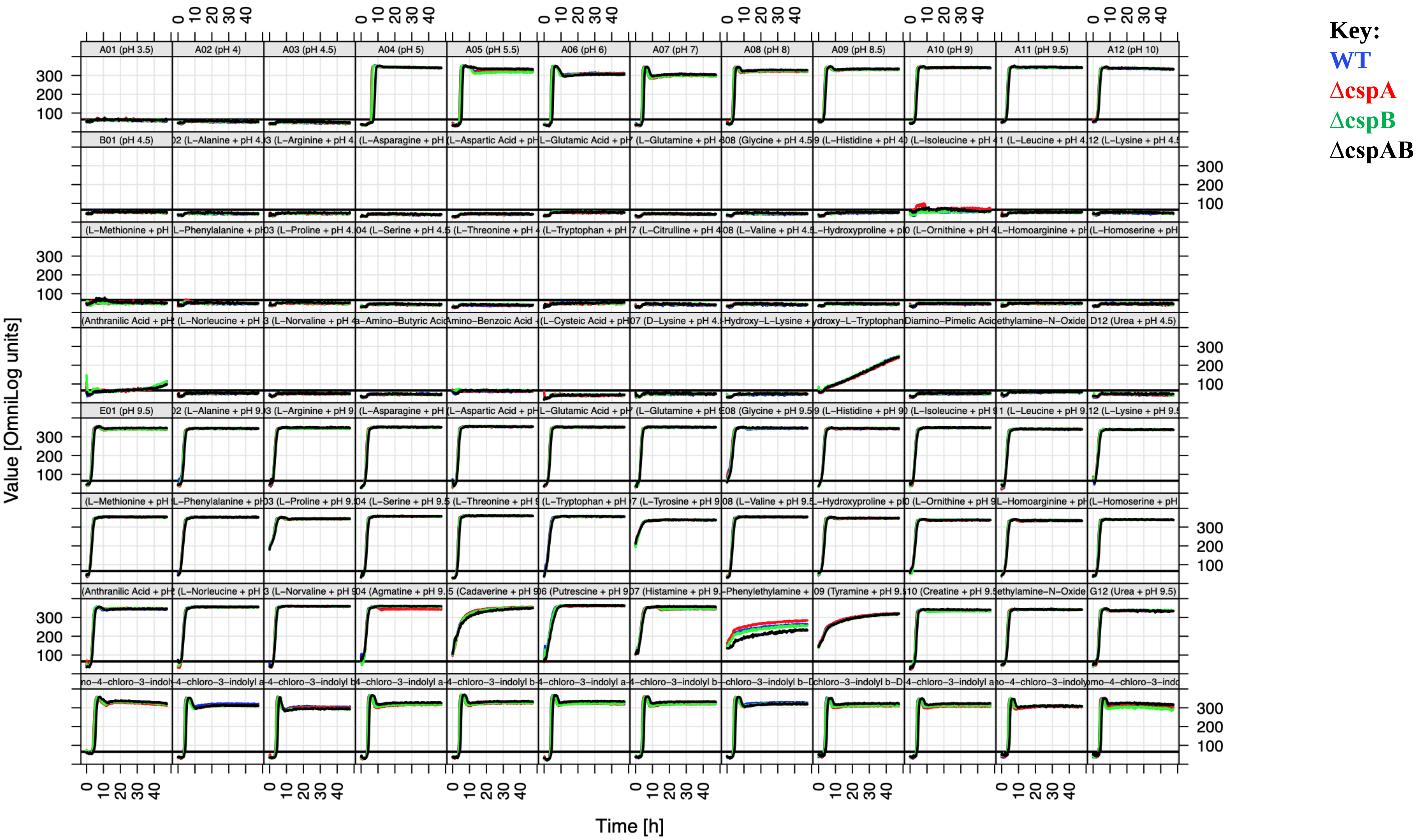

Supplementary Figure S4E: Opm generated graphs for PM10 (pH) generated using N16-0044 and its *csp* deletion mutants

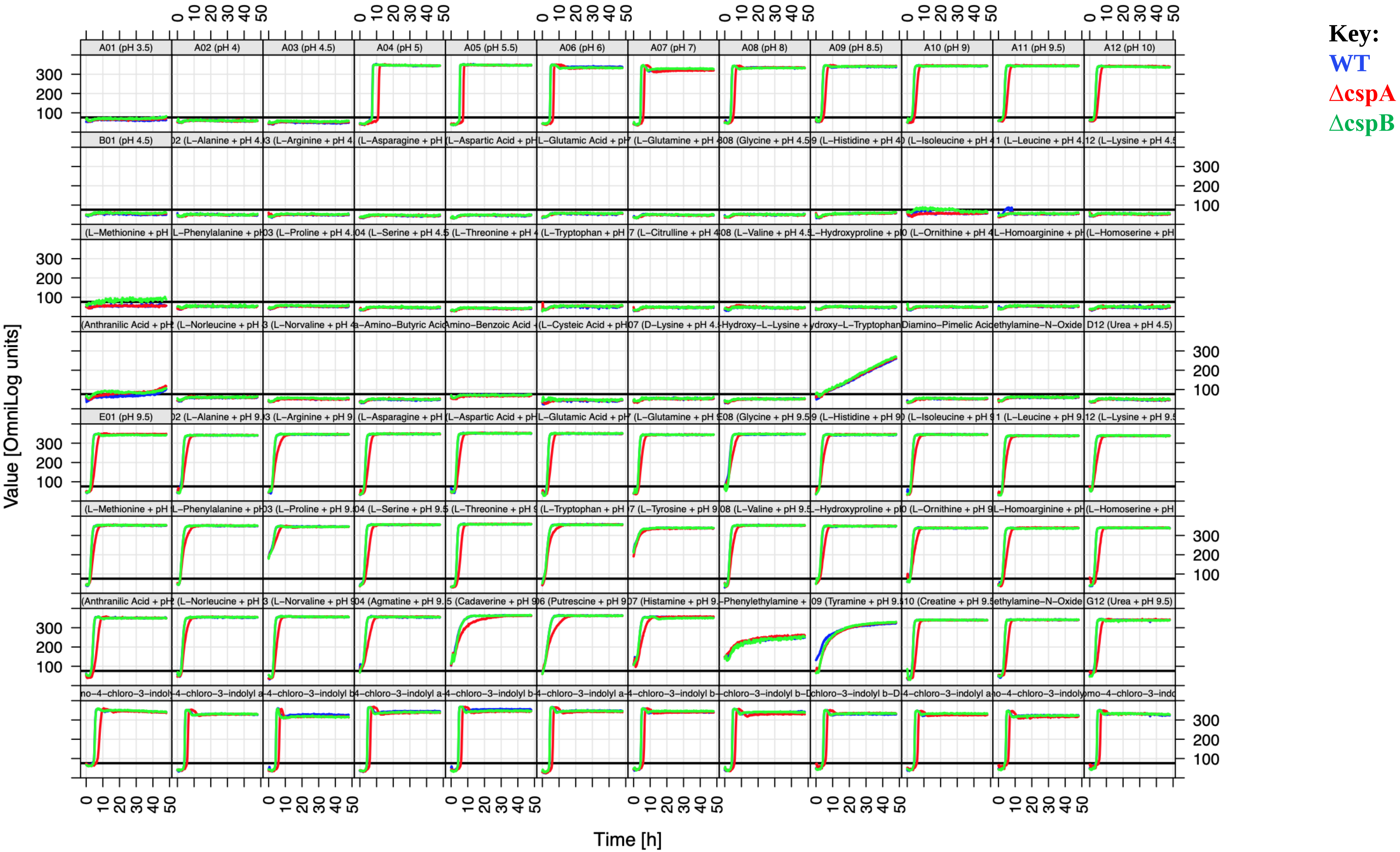

Supplementary Figure S4F: Opm generated graphs for PM10 (pH) generated using LMNC318 and its *csp* deletion mutants

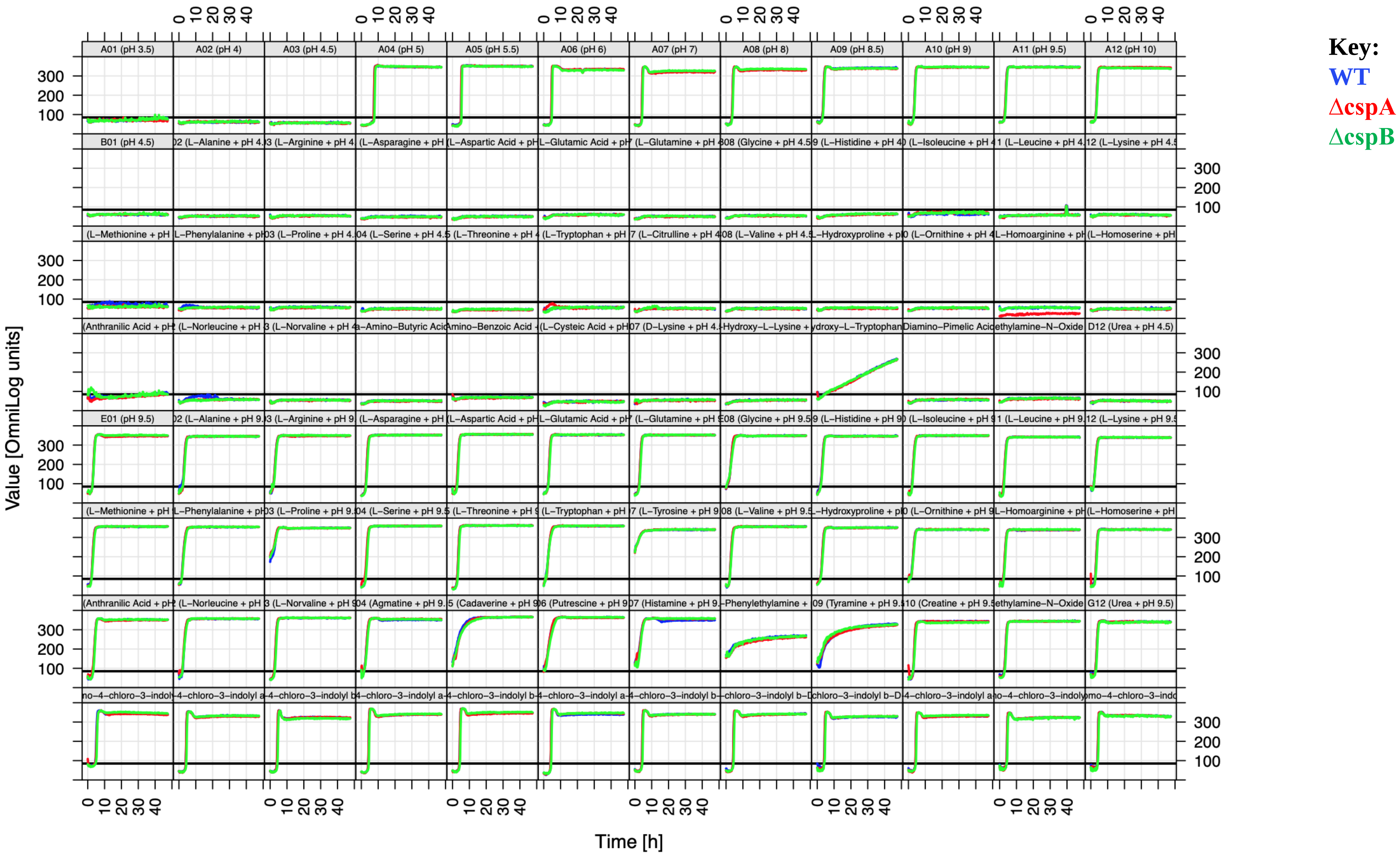

Supplementary Figure S4G: Opm generated graphs for PM10 (pH) generated using LMNC326 and its *csp* deletion mutants
