# Supplementary material for: Deciphering the global roles of Cold shock proteins in Listeria monocytogenes nutrient metabolism and stress tolerance
Source: Front Microbiol. 2022 Dec 20;13:1057754. doi: 10.3389/fmicb.2022.1057754 (PMC9808409; doi:10.3389/fmicb.2022.1057754)
Supplement: Supplementary file 3 [file Data_Sheet_2.docx]

**Supplementary Table S1.** Strains and plasmids used in this study

| **Strain ID** | **Description ^a^** | **Reference** |
| --- | --- | --- |
| EGDe **^b^** | Reference strain, LII, serotype 1/2a, CC9 | Glaser et al., 2001 |
| N1546 | 2011 Swiss listeriosis outbreak, LII, serotype 1/2a, CC8 | Hächler et al., 2013 |
| N2306 | 2013-2014 Swiss listeriosis outbreak, LI, serotype 4b, CC4 | Stephan et al., 2015 |
| LL195 | 1983 Swiss listeriosis outbreak, LI, serotype 4b, CC1 | Bille, 1990 |
| N16-0044 | 2016 Swiss listeriosis outbreak, LI, serotype 4b, CC6 | Althaus et al., 2017 |
| LMNC318 | Ruminant listeriosis, LIII, serotype 4a/4c, CC70 | Dreyer et al., 2016 |
| LMNC326 | Ruminant listeriosis, LIII, serotype 4a/4c, CC70 | Dreyer et al., 2016 |
| J5051 | *Listeria innocua* used as a negative control | Guldimann *et al*., 2015 |
| **∆*csp* strains** | | |
| EGDe_*ΔcspA* | EGDe *cspA* (*lmo1364*) deletion | Schmid et al., 2009 |
| EGDe_*ΔcspB* | EGDe *cspB* (*lmo2016)* deletion | Schmid et al., 2009 |
| EGDe_*ΔcspD* | EGDe *cspD* (*lmo1879*) deletion | Schmid et al., 2009 |
| EGDe_*ΔcspAB* | EGDe *cspA* and *B* deletions | Schmid et al., 2009 |
| EGDe_*ΔcspAD* | EGDe *cspA* and *D* deletions | Schmid et al., 2009 |
| EGDe_*ΔcspBD* | EGDe *cspB* and *D* deletions | Schmid et al., 2009 |
| *EGDe_ΔcspABD* **^b^** | EGDe *cspA, B,* and *D* deletions | Schmid et al., 2009 |
| EGDe_*ΔcspA*::pPL2-*cspA* | EGDe *cspA* deletion complemented with pPL2-*cspA* | Muchaamba et al., 2021b |
| EGDe_*ΔcspD*::pPL2-*cspD* | In-frame *cspD* deletion complemented with pPL2-*cspA* | Muchaamba et al., 2021b |
| *EGDe_ΔcspABD*::pPL2-*cspA* | In-frame *cspA, B,* and *D* deletions complemented with pPL2-*cspA* | Muchaamba et al., 2021b |
| *EGDe_ΔcspABD*::pPL2-*cspB* | In-frame *cspA, B,* and *D* deletions complemented with pPL2-*cspB* | Muchaamba et al., 2021b |
| *EGDe_ΔcspABD*::pPL2-*cspD* | In-frame *cspA, B,* and *D* deletions complemented with pPL2-*cspD* | Muchaamba et al., 2021b |
| N1546_*ΔcspA* | N1546 *cspA* deletion | This study |
| N1546_*ΔcspB* | N1546 *cspB* deletion | This study |
| N1546_*ΔcspAB* | N1546 *cspA* and *B* deletions | This study |
| N2306_*ΔcspA* | N2306 *cspA* deletion | This study |
| N2306_*ΔcspB* | N2306 *cspB* deletion | This study |
| N2306_*ΔcspAB* | N2306 *cspA* and *B* deletions | This study |
| LL195_*ΔcspA* | LL195 *cspA* deletion | This study |
| LL195_*ΔcspB* | LL195 *cspB* deletion | This study |
| LL195_*ΔcspAB* | LL195 *cspA* and *B* deletions | This study |
| N16-0044_*ΔcspA* | N16-0044 *cspA* deletion | This study |
| N16-0044_*ΔcspB* | N16-0044 *cspB* deletion | This study |
| N16-0044_*ΔcspAB* | N16-0044 *cspA* and *B* deletions | This study |
| LMNC318_*ΔcspA* | LMNC318 *cspA* deletion | This study |
| LMNC318_*ΔcspB* | LMNC318 *cspB* deletion | This study |
| LMNC318_*ΔcspAB* **^c^** | LMNC318 *cspA* and *B* deletions | This study |
| LMNC326_*ΔcspA* | LMNC326 *cspA* deletion | This study |
| LMNC326_*ΔcspB* | LMNC326 *cspB* deletion | This study |
| LMNC326_*ΔcspAB* **^c^** | LMNC326 *cspA* and *B* deletions | This study |
| **Plasmids** | | |
| pKSV7 | Plasmid vector | Smith & Youngman, 1992 |
| pKSV7-*ΔcspA* | pKSV7 with Δ*cspA* deletion construct | This study |
| pKSV7-*ΔcspB* | pKSV7 with Δ*cspB* deletion construct | This study |
| pKSV7-*ΔcspD* | pKSV7 with Δ*cspD* deletion construct | This study |
| pPL2 | Plasmid vector | Lauer et al., 2002 |
| pPL2-*cspA* | pPL2 with *cspA* sequence and 5’ flanking region | Muchaamba et al., 2021b |
| pPL2-*cspB* | pPL2 with *cspB* sequence and 5’ flanking region | Muchaamba et al., 2021b |
| pPL2-*cspD* | pPL2 with *cspD* sequence and 5’ flanking region | Muchaamba et al., 2021b |

^a^L: lineage, CC: clonal complex. ^b^Used in transcriptomics experiments. ^c^Strains not used in PM assays**.**

**Supplementary Table S2.** Validation of PM11-20 data **^a^**

| **Antibiotics (µg)** | **Diameter**  **(mm)** | **N1546** | | **LL195** | | **LMNC326** | | **EGDe** | | |
| --- | --- | --- | --- | --- | --- | --- | --- | --- | --- | --- |
|  |  | **WT** | **∆*cspAB*** | **WT** | **∆*cspAB*** | **WT** | **∆*cspAB*** | **WT** | **∆*cspAB*** | **∆*cspABD*** |
| Ampicillin (10) | Mean | 33.0 | 38.0 | 27.7 | 43.0 | 26.0 | 35.0 | 38.0 | 33.0 | 42.0 |
|  | SD | 1.4 | 2.8 | 0.6 | 7.1 | 0.0 | 4.2 | 8.7 | 1.4 | 5.7 |
| Trimethoprime (15) | Mean | 36.5 | 36.0 | 33.0 | 41.0 | 34.0 | 37.0 | 36.0 | 36.3 | 40.0 |
|  | SD | 2.1 | 0.0 | 1.4 | 4.2 | 2.0 | 1.4 | 5.7 | 1.5 | 0.0 |
| Polymixin B (300U) | Mean | 11.0 | 11.0 | 10.5 | 18.5 | 6.5 | 11.5 | 12.5 | 14.0 | 15.7 |
|  | SD | 1.4 | 1.4 | 2.1 | 2.1 | 0.7 | 0.7 | 3.5 | 0.0 | 0.6 |
| Amoxi/ Clav (20/10) | Mean | 33.5 | 38.7 | 28.5 | 41.0 | 27.5 | 37.7 | 36.0 | 36.0 | 43.0 |
|  | SD | 0.7 | 1.2 | 2.1 | 4.2 | 0.7 | 1.5 | 8.5 | 0.0 | 1.4 |
| Neomycin (30) | Mean | 25.7 | 28.5 | 23.5 | 28.0 | 25.0 | 26.5 | 28.0 | 28.0 | 31.0 |
|  | SD | 0.6 | 2.1 | 0.7 | 0.0 | 1.4 | 0.7 | 2.8 | 0.0 | 1.4 |
| Nalidixic acid (30) | Mean | 0.0 | 0.0 | 0.0 | 10.5 | 0.0 | 0.0 | 0.0 | 0.0 | 0.0 |
|  | SD | 0.0 | 0.0 | 0.0 | 3.4 | 0.0 | 0.0 | 0.0 | 0.0 | 0.0 |
| Ciprofloxacin (5) | Mean | 25.5 | 26.0 | 23.5 | 25.0 | 23.5 | 22.5 | 23.0 | 20.5 | 30.5 |
|  | SD | 0.7 | 5.3 | 0.7 | 1.0 | 2.1 | 0.7 | 4.2 | 0.7 | 2.1 |
| Sulfamethoxazole () | Mean | 32.0 | 30.5 | 28.0 | 39.0 | 29.0 | 31.0 | 30.0 | 29.0 | 35.0 |
|  | SD | 5.7 | 9.2 | 5.7 | 7.1 | 7.1 | 7.1 | 14.1 | 9.9 | 7.1 |
| Azithromycin (15) | Mean | 30.5 | 30.0 | 24.5 | 38.0 | 24.0 | 28.3 | 30.0 | 31.5 | 34.5 |
|  | SD | 2.1 | 0.0 | 0.7 | 2.8 | 0.0 | 2.5 | 3.6 | 0.7 | 2.1 |
| Chloraphenicol (30) | Mean | 25.5 | 26.0 | 23.5 | 26.0 | 23.5 | 15.0 | 23.0 | 26.5 | 26.0 |
|  | SD | 0.7 | 2.8 | 0.7 | 2.8 | 0.7 | 1.4 | 4.2 | 0.7 | 2.0 |
| Tetracycline (30) | Mean | 28.5 | 35.0 | 27.0 | 35.5 | 26.0 | 33.0 | 26.7 | 31.5 | 32.0 |
|  | SD | 0.7 | 1.4 | 1.4 | 0.7 | 0.0 | 1.4 | 4.2 | 4.9 | 2.8 |
| Streptomycin (10) | Mean | 18.5 | 19.5 | 13.7 | 21.0 | 18.0 | 19.5 | 20.5 | 25.5 | 25.0 |
|  | SD | 2.1 | 0.7 | 4.0 | 1.4 | 0.0 | 0.7 | 3.5 | 2.1 | 4.2 |
| Vancomycin (30) | Mean | 24.7 | 26.0 | 22.5 | 30.5 | 23.0 | 26.0 | 26.0 | 27.0 | 28.0 |
|  | SD | 1.2 | 0.0 | 0.7 | 0.7 | 1.4 | 0.0 | 3.5 | 1.4 | 0.0 |
| Clindamycin (2) | Mean | 5.0 | 14.5 | 2.7 | 34.5 | 2.7 | 17.5 | 12.5 | 21.5 | 19.0 |
|  | SD | 7.1 | 2.1 | 4.6 | 2.1 | 4.6 | 0.7 | 0.7 | 0.7 | 1.8 |
| Erythromycin (5) | Mean | 33.0 | 35.0 | 29.0 | 40.0 | 30.0 | 36.0 | 33.0 | 35.5 | 36.5 |
|  | SD | 1.4 | 1.4 | 1.4 | 0.0 | 0.0 | 2.8 | 3.6 | 0.7 | 0.7 |
| Penicillin (10 U) | Mean | 29.0 | 34.5 | 27.0 | 40.0 | 28.3 | 35.5 | 32.7 | 32.0 | 38.0 |
|  | SD | 1.4 | 0.7 | 1.4 | 2.8 | 2.1 | 0.7 | 3.1 | 2.8 | 0.0 |
| Rifampin (5) | Mean | 29.0 | 27.5 | 25.5 | 29.0 | 25.5 | 28.0 | 33.0 | 38.0 | 39.0 |
|  | SD | 1.4 | 0.7 | 0.7 | 1.4 | 0.7 | 0.0 | 1.4 | 2.8 | 1.0 |
| Cefuroxime (30) | Mean | 0.0 | 29.0 | 8.3 | 42.0 | 0.0 | 28.5 | 21.5 | 30.0 | 31.0 |
|  | SD | 0.0 | 1.4 | 10.2 | 5.7 | 0.0 | 0.7 | 4.9 | 2.8 | 4.2 |
| Cefoxitin (30) | Mean | 0.0 | 22.0 | 2.7 | 39.0 | 2.0 | 21.5 | 4.8 | 15.5 | 18.3 |
|  | SD | 0.0 | 2.8 | 4.6 | 1.4 | 3.5 | 0.7 | 5.5 | 0.7 | 5.9 |
| Amikacin (30) | Mean | 25.5 | 27.0 | 23.5 | 29.5 | 26.5 | 28.0 | 29.0 | 31.5 | 31.0 |
|  | SD | 0.7 | 1.4 | 0.7 | 0.7 | 2.1 | 0.0 | 4.2 | 3.5 | 4.2 |
| Kanamycin (30) | Mean | 28.5 | 28.5 | 25.5 | 32.5 | 26.5 | 29.5 | 30.5 | 34.5 | 33.3 |
|  | SD | 0.7 | 0.7 | 0.7 | 2.1 | 0.7 | 0.7 | 3.5 | 2.1 | 1.2 |
| Gentamicin (10) | Mean | 26.0 | 29.0 | 25.5 | 31.0 | 26.5 | 29.5 | 29.0 | 32.0 | 32.3 |
|  | SD | 0.0 | 1.4 | 0.7 | 1.4 | 2.1 | 0.7 | 4.2 | 2.8 | 1.5 |
| Cephalothin (30) | Mean | 25.0 | 30.5 | 25.0 | 42.0 | 25.0 | 32.0 | 28.7 | 34.0 | 33.5 |
|  | SD | 1.4 | 0.7 | 1.4 | 0.0 | 1.0 | 2.8 | 1.2 | 0.0 | 2.1 |
| Spectinomycin (100) | Mean | 28.0 | 30.0 | 26.0 | 36.0 | 26.0 | 34.0 | 32.0 | 37.0 | 36.0 |
|  | SD | 0.0 | 0.0 | 0.0 | 0.0 | 0.0 | 0.0 | 0.0 | 1.4 | 0.0 |
| Spiramycin (100) | Mean | 28.0 | 31.0 | 25.0 | 39.0 | 28.5 | 33.0 | 32.0 | 34.0 | 35.0 |
|  | SD | 0.0 | 0.0 | 0.0 | 0.0 | 7.8 | 0.0 | 0.0 | 0.0 | 0.0 |

**^a^** MIC (µg/ml). Reduced and increased sensitivity compared to its WT.

**Supplementary Table S3**. Genomes used in this study

| **Strain ID** | **Source** | **ST^a^** | **CC^b^** | **Serotypes** | **Lineage** | **Reference** | **Accession number** |
| --- | --- | --- | --- | --- | --- | --- | --- |
| LL195 | Vacherin Montd’or cheese | ST1 | CC1 | 4b | I | Bille, 1990 | HF558398 |
| N11-2292 | human listeriosis | ST1 | CC1 | 4b | I | Althaus et al., 2014 | JABYYE000000000 |
| N12-0605 | Meat / Meat product | ST727 | CC1 | 4b | I | Ebner et al., 2015 | JABYXR000000000 |
| N12-1339 | Meat / Meat product | ST746 | CC1 | 4b | I | Ebner et al., 2015 | JABYXM000000000 |
| N12-1996 | Milk / Milk product | ST1 | CC1 | 4b | I | Ebner et al., 2015 | JABYXD000000000 |
| N13-0047 | Milk / Milk product | ST1 | CC1 | 4b | I | Ebner et al., 2015 | JABYWZ000000000 |
| N11-2747 | human listeriosis | ST1 | CC1 | 4b | I | Althaus et al., 2014 | JABYYC000000000 |
| N12-0341 | human listeriosis | ST1 | CC1 | 4b | I | Althaus et al., 2014 | JABYXY000000000 |
| N12-0551 | human listeriosis | ST1 | CC1 | 4b | I | Althaus et al., 2014 | JABYXU000000000 |
| Scott A | Human listeriosis | ST290 | CC2 | 4b | I | Fleming et al., 1985 | CM001159 |
| N13-1054 | human listeriosis | ST1285 | CC2 | 4b | I | Althaus et al., 2014 | QYED00000000 |
| N12-0973 | Meat / Meat product | ST2 | CC2 | 4b | I | Ebner et al., 2015 | JABYXO000000000 |
| N11-1846 | Meat / Meat product | ST724 | CC2 | 4b | I | Ebner et al., 2015 | JABYYG000000000 |
| N12-0432 | Meat / Meat product | ST2 | CC2 | 4b | I | Ebner et al., 2015 | JABYXX000000000 |
| N12-0466 | Meat / Meat product | ST2 | CC2 | 4b | I | Ebner *et al*., 2015 | JABYXW000000000 |
| N2306 | Ready-to-eat salads | ST4 | CC4 | 4b | I | Stephan et al., 2015 | CP011004 |
| N12-0320 | human listeriosis | ST4 | CC4 | 4b | I | Althaus et al., 2014 | JABYXZ000000000 |
| N12-0794 | human listeriosis | ST4 | CC4 | 4b | I | Althaus et al., 2014 | JABYXQ000000000 |
| N13-2107 | Meat / Meat product | ST4 | CC4 | 4b | I | Ebner et al., 2015 | QYDL00000000 |
| N12-1772 | Milk / Milk product | ST682 | CC4 | 4b | I | Ebner et al., 2015 | JABYXH000000000 |
| N11-2675 | human listeriosis | ST1063 | CC5 | 1/2b | I | Althaus et al., 2014 | QYHF00000000 |
| N16-0044 | Meat pâté | ST6 | CC6 | 4b | I | Althaus et al., 2017 | CP035187 |
| N12-1387 | human listeriosis | ST6 | CC6 | 4b | I | Althaus et al., 2014 | JABYXL000000000 |
| N11-2801 | human listeriosis | ST6 | CC6 | 4b | I | Althaus et al., 2014 | JABYYB000000000 |
| N11-1850 | Milk / Milk product | ST1290 | CC217 | 4b | I | Ebner *et al*., 2015 | QYIA00000000 |
| N12-1608 | human listeriosis | ST224 | CC224 | 1/2b | I | Althaus *et al*., 2014 | JABYZR000000000 |
| H34 | Human listeriosis | ST489 | CC489 | 1/2b | I | Muchaamba et al., 2018 | CP020774 |
| Lm10403S | Human listeriosis | ST85 | CC7 | 1/2a | II | Edman et al., 1968 | NC_017544 |
| N1546 | Imported cooked ham | ST8 | CC8 | 1/2a | II | Hächler et al., 2013 | CP013724 |
| N11-1617 | Meat / Meat product | ST8 | CC8 | 1/2a | II | Ebner et al., 2015 | JABZDM000000000 |
| N11-1649 | Meat / Meat product | ST743 | CC8 | 1/2a | II | Ebner et al., 2015 | JABZDL000000000 |
| N11-1584 | human listeriosis | ST1295 | CC8 | 1/2a | II | Althaus et al., 2014 | JABZAJ000000000 |
| N11-1346 | Human listeriosis | ST673 | CC8 | 1/2a | II | Althaus *et al*., 2014 | JABZDQ000000000 |
| EGDe | Rabbits | ST35 | CC9 | 1/2a | II | Glaser et al., 2001 | NC_003210.1 |
| N11-1514 | Meat / Meat product | ST9 | CC9 | 1/2c | II | Ebner et al., 2015 | JABYZD000000000 |
| N12-1921 | Plant associated | ST9 | CC9 | 1/2c | II | Ebner et al., 2015 | JABYYW000000000 |
| N11-1837 | human listeriosis | ST9 | CC9 | 1/2a | II | Althaus et al., 2014 | JABZBE000000000 |
| N12-0486 | human listeriosis | ST9 | CC9 | 1/2c | II | Althaus et al., 2014 | JABYZC000000000 |
| N13-0001 | human listeriosis | ST9 | CC9 | 1/2c | II | Althaus et al., 2014 | JABYYS000000000 |
| Lm3136 | Tomme cheese | ST18 | CC18 | 1/2a | II | Bille et al., 2006 | CP013723 |
| N11-2183 | Plant associated | ST20 | CC20 | 1/2a | II | Ebner et al., 2015 | JABZDF000000000 |
| Lm3163 | Tomme cheese | ST26 | CC26 | 1/2a | II | Bille et al., 2006 | CP013722 |
| N11-1515 | Milk product | ST29 | CC29 | 1/2a | II | Ebner et al., 2015 | JABZDO000000000 |
| N05-195**^c^** | Meat / Meat product | ST31 | CC31 | 1/2a | II | Ebner et al., 2015 | QYIT00000000 |
| N11-1905 | Meat / Meat product | ST121 | CC121 | 1/2a | II | Ebner *et al*., 2015 | JABZDG000000000 |
| N12-1024 | Meat / Meat product | ST121 | CC121 | 1/2a | II | Ebner *et al*., 2015 | JABZCE000000000 |
| N13-0119 | human listeriosis | ST121 | CC121 | 1/2a | II | Althaus *et al*., 2014 | JABZAK000000000 |
| N12-0367 | human listeriosis | ST121 | CC121 | 1/2a | II | Althaus *et al*., 2014 | JABZAE000000000 |
| N842_15 | Human prosthetic joint | ST412 | CC412 | 1/2a | II | Muchaamba et al., 2020 | CP046361 |
| N843_10 | Human prosthetic joint | ST412 | CC412 | 1/2a | II | Muchaamba et al., 2020 | CP046362 |
| N12-1273 | Human listeriosis | ST412 | CC412 | 1/2a | II | Althaus *et al*., 2014 | QYFZ00000000 |
| WSLC1019 | Animal isolate | ST130 | CC69 | 4c | III | ATCC 19116 | CP013286 |
| LMNC318 | Ruminant listeriosis | ST70 | CC70 | 4a/4c | III | Dreyer et al., 2016 | ERS1324346 |
| LMNC326 | Ruminant listeriosis | ST70 | CC70 | 4a/4c | III | Dreyer et al., 2016 | ERS1324347 |
| WLSC1020 | Animal isolate | ST71 | CC131 | 4a | III | ATCC 19114 | NZ_CP013287 |
| F2365 | Genome control | ST1 | CC1 | 4b | I | Nelson et al., 2004 | NC_002973 |

**^a^**ST: sequence type; **^b^**CC: clonal complex. **^c^**Also known as N14-195.

**Supplementary Table S4**. RT-PCR Primers used in this study

| **Primer** | **Sequence** | **Reference** |
| --- | --- | --- |
| *cspA* | Fwd: AACATGGAACAAGGTACAG  Rev: GTTGGCCTTCTTCAACG | This study |
| *cspB* | Fwd: CAAACAGGTACAGTTAAATGGTTTA  Rev: ACGATTTCAAATTCAACGCTTTGA | This study |
| *cspD* | Fwd: TACGGTTTTATCGAATCAGAC  Rev: ACGTTAGCTGCTTGAG | This study |
| *rhaB* | Fwd: CTGTACCGGAGACGATT  Rev: CTGAAGGTCCCGCAAA | This study |
| *rhaD* | Fwd: AAACTTCTGCTGCTTTC  Rev: GTCACGCTACTCATTTG | This study |
| *rhaR* | Fwd: CCTAGAATGGCTAACGAGT  Rev: CTTCGAGCATGGTGGA | This study |
| *16S rRNA* | Fwd: CTTCCGCAATGGACGAAAGT  Rev: CTCATCGTTTACGGCGTG | Eshwar et al., 2017 |

**Supplementary Table S10**. Hierarchical trends for Csp importance on selected C-source

| **C-source** | **CspA** | **CspB** | **CspD** |
| --- | --- | --- | --- |
|  |  |  |  |
| Glycerol |  |  |  |
| Rhamnose |  |  |  |
| Arbutin |  |  |  |
| Thymidine |  |  |  |
| Dextrin |  |  |  |
| Palatinose |  |  |  |
| N-acetyl-D-glucosamine |  |  |  |
| N-acetyl-β-D-mannosamine |  |  |  |

**Key: Red>green>yellow**

# References

Althaus, D.; Lehner, A.; Brisse, S.; Maury, M.; Tasara, T.; and Stephan, R. 2014. Characterization of *Listeria monocytogenes* strains isolated during 2011-2013 from human infections in Switzerland. Foodborne. Pathog. Dis; 11: 753-758. doi: 10.1089/fpd.2014.1747.

Althaus, D.; Jermini, M.; Giannini, P.; Martinetti, G.; Reinholz, D.; Nüesch-Inderbinen, M.; Lehner, A.; Stephan, R. 2017. Local Outbreak of *Listeria monocytogenes* Serotype 4b Sequence Type 6 due to Contaminated Meat Pâté. Foodborne Pathog. Dis. 14, 219–222.

Bille, J. 1990. Epidemiology of human listeriosis in Europe with special reference to the Swiss outbreak, p 71-74 In Miller AJ, Smith JL, Somkuti GA, editors. Foodborne listeriosis. Elsevier, New York.

Bille, J.; Blanc, D.S.; Schmid, H.; Boubaker, K.; Baumgartner, A.; Siegrist, H.H.; Tritten, M.L.; Lienhard, R.; Berner, D.; Anderau, R.; et al. 2005. Outbreak of human listeriosis associated with tomme cheese in northwest Switzerland. Eurosurveillance 2006, 11, 11–12.

Dreyer, M.; Aguilar-Bultet, L.; Rupp, S.; Guldimann, C.; Stephan, R.; Schock, A.; Otter, A.; Schüpbach, G.; Brisse, S.; Lecuit, M.; et al. 2016. *Listeria monocytogenes* sequence type 1 is predominant in ruminant rhombencephalitis. Sci. Rep. 6, 36419.

Ebner, R.; Stephan, R.; Althaus, D.; Brisse, S.; Maury, M.; and Tasara, T. 2015. Phenotypic and genotypic characteristics of *Listeria monocytogenes* strains isolated during 2011-2014 from different food matrices in Switzerland. Food Control; 57: 321-326. doi: 10.1016/j.foodcont.2015.04.030

Edman, D.C.; Pollock, M.B.; Hall, E.R. 1968. *Listeria monocytogenes* L Forms I. Induction, Maintenance, and Biological Characteristics1. J. Bacteriol. 96, 352–357.

Fleming, D.W.; Holmes, M.B.; Audurier, A.; Cochi, S.L.; Macdonald, K.L.; Brondum, J.; Hayes, P.S.; Plikaytis, B.D.; Broome, C.V.; Reingold, A.L. 1985. Pasteurized Milk as a Vehicle of Infection in an Outbreak of Listeriosis. N. Engl. J. Med. 312, 404–407.

Glaser, P.; Frangeul, L.; Buchrieser, C.; Rusniok, C.; Amend, A.; Baquero, F.; Berche, P.; Bloecker, H.; Brandt, P.; Chakraborty, T.; et al. 2001. Comparative genomics of *Listeria* species. Science, 294, 849–852.

Guldimann, C.; Bärtschi, M.; Frey, J.; Zurbriggen, A.; Seuberlich, T.; and Oevermann, A. 2015. Increased spread and replication efficiency of *Listeria monocytogenes* in organotypic brain-slices is related to multilocus variable number of tandem repeat analysis (MLVA) complex. BMC Microbiol; 15: 134. doi: 10.1186/s12866-015-0454-0.

Hächler, H.; Marti, G.; Giannini, P.; Lehner, A.; Jost, M.; Beck, J.; Weiss, F.; Bally, B.; Jermini, M.; Stephan, R.; et al. 2013. Outbreak of Listerosis due to Imported Cooked Ham, Switzerland 2011. Eurosurveillance, 18. Available online: http://www.eurosurveillance.org/ViewArticle.aspx?ArticleId=20469 (accessed on 11 August 2020).

Lauer, P., Chow, M. Y., Loessner, M. J., Portnoy, D. A., and Calendar, R. 2002. Construction, characterization, and use of two *Listeria monocytogenes* site-specific phage integration vectors. J. Bacteriol. 184, 4177–4186. doi: 10.1128/jb.184.15.4177-4186.2002

Muchaamba, F.; Eshwar, A.K.; Von Ah, U.; Stevens, M.J.A.; Tasara, T. 2020. Evolution of *Listeria monocytogenes* During a Persistent Human Prosthetic Hip Joint Infection. Front. Microbiol. 11, 1726

Muchaamba, F.; Guldimann, C.; Tasara, T.; Mota, M.I.; Braga, V.; Varela, G.; Algorta, G.; Klumpp, J.; Jermini, M.; Stephan, R. 2017. Full-Genome Sequence of *Listeria monocytogenes* Strain H34, Isolated from a Newborn with Sepsis in Uruguay. Genome Announc. 5, e00544-17.

Nelson, KE.; Fouts, DE.; Mongodin, EF.; Ravel, J.; DeBoy, RT.; Kolonay, JF.; Rasko, DA.; Angiuoli, SV.; Gill, SR.; et al. 2004. Whole genome comparisons of serotype 4b and 1/2a strains of the food-borne pathogen *Listeria monocytogenes* reveal new insights into the core genome components of this species. Nucleic Acids Res. Apr 28;32(8):2386-95. doi: 10.1093/nar/gkh562.

Stephan, R.; Althaus, D.; Kiefer, S.; Lehner, A.; Hatz, C.; Schmutz, C.; Jost, M.; Gerber, N.; Baumgartner, A.; Hächler, H.; et al. 2015. Foodborne transmission of *Listeria monocytogenes* via ready-to-eat salad: A nationwide outbreak in Switzerland, 2013–2014. Food Control, 57, 14–17.

Tasara, T.; Ebner, R.; Klumpp, J.; Stephan, R. 2015. Complete genome sequence of *Listeria monocytogenes* N2306, a strain associated with the 2013-2014 listeriosis outbreak in Switzerland. Genome Announc 3(3):e00553-15. doi:10.1128/genomeA.00553-15.

Tasara, T.; Klumpp, J.; Bille, J.; Stephan, R. 2016. Genome sequences of *Listeria monocytogenes* strains responsible for cheese- and cooked ham product-associated Swiss listeriosis outbreaks in 2005 and 2011. Genome Announc 4(2):e00106-16. doi:10.1128/genomeA.00106-16.
